# Chemogenetic modulation of sensory neurons reveals their regulating role in melanoma progression

**DOI:** 10.1186/s40478-021-01273-9

**Published:** 2021-11-16

**Authors:** Pedro A. C. Costa, Walison N. Silva, Pedro H. D. M. Prazeres, Caroline C. Picoli, Gabriela D. A. Guardia, Alinne C. Costa, Mariana A. Oliveira, Pedro P. G. Guimarães, Ricardo Gonçalves, Mauro C. X. Pinto, Jaime H. Amorim, Vasco A. C. Azevedo, Rodrigo R. Resende, Remo C. Russo, Thiago M. Cunha, Pedro A. F. Galante, Akiva Mintz, Alexander Birbrair

**Affiliations:** 1 Departamento de Patologia, Universidade Federal de Minas Gerais, Belo Horizonte, MG Brasil; 2 Centro de Oncologia Molecular, Hospital Sirio-Libanes, Sao Paulo, SP Brasil; 3 Departamento de Bioquimica e Imunologia, Universidade Federal de Minas Gerais, Belo Horizonte, MG Brasil; 4 Departamento de Fisiologia e Biofísica, Universidade Federal de Minas Gerais, Belo Horizonte, MG Brasil; 5 Departamento de Farmacologia, Universidade Federal de Goiás, Goiânia, GO Brasil; 6 Centro das Ciências Biológicas e da Saúde, Universidade Federal do Oeste da Bahia, Barreiras, BA Brasil; 7 Departamento de Genetica, Ecologia e Evolucao, Universidade Federal de Minas Gerais, Belo Horizonte, MG Brasil; 8 Departamento de Farmacologia, Universidade de São Paulo, Ribeirão Preto, SP Brasil; 9 Department of Radiology, Columbia University Medical Center, New York, NY USA

**Keywords:** Sensory neurons, Tumor microenvironment, Melanoma, Neuronal activity, Chemogenetics

## Abstract

Sensory neurons have recently emerged as components of the tumor microenvironment. Nevertheless, whether sensory neuronal activity is important for tumor progression remains unknown. Here we used Designer Receptors Exclusively Activated by a Designer Drug (DREADD) technology to inhibit or activate sensory neurons’ firing within the melanoma tumor. Melanoma growth and angiogenesis were accelerated following inhibition of sensory neurons’ activity and were reduced following overstimulation of these neurons. Sensory neuron-specific overactivation also induced a boost in the immune surveillance by increasing tumor-infiltrating anti-tumor lymphocytes, while reducing immune-suppressor cells. In humans, a retrospective in silico analysis of melanoma biopsies revealed that increased expression of sensory neurons-related genes within melanoma was associated with improved survival. These findings suggest that sensory innervations regulate melanoma progression, indicating that manipulation of sensory neurons’ activity may provide a valuable tool to improve melanoma patients’ outcomes.

**Supplementary Information:**

The online version contains supplementary material available at 10.1186/s40478-021-01273-9.

## Introduction

Melanoma represents one of the leading causes of cancer-related deaths, being the most aggressive skin cancer type worldwide [124]. It emerges from molecularly altered melanocytes, which are the producers of melanin in the skin [48]. These cancer cells are embedded within the cutaneous microenvironment where they reside and interact dynamically with its constituents during disease progression [15, 54]. Understanding the interplay between the different components within the tumor microenvironment is crucial for the success of therapeutic applications, since each component can be influenced by the others, resulting in impacts on the cancer cells [9, 10, 45, 52, 88, 106]. The presence of individual nerve fibers within the tumor microenvironment was ignored for many years as they are difficult to detect in classical histology. For a long time, only large nerve trunks were detected within tumors, and they were always associated with perineural invasion of cancer cells, a process in which these cells grow and migrate along native passive tissue nerves [84]. Recently, a different phenomenon was described, by which the tumor itself is infiltrated pro-actively by newly developed peripheral nerve projections [32, 36, 71, 89, 108, 115, 116, 121, 154, 158].

To understand how peripheral innervations behave within the tumors, functional studies, in which intra-tumoral nerves were eliminated, have relied on the surgical or pharmacological manipulation of nerves. Each such strategy, however, has its disadvantages. Peripheral nerves contain mixtures of different nerve fiber types [41, 82], and therefore, surgical denervation of a peripheral nerve leads to the disruption of all the nerve fibers present within that specific nerve [101]. Consequently, the role of particular nerve projections in the tumor cannot be isolated, as other nerve fibers are also affected. On the other hand, pharmacological drugs cause systemic reactions in several organs and indirect effects on unexpected targets. Thus, achieving the neuronal type-specificity that is needed to understand the role that specific nerve fibers perform in the tumor microenvironment is difficult with these methods, and the observed outcomes could be due to the unwanted effects on other innervations in addition to the targeted neurons. Wherefore, conclusions drawn from studies based on surgical or pharmacological denervation may be imprecise. These are some of the reasons, in addition to tumor tissue specificity, for some of the ambiguity about the roles of specific nerve fibers in cancer behavior. Accordingly, contradictory reports have been published: while some studies have claimed that certain neuronal types promote cancer progression [57, 158], others concluded that they suppress tumorigenesis [32, 116].

Therefore, to study the role of specific innervations, these should be directly manipulated in a nerve-fiber-type-specific manner. Recently, this approach became possible with the advent of powerful genetically-based tools, that precisely allow the targeting and elimination of specific peripheral nerve fibers for studying their functions *in vivo* [13]. Our group showed that specific genetic depletion of sensory neurons promotes melanoma growth [108]. Nevertheless, genetic ablation of these innervations may result in the generation of a pro-inflammatory microenvironment, secondary to cell death in the site where the neurons were ablated (Männ et al. 2016; Christiaansen, Boggiatto, and Varga 2014; Bennett et al. 2005), which itself is strongly tumor growth promoting [49, 67], and can affect cancer cells’ behavior [50]. Thus, it remains unclear which facets of the sensory neuron-ablated tumor phenotype are due to the loss of sensory innervations, rather than indirect effects due to the local inflammation caused by the death of these neurons. To circumvent this issue, in the present study, we used chemogenetics, an experimental strategy that has empowered neuroscience studies [131, 147], to determine the precise role of sensory neurons in the regulation of melanoma progression. Designer Receptors Exclusively Activated by Designer Drugs (DREADDs) enable the silencing or overactivation of genetically defined neuronal populations upon binding to small-molecule designer drugs [119]. This approach allowed for highly selective and non-invasive modulation of sensory neurons’ activity in the tumor. Here, we revealed that silencing of sensory neurons’ activity, without ablating them, is sufficient to trigger increase in melanoma growth and in intra-tumoral new blood vessel formation. In contrast, chemogenetic stimulation of sensory neurons counteracted melanoma progression, by regulating tumoral growth, angiogenesis and immunosurveillance. Our results provide unequivocal evidence of the influence of sensory neurons in cancer progression.

## Materials and methods

### Animals

Generation of Nav1.8-Cre mice, in which Nav1.8 + sensory neurons express Cre recombinase, have been previously described. These animals were obtained from Infrafrontier (EMMA ID: 04 582). R26-LSL-hM4Di-DREADD (hM4Di) and CAG-LSL-hM3Dq-DREADD (hM3Dq) mice were purchased from the Jackson Laboratory (Jax) (Bar Harbor, ME).

To silence neuronal activity in sensory innervations in vivo, Nav1.8-Cre mice were crossed with R26-LSL-hM4Di-DREADD (hM4Di), a mouse line conditionally expressing a Gi-coupled engineered human muscarinic 4 receptor (hM4Di) [159]. hM4Di is a mutant G protein-coupled receptor which induces the canonical Gi pathway following binding to the pharmacologically inert drug clozapine-*N*-oxide (CNO). In Nav1.8-Cre + /hM4Di + mice, upon removal of the loxP-stop-loxP cassette by Cre recombination, the Gi-coupled hM4Di is expressed only in Nav1.8 + sensory neurons. Thus, sensory neuronal activity can be silenced by the administration of CNO. Nav1.8-Cre-/hM4Di + mice were used as controls.

To promote sensory neuron overactivation in vivo, Nav1.8-Cre mice were crossed with CAG-LSL-hM3Dq-DREADD (hM3Dq) animals, a mouse line conditionally expressing an evolved Gq protein-coupled receptor (hM3Dq), to generate Nav1.8-Cre + /hM3Dq + mice. In Nav1.8-Cre + /hM3Dq + animals, upon removal of loxP-stop-loxP cassette by Cre recombination, the Gq-coupled hM3Dq is expressed specifically in Nav1.8-sensory neurons. hM3Dq is a mutant G protein-coupled receptor which induces the canonical Gq pathway following the binding to CNO. Thus, sensory neuron firing can be chemically induced by administration of CNO. Nav1.8-Cre-/ hM3Dq + animals were used as controls.

All animal care and experimental procedures were approved by the Ethics Animal Care and Use Committee (CEUA), in accordance with the Guide for the Care and Use of Laboratory Animals from the Federal University of Minas Gerais. All colonies were housed in a pathogen-free animal facility of the Department of Pathology, UFMG, under controlled light cycle (12:12-h light/dark cycle) and fed ad libitum. Age-matched 8- to 12-week-old mice were used for all experiments. All experiments used mice heterozygous for both NaV1.8-Cre and DREADD receptors.

### Cell culture

Murine B16-F10 melanoma is a common cell line that naturally originated in melanin-producing epithelia of C57BL6 mice. These cells were originally obtained from ATCC (USA), and were used to study melanoma development in vivo. The cells were cultured in Dulbecco’s modified Eagle’s medium (DMEM) supplemented with 10% (v/v) fetal calf serum/2 mM L-glutamine/100 U/ml penicillin/100 μg/ml streptomycin. The cells have been tested and found negative for mycoplasma. Cells were cultured in a humidified atmosphere of 95% air and 5% (v/v) CO2 at 37 °C.

### Melanoma tumor implantation

B16-F10 cells were suspended in PBS and examined for viability using trypan blue staining. B16-F10 cells were used for transplantation only when more than 90% of cells were viable. For subcutaneous injection, the skin of all mice at an age of 8–12 weeks was shaved at the place of application. 1 × 10^5^ cells in 100 μL were injected subcutaneously into the right flank of each mouse and the growth of the tumors was monitored until sacrifice. Growth of the tumors was assessed over time with a caliper as previously reported [12]. For determination of tumor volume, tumor-bearing animals were anesthetized with isoflurane in O2 by manually restraining the mice and placing their heads in in-house-built nose cones. Tumors were removed 16 days after transplantation and weighted. Length (L) and width (W) were calculated to measure tumor volume (V) using the formula V = 0.5 × (L × W2) [40]. Tumor area was determined using calibrated photographs of each tumor using Fiji software®, version 1.53 (National Institute of Health, Bethesda, MD).

### CNO treatment

The DREADD ligand clozapine-N-oxide (CNO) (1 mg/kg in saline) (Sigma-Aldrich, St Louis, MO, USA) [7] was administered intra-peritoneally using a 25-gauge needle daily to test the effect of neuronal inhibition or activation on melanoma progression in Nav1.8-Cre + /hM4Di + and Nav1.8-Cre + /hM3Dq + animals, respectively. Control Nav1.8-Cre-/hM4Di + and Nav1.8-Cre-/hM3Dq + mice were similarly injected with CNO.

### Capsaicin-induced spontaneous behavior

To confirm sensory neurons inhibition efficiency, following acclimation, Nav1.8-Cre-/hM4Di + and Nav1.8-Cre + /hM4Di + mice were injected with an intra-plantar subcutaneous dose of 10 μl of capsaicin (1 μg/10 μl; Sigma-Aldrich). A video recording was taken for 5 min post-capsaicin injection. The time that the animals spent performing spontaneous behaviors of licking, lifting, and flicking the paw were measured for 5 min after injection of capsaicin from these videos.

### Immunohistochemistry and microscopy

Adult mice were deeply anesthetized with isoflurane and transcardially perfused with saline followed by 4% buffered paraformaldehyde (PFA, pH = 7.4). After dissection, B16F10 tumors were fixed overnight at 4 °C in 4% buffered paraformaldehyde, incubated overnight at 4 °C with 30% sucrose diluted in PBS, embedded and frozen in optimal cutting temperature compound (OCT, Tissue‐Tek). Embedded tumors were stored at − 80 °C. 20 μm cryosections were cut and blocked for 2 h in 3% BSA in PBS + 0.5% Triton and immunostained with the following antibodies: CD31‐PE (dilution 1:100) (BioLegend), Ki67 (dilution 1:100) (BD Biosciences), and anti‐Guinea pig‐AlexaFluor‐647 (1:1000) (Life Technologies) [10, 23]. After this, the sections were washed with PBS containing 4',6-diamidino-2-phenylindole (DAPI, 5 μg/ml, Invitrogen) and mounted using Dako fluorescence mounting medium (Dako, Santa Clara, CA). Stained tumor sections were imaged and analyzed by confocal microscopy using an inverted Zeiss LSM 880 confocal microscope (Oberkochen, Germany). CD31 area and the number of Ki67^+^ cells were quantified using Fiji software®, version 1.53 (National Institute of Health). Multiple random fields of each section were used for quantification.

### Tumor-infiltrating leukocytes immunophenotyping and intracellular cytokine measurement

Tumors, their draining lymph nodes and non-tumor draining lymph nodes were harvested. Tissues were macerated and filtrated trough cell strainers of 40 um (Falcon) to isolate the cells used for immunophenotyping. Cells were washed in phosphate-buffered saline (PBS), incubated with Live/Dead solution (Invitrogen), for dead cell exclusion, and with monoclonal antibodies, washed, fixed, and permeabilized (FoxP3 staining buffer set, eBioscience) according to manufacture's instructions. Antibodies are listed in Table 1. Acquisition was realized on a LSR-FORTESSA. For analyses, to exclude debris, combinations of fluorochromes was done, to remove doublets a forward scatter area (FSC-A) versus forward scatter height (FSC-H) gate was used, and then cells were gated in function of time versus FSC-A to avoid a possible interference of flux interruptions. Only live leukocytes were used using a Live/Dead gate versus CD45. We assessed different immune cell subpopulations based on molecular markers of each cell subset: CD4 + T cells (CD4^+^/CD3^+^), CD8 + T cells (CD8^+^/CD3^+^), γδ T cells (CD3^+^/CD4^−^/CD8^−^/TCRγδ^+^), NKT cells (CD3^+^/NK^+^), regulatory T cells (Foxp3^+^/CD4^+^/CD3^+^), NK cells (CD3^−^/NK^+^), neutrophils (CD11b^+^/CD11c^−^/Ly6C^−^/LyG6^+^), PMN/MDSCs (CD11b^+^/Ly6C^−^/LyG6^+^) and dendritic cells (CD11b^−^/CD11c^+^/Ly6C^−^/LyG6^−^). In each T-cell subset, frequencies of cells expressing checkpoint inhibitors CTLA-4 and PD1 were evaluated. Cytokine analyses in lymphocytes from the tumor microenvironment and lymph nodes were done using intracellular staining. Briefly, cells were isolated from tumor samples and lymph nodes and cultivated for 4 h at 37 °C in 10% FBS RPMI supplemented with 2 mM L-glutamine, 50 units/mL penicillin, and 50 μg/mL streptomycin, in the presence of Brefeldin A (ThermoFisher) and Monensin (ThermoFisher). Following this, cells were washed in FACS buffer and stained for cell surface markers. Cells were then fixed for 35 min at 4 °C with eBiosciences Cytofix/Cytoperm buffer and, subsequently, washed once in eBioscience Perm/Wash buffer. Then, cells were stained for 45 min at 4 °C with anti-IFN-γ and anti-IL-17 (Table 1) antibodies diluted in eBioscience Perm/Wash [78]. Cells were washed twice and the data was acquired. Ki67 is a nuclear factor transcript in the late G1, S, G2, and M of cell cycle, therefore marks proliferating cells [44, 128]. Thus, we evaluated proliferation in viable CD45 negative cells, suggesting tumoral proliferation. GraphPad Prism V7.0 (GraphPad software) and FlowJo V10.4.11 (TreeStar) were used for data analysis and graphic presentation.

**Table 1:** Antibodies used in flow cytometry

Antigen	*Fluorochrome*	Clone	Company
CD3	eFluor450	145-2C11	ThermoFhisher
CD8a	eFluor 450	53–6.7	ThermoFhisher
CD11c	eFluor 450	N418	ThermoFhisher
LIVE/DEAD	Acqua		ThermoFhisher
Streptavidin	Pacific Orange		ThermoFhisher
CD45	Super Bright 600	30-F11	ThermoFhisher
TCR gamma/delta	Super Bright 645	eBioGL3	ThermoFhisher
CD4	Alexa Fluor 488	GK1.5	ThermoFhisher
F4/80	FITC	BM8	Hycult
NK1.1	PE-eFluor 610	PK136	ThermoFhisher
CD8a	PerCP-Cyanine5.5	53–6.7	ThermoFhisher
Ly-6G	PerCP-eFluor 710	1A8-Ly6g	ThermoFhisher
IL-17A	PerCP-Cyanine5.5	eBio17B7	ThermoFhisher
CTLA-4	PE-Cyanine7	UC10-4B9	ThermoFhisher
FoxP3	Alexa Fluor 647	3G3	ThermoFhisher
CD3e	Cyanine5	500A2	ThermoFhisher
Ki67	AlexaFluor 700	SolA15	ThermoFhisher
PD-1	APC-eFluor 780	J43	ThermoFhisher
Ly-6C	APC-eFluor 780	HK1.4	ThermoFhisher
IFN-γ	APC-eFluor 780	XMG1.2	ThermoFhisher
CD11b	Biotin	M1/70	Biolegend

### Quantification of CGRP within tumors

Tumor samples from Nav1.8-Cre + /hM4Di + and Nav1.8-Cre + /hM3Dq + animals, as well as from their respective controls (Nav1.8-Cre-/hM4Di + and Nav1.8-Cre-/hM3Dq +) were analyzed to measure the amount of CGRP using commercially available Sandwich-CGRP ELISA kit purchased from Elabscience (Catalog # E-EL-M2744). Briefly, tumor pieces were weighed and then homogenized in PBS (0.01 M, pH = 7.4) with a glass homogenizer on ice. The homogenates were centrifuged for 5 min at 5000 × g at 4 ℃ to get the supernatant. ELISA of CGRP were performed according to manufacturer’s instructions. After ELISA, Optical Density (OD) was measured using Varioskan Flash (Thermo) set at 450 nm.

### In silico analysis

RNA sequencing count data of 103 Skin Cutaneous Melanoma (SKCM) patients was downloaded from The Cancer Genome Atlas (TCGA) repository (https://portal.gdc.cancer.gov/). Differential gene expression analyses were performed between samples of alive and dead patients (considering a 5-year interval) using DESeq2 [83]. We stratified patients in these two groups, alive or dead, based on their vital status in a 5-year interval of their tumor diagnosis (clinical data available at TCGA and curated by Liu et al. (2018) [85]. Genes with absolute log2(Fold-change) ≥ 1 and False Discovery Rate (FDR) adjusted *P*-value < 0.05 were considered differentially expressed. To identify biological processes associated with genes differentially expressed, we performed a Gene Ontology (GO) enrichment analysis using ShinyGO [42]. We used the STRING database [132] (parameters: full STRING network, considering only text-mining, databases and experiments interactions with score > 0.400, and only genes with 3 or more interactions) and Cytoscape (https://cytoscape.org/) to construct protein–protein interactions (PPIs) among our manually curated list of 34 gene related to sensory neurons selected based on the literature [29, 37, 51, 114, 141, 144]. The set of 18 genes showing at least two PPI interactions are shown. For the remaining analyses, RNA sequencing counts were first Transcripts Per Million (TPM)-normalized using a local R script. To identify a gene signature associated with SKCM cancer patient survival we used Reboot [31] with parameters "-B 100 -G 5 -P 0.3 -V 0.01". Briefly, Reboot finds genes associated with cancer patient prognosis using multivariate penalized Cox regression combined with a bootstrap approach. In the first step of Reboot, it produces regression coefficients (numerical values) that determine the contribution of each submitted gene to patients' survival. These coefficients may be positive or negative values indicating that high expression of a particular gene potentially contributes to worse or better prognosis, respectively. Once these coefficients are produced, Reboot then calculates the score of each patient (sample) as the sum of each gene coefficient multiplied by the corresponding gene expression level in that patient. Finally, when all patients' scores are calculated, we then stratify them into groups with high/low scores based on the median score of all patients to create the survival curve (Kaplan–Meier). For further information, see [31]. SCN10A box plots and survival curves were created using R (https://www.r-project.org/) scripts.

### Statistical analysis

Graphs were plotted using GraphPad Prism 7 (San Diego, CA). Shapiro‐Wilk normality test was performed, and unpaired *t* test was used to determine statistical significance.

## Results

### Chemogenetic inhibition of Nav1.8 + neurons accelerates melanoma progression

**Fig. 1:**
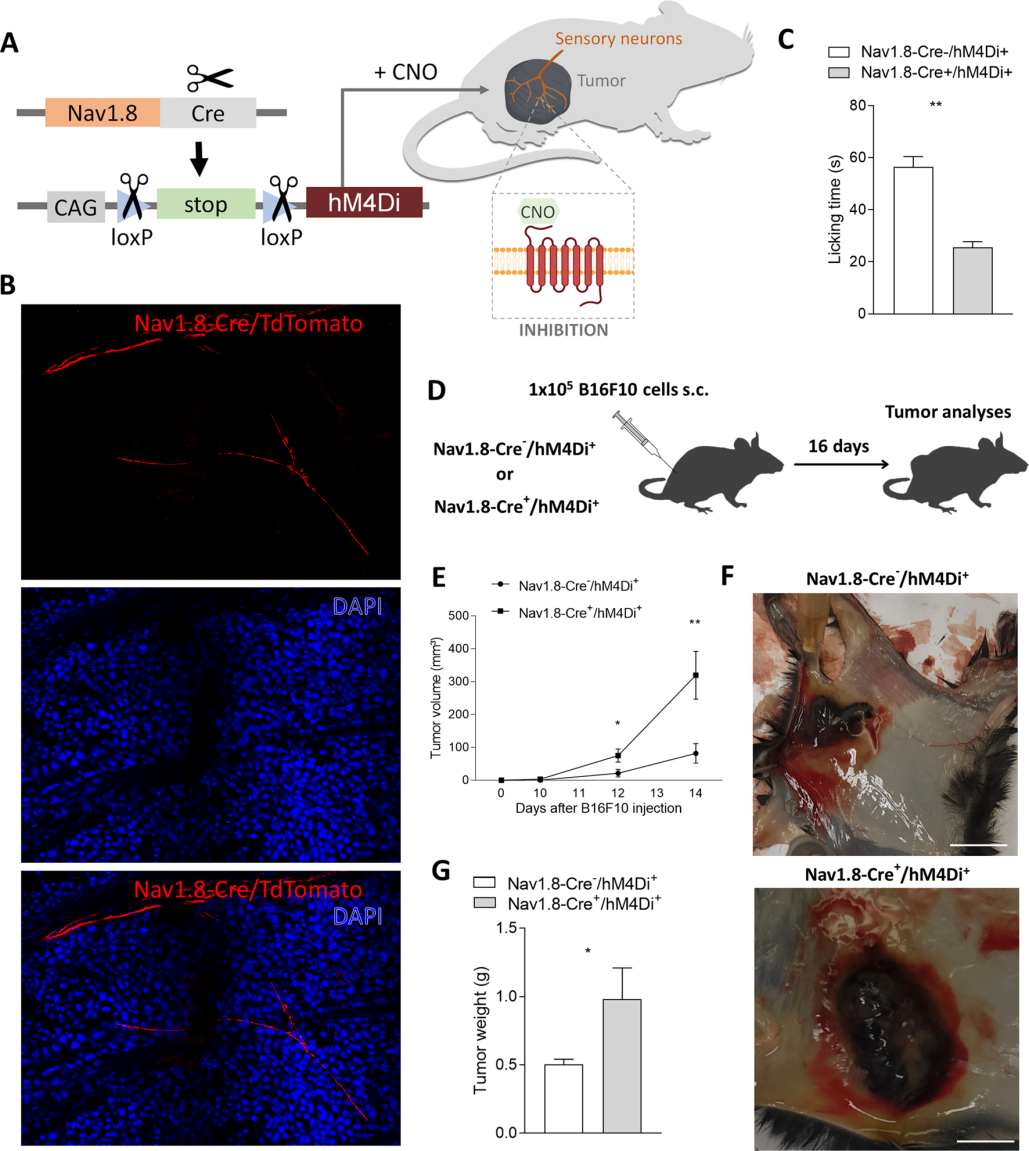
Chemogenetic inhibition of neuronal activity in sensory Nav1.8 + nerve fibers triggers melanoma growth. **A** Schematic diagram of the Nav1.8-Cre + /hM4Di + experimental mouse model. Cre recombinase directs the expression of hM4Di specifically to sensory neurons in those mice. After the administration of CNO to those mice, neuronal activity in sensory neurons is inhibited. **B** Tumor-infiltrating sensory neurons are targeted in Nav1.8-Cre mice. 1 × 10^5^ B16F10 melanoma cells were subcutaneously injected into Nav1.8-Cre/TdTomato mice, and tumor tissues were surgically removed 16 days later. Representative image of a Nav1.8-Cre/TdTomato mouse tumoral section with sensory nerve fibers infiltrating the tumor labelled with TdTomato fluorescence (red) and nuclei with DAPI (blue). **C** Capsaicin-induced spontaneous behavior test corroborates chemogenetic inhibition of sensory Nav1.8 + nerve fibers in Nav1.8‐Cre^+^/hM4Di^+^ mice after CNO treatment. Column charts show the licking time after capsaicin application of Nav1.8‐Cre^−^/hM4Di^+^ (n = 5) and Nav1.8‐Cre^+^/hM4Di^+^ (n = 5) animals. **D** Representation of the protocol for subcutaneous allograft melanoma growth. 1 × 10^5^ B16F10 melanoma cells were subcutaneously injected into Nav1.8‐Cre^−^/hM4Di + (n = 5) and Nav1.8‐Cre + /hM4Di + (n = 5) mice, followed by tumors removal for analysis after 16 days. CNO was daily intra-peritoneal injected at 1 mg/kg. **E** Development curve of tumor growth from Nav1.8‐Cre^−^/hM3Dq^+^ and Nav1.8‐Cre^+^/hM3Dq^+^. Tumor volumes were assessed over time with a caliper. **F** Representative macroscopic image of B16F10 melanoma after dissection, left panel (Nav1.8‐Cre^−^/hM4Di^+^) and right panel (Nav1.8‐Cre^+^/hM4Di^+^). **G** Tumor weight. (Nav1.8‐Cre^−^/hM4Di^+^: 0.50 ± 0.04 g; Nav1.8‐Cre^+^/hM4Di^+^: 0.98 ± 0.23 g). Data are shown as mean ± SEM. Unpaired t test (ns P > 0.05; *P < 0.05; **P < 0.01)

We have previously demonstrated that melanomas are infiltrated by Nav1.8 + sensory innervations, and that those tumors grow slower when these neurons are pharmacologically or genetically ablated [108]. However, these investigations were performed using Nav1.8-Cre + /DTA + mice, in which a diphtheria toxin fragment A is constitutively activated in Nav1.8 + sensory neurons, resulting in the toxin induced-death of these cells. Therefore, this technique lacks temporal control of neuronal ablation, and enables compensatory effects during the development of these animals. Importantly, the approach by which specific neurons are ablated from the tissue microenvironment is also limited because of the secondary consequences, such as inflammation, caused in the tissue where sensory neurons are eliminated. Thus, it remains unclear whether these damage-induced changes in the tissue may influence the observed cancer outcomes. Here, we applied a chemogenetic approach to specifically inhibit the activity of Nav1.8-expressing sensory neurons without killing these cells. We used DREADDs to specifically control sensory neuron activity. DREADDs are derived from different types of mutated muscarinic receptors that have been engineered to lose affinity to their endogenous ligand acetylcholine [5], but to gain responsiveness to a synthetic ligand, clozapine-N-oxide (CNO). Inhibitory DREADDs (hM4Di) elicit an intracellular cascade that results in the silencing of neuronal activity [113], without changing the number of innervations as previously reported [68]. This method allows for the selective silencing of specific types of neurons in vivo without physical manipulation or destruction in the tissue. DREADDs were expressed specifically in sensory neurons, using a transgenic murine approach: mice carrying the construct encoding for Cre-dependent expression of hM4Di were crossed to Nav1.8-Cre animals. In the resulting mice, Nav1.8-Cre + /hM4Di + , only Nav1.8 + sensory neurons expressed inhibitory DREADDs. As controls in this study, we used littermate mice carrying Cre-dependent hM4Di, but lacking the Cre gene (Nav1.8-Cre-/hM4Di +) (Fig. 1A). This allowed us to control for any potential side effects from CNO administration.

In order to ascertain that the expression of DREADD receptors was driven to intra-tumoral Nav1.8-expressing neurons in Nav1.8-Cre + /hM3Di + mice, we used the solid tumor model B16F10. We assessed tumor sections from melanoma grown in Nav1.8-Cre + /TdTomato + mice and detected Nav1.8 + neurons expressing TdTomato present within the tumor microenvironment (Fig. 1B). To validate sensory neuronal inhibition following daily CNO injection, we used a behavioral test to evaluate the sensitivity to capsaicin, confirming the silencing of sensory neurons, as previously described [2]. Indeed, Nav1.8-Cre + /hM4Di + animals spent less time (25.53 ± 2.27 s) licking their paws after intra‐plantar injection of capsaicin, compared to control animals (56.53 ± 3.92 s) (Fig. 1C). To analyze the effect of sensory neurons silencing on tumor growth, we subcutaneously transplanted B16F10 cells to the lower right flank of both inhibitory DREADD-expressing mice (Nav1.8-Cre + /hM4Di +) and their controls (Nav1.8-Cre-/hM4Di +). Following cancer cell injection, we treated the animals daily with CNO to induce sensory neuronal activity inhibition (controls were also treated with CNO) (Fig. 1D). After 14 days of continuous sensory inhibition, tumor volume was significantly enhanced in the sensory neuron-silenced mice when compared to the controls (tumor volume was increased from 82.1 ± 29.6 to 319.6 ± 72.8 mm^3^; Fig. 1E). After 16 days of repeated sensory inhibition, tumor weight was also significantly enhanced in the sensory neuron-silenced mice when compared to the controls (tumor weight was increased from 0.50 ± 0.04 to 0.98 ± 0.23 g; Fig. 1F, G). Animal weights were not affected by sensory inhibition (data not shown).

**Fig. 2:**
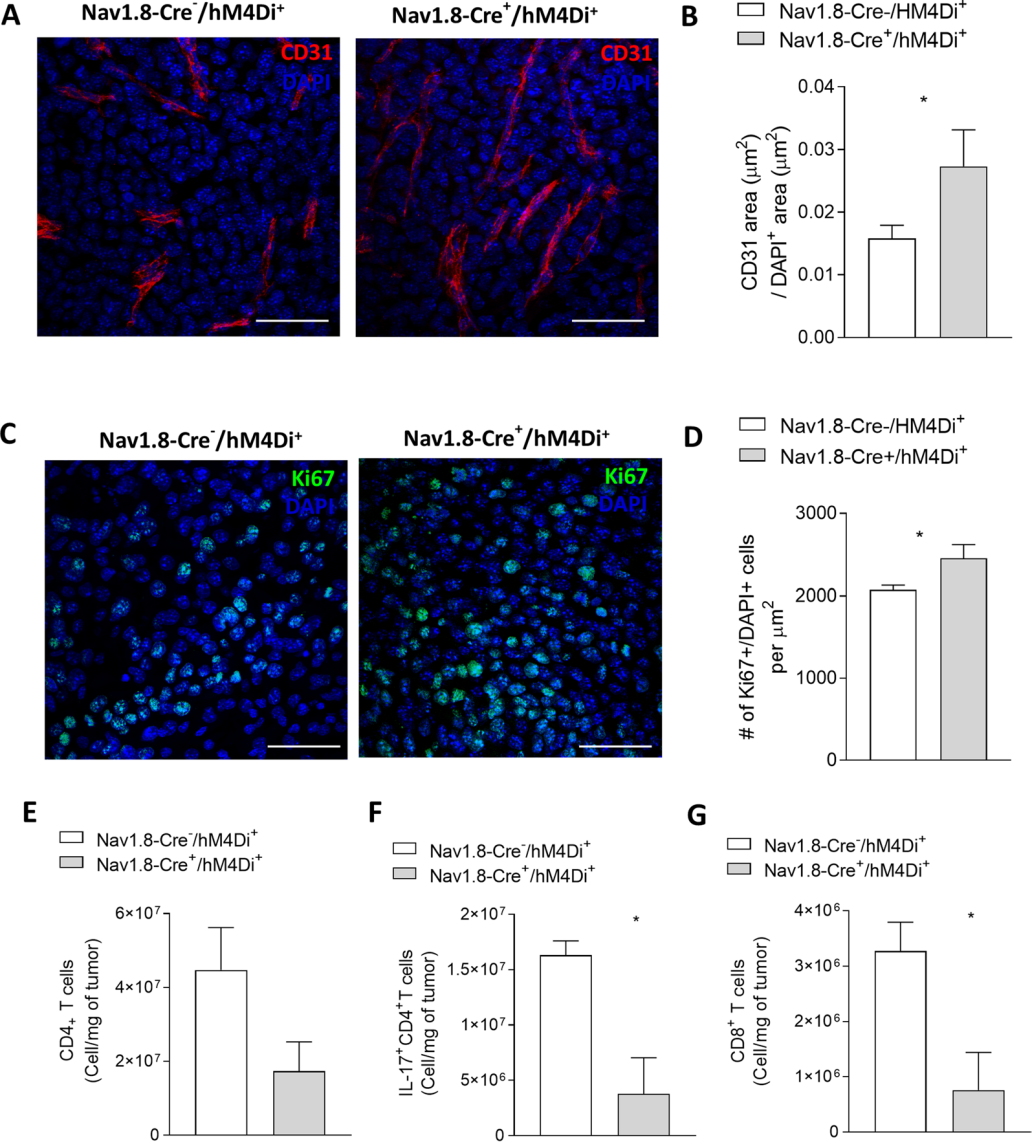
Chemogenetic inhibition of neuronal activity in sensory Nav1.8 + innervations increases intra-tumoral proliferation and angiogenesis, and blocks anti-tumoral immune response. 1 × 10^5^ B16F10 melanoma cells were subcutaneously injected into Nav1.8‐Cre^−^/hM4Di + (n = 5) and Nav1.8‐Cre + /hM4Di + (n = 5) mice, followed by tumors removal for analysis after 16 days. **A** Representative immunofluorescence images of tumors labelled for endothelial cells (CD31; red) to identify blood vessels and nuclei (DAPI; blue). **B** Quantification of angiogenesis in melanomas by blood vessel area. **C** Representative immunofluorescence images of tumors labelled for Ki67 (Ki67; green) to identify cell proliferation and nuclei (DAPI; blue). **D** Quantification of proliferation in melanomas by the counting of Ki67 + cells per μm^2^. Absolute number of CD4 + **E** and CD8 + **G** T cells from the melanomas of B16F10–inoculated mice. **F** Graph shows absolute numbers of CD4 + T cells producers of IL-17. IL-17 levels were measured in cells isolated from tumors of B16F10–inoculated Nav1.8-Cre^−^/hM4Di^+^ and Nav1.8-Cre^+^/hM4Di^+^ animals. Data are shown as mean ± SEM. Unpaired t test (ns *P* > 0.05; *P < 0.05)

Increase in neoangiogenesis within melanoma tumors is correlated with worse outcomes in these patients [111]. We detected, in melanoma-bearing animals with silenced sensory neurons, an enhancement in the intra‐tumoral blood vessels’ area (from 0.02 ± 0.00 to 0.03 ± 0.01 µm^2^) (Fig. 2A, B). Expression of Ki67 is used to determine the proliferation rate of malignant cancer cells [139], which is also associated with melanoma aggressiveness [76]. We found that genetic silencing of sensory innervations led to an increase in the proliferation rate within the melanoma (from 2074 ± 55.32 to 2454 ± 168.4 Ki67 + cells per μm^2^) (Fig. 2C, D). We also observed after inhibition of sensory neurons firing a decrease in tumor-infiltrating CD4 + T cells (from 4.47 × 10^7^ ± 1.15 × 10^7^ to 1.73 × 10^7^ ± 7.92 × 10^6^ cells per mg of tumor) (Fig. 2E), in special, in IL-17-producing CD4 + T cells (from 1.63 × 10^7^ ± 1.30 × 10^6^ to 3.77 × 10^6^ ± 3.27 × 10^6^ cells per mg of tumor) (Fig. 2F), and a decrease in melanoma-infiltrating CD8 + T cells (from 3.27 × 10^6^ ± 5.22 × 10^5^ to 7.62 × 10^5^ ± 6.78 × 10^5^ cells per mg of tumor) (Fig. 2G). Our results indicate that inhibition of neuronal activity in sensory neurons promotes melanoma tumor advancement.

### Chemogenetic activation of hM3Dq excitatory DREADD receptors in Nav1.8 + neurons promotes melanoma regression

**Fig. 3:**
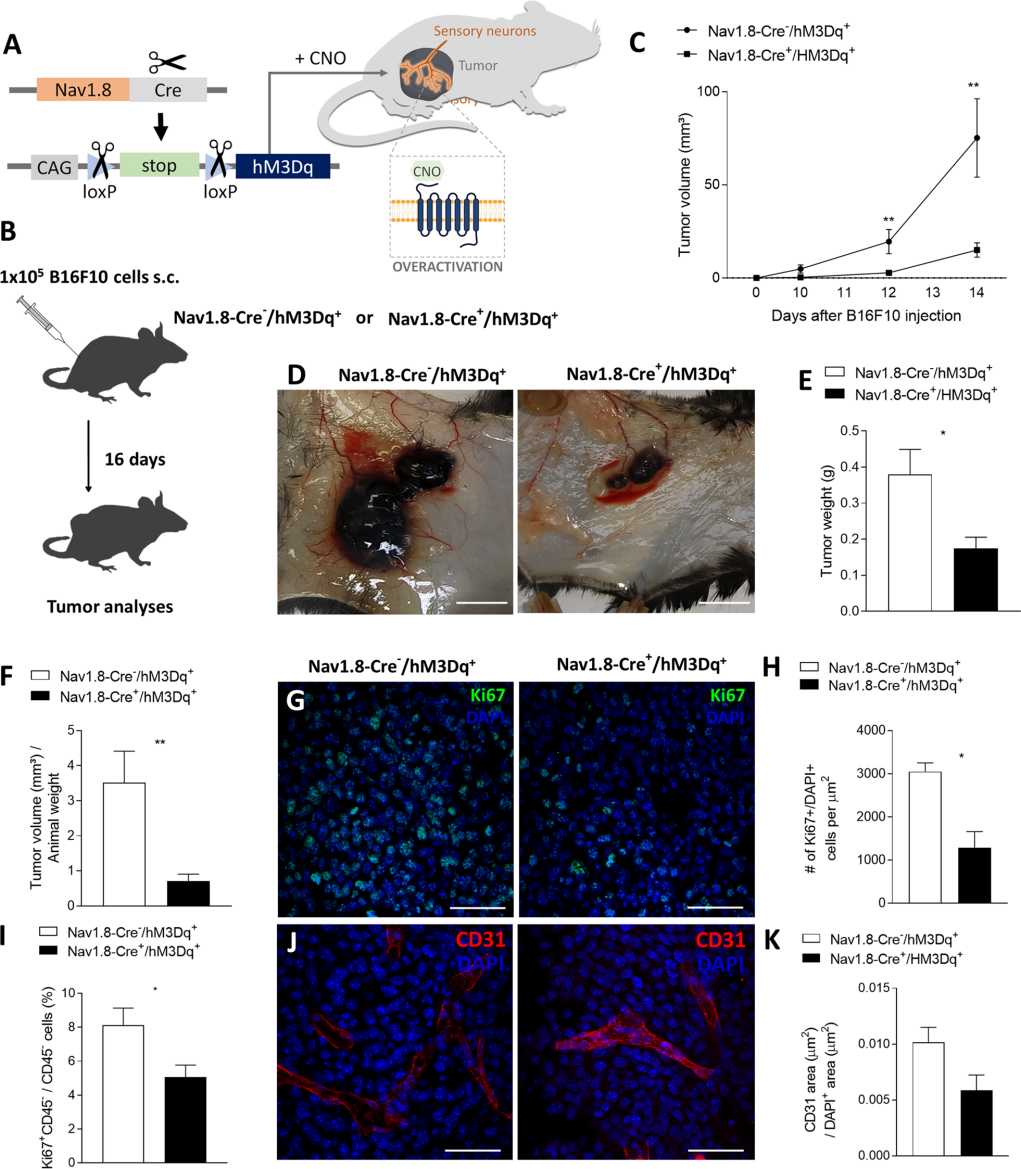
Overstimulation of sensory Nav1.8 + nerve fibers decreases melanoma growth. **A** Schematic diagram of the Nav1.8-Cre + /hM3Dq + experimental mouse model. Cre recombinase directs the expression of hM3Dq specifically to sensory neurons in those mice. After the administration of CNO to those mice, neuronal activity in sensory neurons is overactivated. **B** Representation of the protocol for subcutaneous allograft melanoma growth. 1 × 10^5^ B16F10 melanoma cells were subcutaneously injected into Nav1.8‐Cre^−^/hM3Dq + (n = 14) and Nav1.8‐Cre + /hM3Dq + (n = 13) mice, and tumors were removed for analysis after 16 days. CNO was injected daily intra-peritoneally at 1 mg/kg. **C** Development curve of tumor growth from Nav1.8‐Cre^−^/hM3Dq^+^ and Nav1.8‐Cre^+^/hM3Dq^+^. Tumor volumes were assessed over time with a caliper. **D** Representative macroscopic images of B16F10 melanoma tumors after dissection, left panel (Nav1.8‐Cre^−^/hM3Dq^+^) and right panel (Nav1.8‐Cre^+^/hM3Dq^+^). **E** Tumor weight. (Nav1.8‐Cre^−^/hM3Dq^+^: 0.38 ± 0.07; Nav1.8‐Cre^+^/hM3Dq^+^: 0.17 ± 0.03). **F** Tumor weight corrected by animal body weight. **G** Representative immunofluorescence images of tumors labelled for Ki67 (Ki67; green) to identify cell proliferation and nuclei (DAPI; blue). **H** Quantification of proliferation in melanomas from Nav1.8‐Cre^−^/hM3Dq^+^ and Nav1.8‐Cre^+^/hM3Dq^+^ animals. **I** Quantification of proliferation (Ki67 +) by flow cytometry in CD45- cells from tumors of Nav1.8‐Cre^−^/hM3Dq^+^ and Nav1.8‐Cre^+^/hM3Dq^+^ mice. **J** Representative immunofluorescence images of tumor sections labelled for endothelial cells (CD31; red) to identify blood vessels and nuclei (DAPI; blue). **K** Quantification of angiogenesis in melanomas by blood vessel area. Data are shown as mean ± SEM. Unpaired t test (ns *P* > 0.05; *P < 0.05; **P < 0.01)

As we found that inhibition of sensory neuron activity promotes melanoma advancement, we hypothesized that increasing sensory excitability would result in the reverse: blockage of melanoma progression. To test this hypothesis, we used again chemogenetics, by which we induced the expression of excitatory hM3Dq DREADDs [127] only in Nav1.8 + sensory neurons. We crossed Nav1.8-Cre mice to a mouse line with a Cre-dependent evolved Gq protein-coupled receptor (hM3Dq) expression. In the resulting Nav1.8-Cre + /hM3Dq + mice, upon removal of loxP-stop-loxP cassette by Cre recombination, the Gq-coupled hM3Dq is expressed specifically in Nav1.8-sensory nerve fibers. Sensory neurons in those mice can thus be overactivated by the administration of CNO. It has been shown previously that Gq-DREADD activation by CNO increases neuronal activity in the targeted neurons, including sensory neurons [68, 90], without changing the number of neurons [110]. To evaluate the role of sensory stimulation on tumor growth, we transplanted subcutaneously B16F10 melanoma cells to the lower right flank of both stimulatory DREADD-expressing mice (Nav1.8-Cre + /hM3Dq +) and their controls (Nav1.8-Cre-/hM3Dq +). Following the cancer cell implantation, we treated mice daily with CNO to induce Nav1.8 + sensory neuron activation (controls were also treated with CNO) (Fig. 3A, B). After repeated sensory neuron activation, melanoma development was decreased in the sensory neuron-overactivated mice when compared to the controls (at day 14, tumor volume per body weight was reduced from 3.51 ± 0.89 to 0.71 ± 0.20 mm^3^; at day 16, tumor weight was reduced from 0.38 ± 0.07 to 0.17 ± 0.03 g; Fig. 3C–F). Animal weights were not affected by genetic stimulation of sensory neurons in melanoma‐bearing mice (data not shown). Moreover, genetic overactivation of sensory neurons led to a decrease in proliferating cells within the tumor (from 3050 ± 203 to 1292 ± 367 Ki67 + cells per μm^2^, analyzed by immunohistochemistry) (Fig. 3G, H), corroborated by flow cytometry analysis of CD45- cells for Ki67 expression (the was a decrease from 8.13 ± 1.00 to 5.07 ± 0.70% of CD45-/Ki67 + cells within the population of CD45- cells) (Fig. 3I). Additionally, there was a decrease in the intra‐tumoral blood vessels’ area (from 0.010 ± 0.001 to 0.006 ± 0.001 µm^2^ of CD31 + area / µm^2^ of tumor area) (Fig. 3J, K). Our data suggest that increase in neuronal activity in sensory neurons counteracts melanoma development.

### Increase in sensory neuron activty affects melanoma immunosurveillance

Functional studies in combination with histological analysis have demonstrated that tumor-infiltrating immune cells modulate melanoma cells’ behavior, altering cancer outcomes [38, 72, 79, 83, 112, 130, 133, 134, 152, 153]. Given that sensory neurons may influence immune responses in non-cancer contexts, we sought to probe whether sensory neurons stimulation alters immune surveillance within the tumor.

**Fig. 4:**
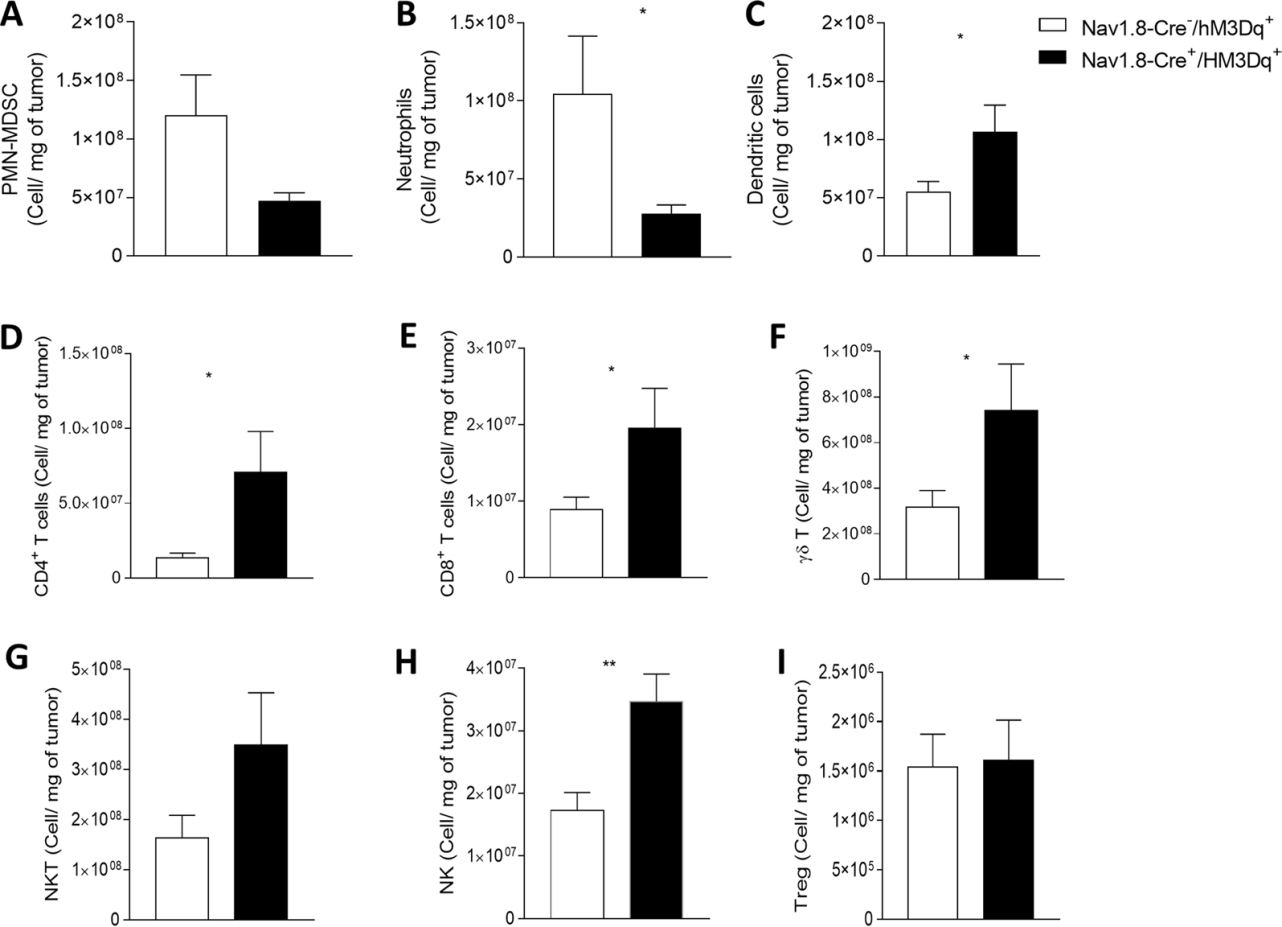
Sensory neurons overactivation improves anti-tumor immunity by decreasing tumor-infiltrating immunosuppressive cells, increasing dendritic cells and by promoting CD4 + T, CD8 + T, γδT, NKT, and NK-cell infiltration. Immune cells from B16F10–inoculated mice were analyzed ex vivo in Nav1.8-Cre^−^/hM3Dq^+^ (n = 14) and Nav1.8-Cre^+^/hM3Dq^+^ (n = 13) mice. Column charts show the proportion of PMN/MDSC (**A**) Neutrophils (**B**) and Dendritic cells (**C**) quantified in the tumor microenvironment. (D-I) TIL from B16F10–inoculated Nav1.8-Cre-/hM3Dq + (n = 14) and Nav1.8-Cre + /hM3Dq + (n = 13) mice were analyzed ex vivo. Absolute number of CD4 + T cells (**D**), CD8 + T cells (**E**), γδ T cells (**F**), NKT cells (**G**), NK cells (**H**), and Treg cells (**I**) from the melanomas of B16F10–inoculated mice. Data are shown as mean ± SEM, Unpaired t test, *.01 < *P* < .05; **.001 < *P* < .01

Accumulating evidence has demonstrated that tumor-infiltrating neutrophils and PMN-MDSCs promote tumor development and progression [21, 39, 65, 107, 136, 138, 140, 150]. Thus, we evaluated whether these cells are affected by sensory neurons’ overactivation. We found that the number of melanoma-infiltrating neutrophils and PMN-MDSCs was significantly decreased in the sensory neuron-overactivated mice (Nav1.8-Cre + /hM3Dq +) when compared to the controls (Nav1.8-Cre-/hM3Dq +) (from 12.02 × 10^7^ ± 3.45 × 10^7^ to 4.69 × 10^7^ ± 7.10 × 10^6^ PMN-MDSCs per mg of tumor; and from 10.45 × 10^7^ ± 3.70 × 10^7^ to 2.78 × 10^7^ ± 5.65 × 10^6^ neutrophils per mg of tumor) (Fig. 4A, B). On the other hand, we found that the number of tumor-infiltrating dendritic cells, which counteract the proliferation of melanoma cells [137], was significantly increased (from 5.53 × 10^7^ ± 8.80 × 10^6^ to 1.07 × 10^8^ ± 2.27 × 10^7^ dendritic cells per mg of tumor) (Fig. 4C).

**Fig. 5:**
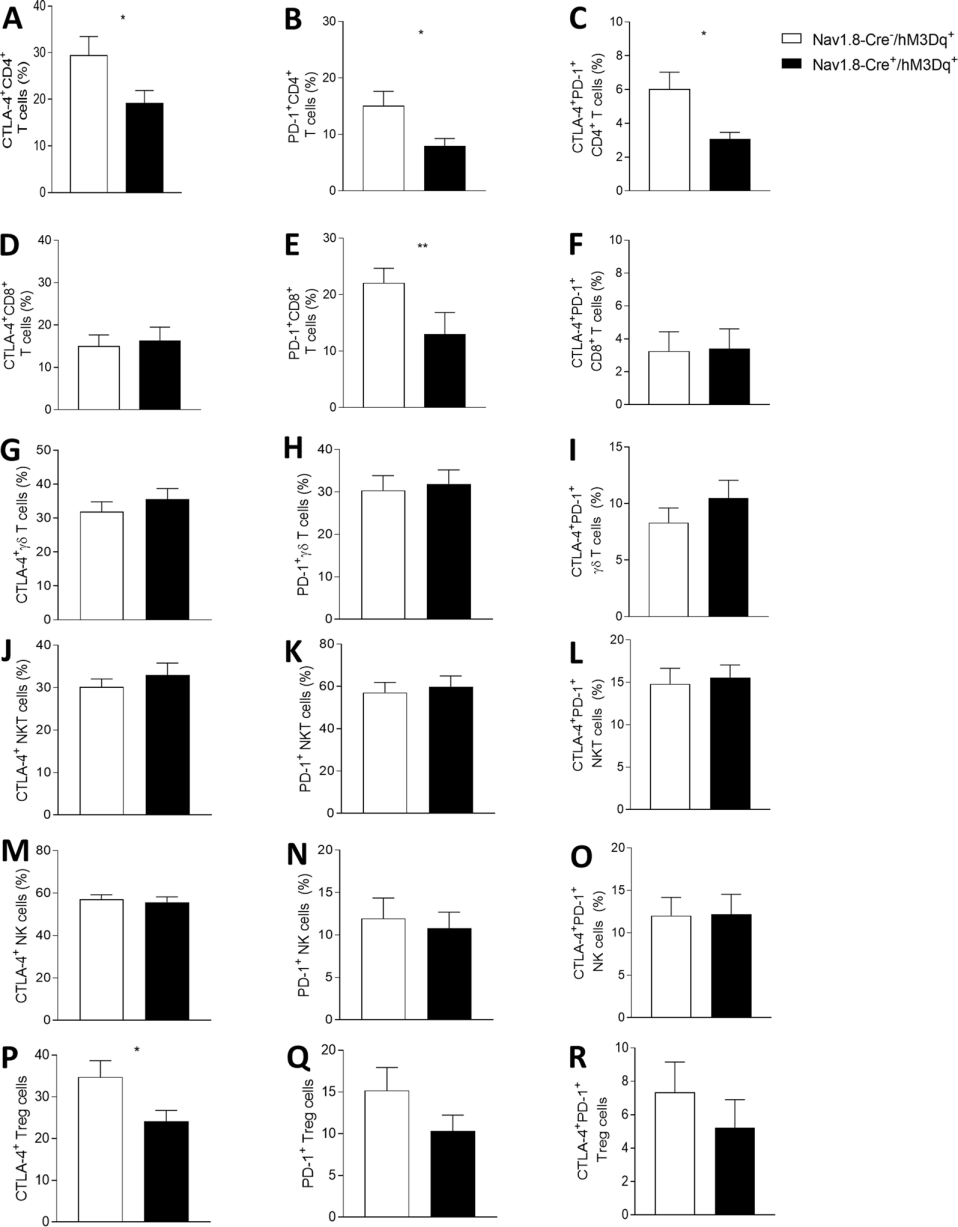
Sensory neurons overstimulation prevent the increase of immune checkpoint markers in tumor infiltrating CD8 + T cells and CD4 + T cells. Immune cells from tumors of B16F10–inoculated mice were analyzed ex vivo in Nav1.8-Cre^−^/hM3Dq^+^ (n = 14) and Nav1.8-Cre^+^/hM3Dq^+^ (n = 13) mice. Column charts show proportion of CTLA-4 (**A**, **D**, **G**, **J**, **M**, **P**), PD-1 (**B**, **E**, **H**, **K**, **N**, **Q**) and CTLA-4/PD-1 co-expressing (**C**, **F**, **I**, **L**, **O**, **R**) CD4 + T cells (**A**, **B**, **C**), CD8 + T cells (**D**, **E**, **F**), γδ T cells (**G**, **H**, **I**), NKT cells (**J**, **K**, **L**), NK cells (**M**, **N**, **O**), and Treg cells (P, Q, R) from tumors of B16F10–inoculated mice. Data are shown as mean ± SEM, Unpaired t test, *.01 < *P* < .05; **.001 < *P* < .01

Recent breakthroughs in cancer immunotherapy have revealed the remarkable ability of the immune system to fight different types of cancers, including melanoma. The phenotypes and numbers of prevalent tumor-infiltrating lymphocytes are predictive of response to immunotherapy and key modulators of disease progression. Thus, we examined how tumor-infiltrating lymphocytes are affected by sensory neurons’ overstimulation. We detected an increase in tumor-infiltrating CD4 + T cells (from 2.91 × 10^6^ ± 1.04 × 10^6^ to 1.09 × 10^7^ ± 2.92 × 10^6^ cells per mg of tumor), CD8 + T cells (from 8.94 × 10^6^ ± 1.60 × 10^6^ to 1.96 × 10^7^ ± 5.20 × 10^6^ cells per mg of tumor), γδ T cells (from 31.76 × 10^7^ ± 7.32 × 10^7^ to 74.14 × 10^7^ ± 20.40 × 10^7^ cells per mg of tumor), NKT cells (from 16.34 × 10^7^ ± 4.6 × 10^7^ to 34.92 × 10^7^ ± 10.42 × 10^7^ cells per mg of tumor) and NK cells (from 1.72 × 10^7^ ± 2.90 × 10^6^ to 3.47 × 10^7^ ± 4.40 × 10^6^ cells per mg of tumor) (Fig. 4D–H), while regulatory T cells, which mediate immunosuppression in the tumor microenvironment [66], were not altered (Fig. 4I). Immune checkpoint molecules, such as cytotoxic T lymphocyte antigen 4 (CTLA-4) and programmed cell death 1 (PD-1), act fine-tuning the intense immune responses that might kill healthy cells [27, 55, 122]. Their expression in cytotoxic T cells may lead to dysfunction of these cells, affecting their effector function [11, 146]. We found that increase in the firing of sensory neurons prevented the increase of immune checkpoint markers of tumor infiltrating CD8 + T cells and CD4 + T cells (Fig. 5 and Additional file 1: Figure 1). The percentage of CTLA-4-expressing CD4 + tumor-infiltrating lymphocytes decreased from 29.43 ± 4.04% in Nav1.8-Cre^−^/hM3Dq^+^ to 19.08 ± 2.80% in Nav1.8-Cre^+^/hM3Dq^+^ animals (Fig. 5A); similarly, the percentage of PD-1-expressing CD4 + tumor-infiltrating lymphocytes decreased from 15.02 ± 2.62% in Nav1.8-Cre^−^/hM3Dq^+^ to 7.85 ± 1.43% in Nav1.8-Cre^+^/hM3Dq^+^ mice (Fig. 5B, C). The percentage of PD-1-expressing CD8 + tumor-infiltrating cytotoxic lymphocytes also decreased from 22,03 ± 2,66% in Nav1.8-Cre^−^/hM3Dq^+^ to 12.99 ± 3.85% in Nav1.8-Cre^+^/hM3Dq^+^ animals (Fig. 5E), while the expression of CTLA-4 did not vary in these cells (Fig. 5D, F). In addition, no differences were found in CTLA-4 and PD-1 expression on γδ T cells (Fig. 5G, H and I), NKT cells (Fig. 5J, K and L), NK cells (Fig. 5M, N and O) and Treg cells (Fig. 5P, Q and R). Overall, our data suggest that sensory neurons overactivation induces improvement of T cells effector functions within the tumor microenvironment.

**Fig. 6:**
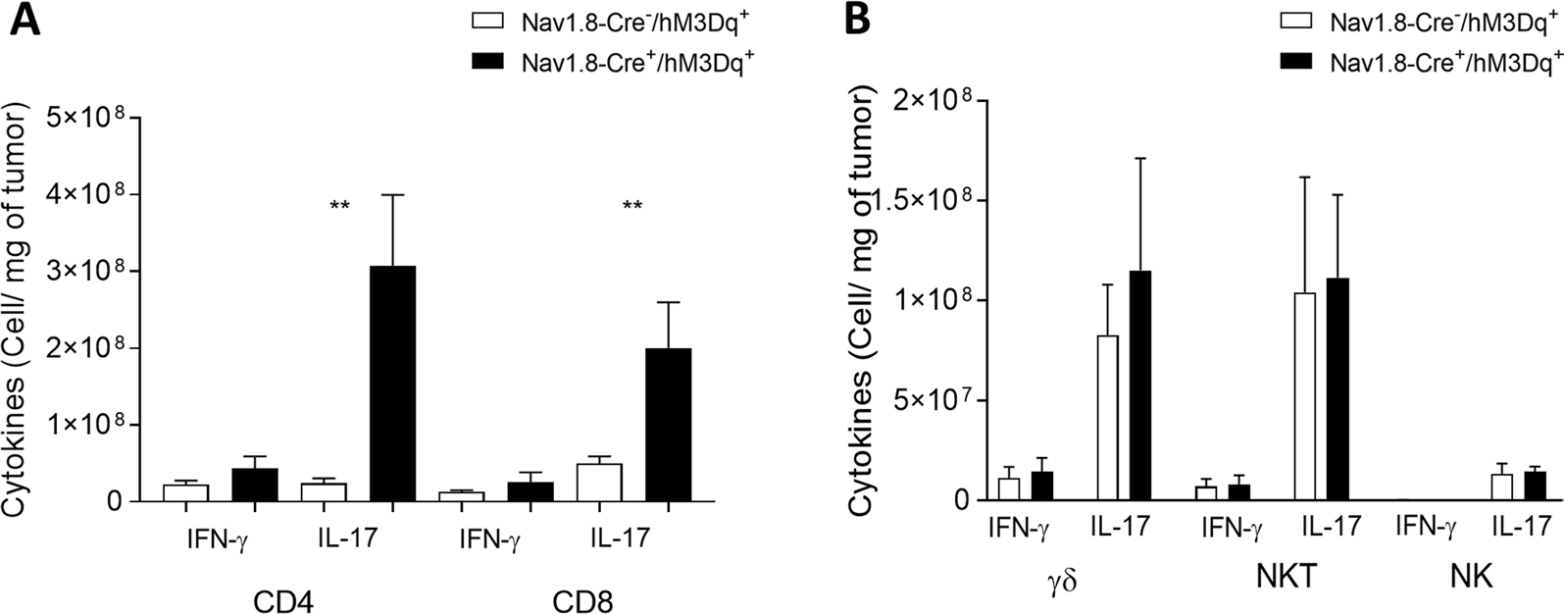
Sensory neurons overactivation promote an increase in tumor-infiltrating IL-17-producing CD4 + and CD8 + T cells. TIL from melanomas of B16F10–inoculated Nav1.8-Cre-/hM3Dq + (n = 14) and Nav1.8-Cre + /hM3Dq + (n = 13) mice were analyzed. TIL from B16F10–inoculated mice were analyzed after 4 h of culture. **A** Column charts show absolute numbers of CD4 + and CD8 + T cells producers of IFN-γ and IL-17. **B** Column charts show absolute number of γδ T cells, NKT cells and NK cells producing IFN-γ and IL-17. Cytokines levels were measured in cells isolated from tumors of B16F10–inoculated Nav1.8-Cre^−^/hM3Dq^+^ and Nav1.8-Cre^+^/hM3Dq^+^ mice. Data are shown as mean ± SEM, Unpaired t test, *.01 < *P* < .05; **.001 < *P* < .01

It has been reported that CD4 + and CD8 + lymphocytes secreting IL-17 promote melanoma regression [91, 99]. Here, we detected in response to sensory neuron firing an increase in melanoma-infiltrating IL-17-producing CD4 + T cells (from 2.45 × 10^7^ ± 6.05 × 10^6^ to 30.78 × 10^7^ ± 9.20 × 10^7^ cells per mg of tumor) as well as in melanoma-infiltrating IL-17-producing CD8 + T cells (from 5.02 × 10^7^ ± 0.90 × 10^7^ to 20.08 × 10^7^ ± 5.92 × 10^7^ cells per mg of tumor) (Fig. 6A). In parallel, we did not detect significant changes in the number of other tumor-infiltrating lymphocytes producing IL-17 or in IFN-γ-producing lymphocytes after sensory neuron overactivation (Fig. 6B). Altogether, our results suggest that sensory neurons induce a Th17-immune response in the melanoma microenvironment.

**Fig. 7:**
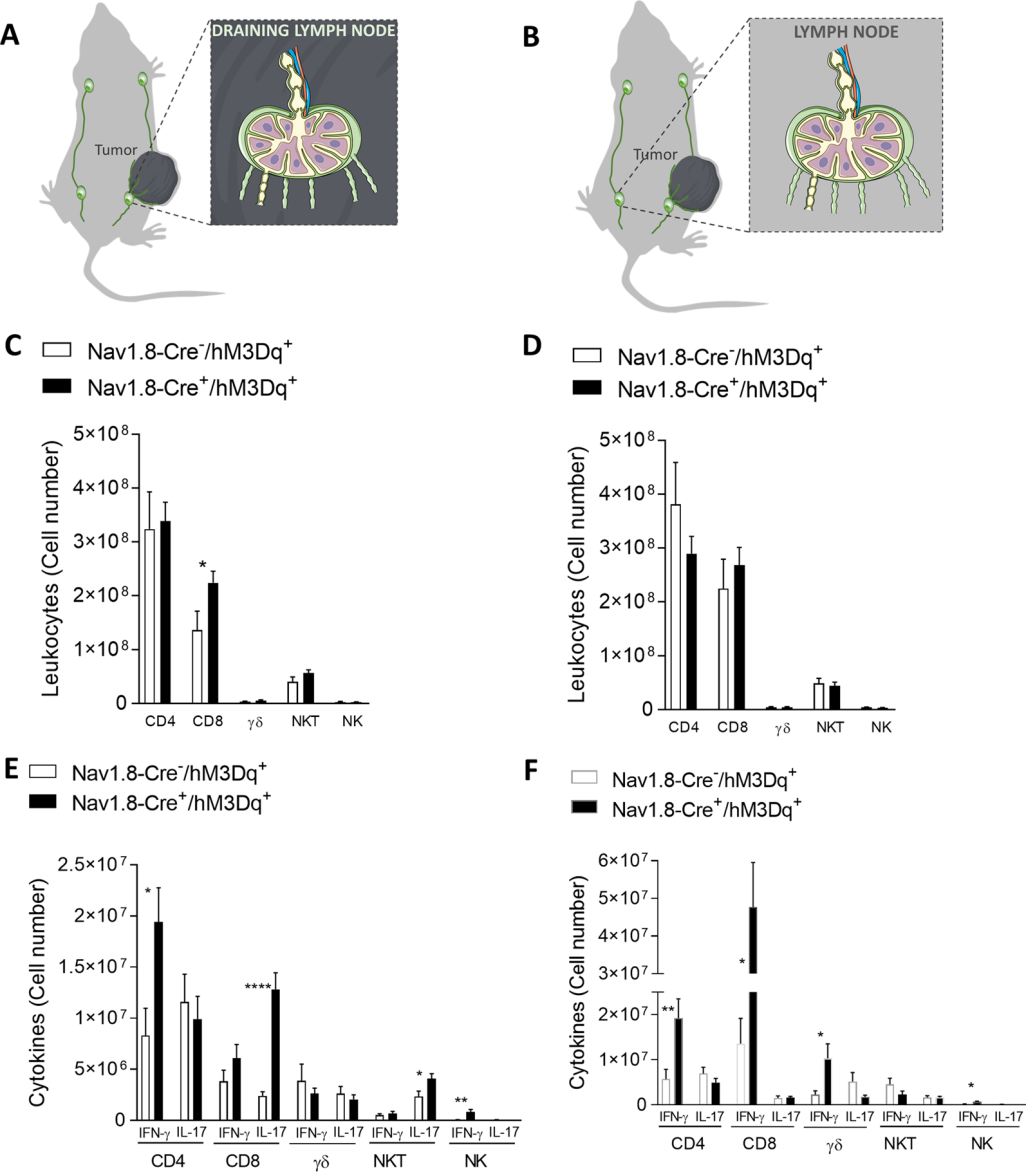
Tumor-draining lymph nodes present an increase in effector CD8 + T-cells after overstimulation of sensory neurons, while the number of lymphocytes in tumor non-draining lymph nodes doesnt change. **A** Schematic representation of the collected tumor draining lymph nodes. TIL from tumor-draining lymph nodes of B16F10–inoculated Nav1.8-Cre-/hM3Dq + (n = 14) and Nav1.8-Cre + /hM3Dq + (n = 13) mice were analyzed. **B** Absolute number of CD4 + , CD8 + , γδ, NKT and NK cells from tumor-draining lymph nodes of B16F10–inoculated mice. **C** IFN-γ and IL-17 were quantified in CD4, CD8, γδ, NKT and NK cells. **D** Schematic representation of the collected tumor non-draining lymph nodes. TIL from tumor non-draining lymph nodes of B16F10–inoculated Nav1.8-Cre-/hM3Dq + (n = 14) and Nav1.8-Cre + /hM3Dq + (n = 13) mice were analyzed. **E** Absolute number of CD4 + , CD8 + , γδ, NKT and NK cells from tumor non-draining lymph nodes of B16F10–inoculated mice. **F** IFN-γ and IL-17 were quantified in CD4, CD8, γδ, NKT and NK cells. Cytokines levels were measured in cells from tumor-draining and tumor non-draining lymph nodes of B16F10–inoculated Nav1.8-Cre-/hM3Dq + and Nav1.8-Cre + /hM3Dq + mice. Data are shown as mean ± SEM. Unpaired t test, *.01 < *P* < .05; **.001 < *P* < .01; ****P < 0.0001

Lymph nodes are an integral part of the adaptive immune system in our organism and are essential for the effective immune responses. Melanoma draining lymph nodes are influenced by the primary tumor, but may also prime the immune suppressive microenvironment, playing critical roles in promoting cancer immune escape [33, 86, 87, 97]. It is completely unknown whether sensory neuron overactivation may affect the immune cells also within the tumor draining lymph nodes. Herein, we analyzed immune cells from tumor draining and non-tumor-draining lymph nodes from CNO-treated stimulatory DREADD-expressing animals (Nav1.8-Cre + /hM3Dq +) and their controls (Nav1.8-Cre-/hM3Dq +). Tumor draining and non-tumor-draining lymph nodes were isolated from the ipsilateral and contralateral side, respectively, of the implanted melanoma (Fig. 7A, B). We found that the effect of sensory neurons stimulation in tumor draining lymph nodes mimics the immune response within the primary tumor, but not in non-tumor-draining lymph nodes. In the tumor-draining lymph nodes, we found an increase in the number of CD8 + cytotoxic T cells after sensory neurons’ overstimulation (from 13.68 × 10^7^ ± 3.50 × 10^7^ to 22.41 × 10^7^ ± 2.17 × 10^7^ cells per mg of tumor) (Fig. 7C); while we did not detect any differences in the numbers of T cells in the tumor non-draining lymph nodes (Fig. 7D). These data indicate a possible priming effect of tumor on the adjacent draining lymph nodes.

We also observed, in the tumor-draining lymph nodes, increases in IFN-γ-producing CD4 + T cells (from 8.31 × 10^6^ ± 2.65 × 10^6^ to 1.94 × 10^7^ ± 3.36 × 10^6^ cells per lymph node), in IFN-γ-producing NK cells (from 4.72 × 10^4^ ± 2.46 × 10^4^ to 8.44 × 10^5^ ± 2.22 × 10^5^ cells per lymph node), in IL-17-producing CD8 + T cells (from 2.38 × 10^6^ ± 4.27 × 10^5^ to 1.28 × 10^7^ ± 1.64 × 10^6^ cells per lymph node), and in IL-17-producing NKT cells (from 2.35 × 10^6^ ± 5.19 × 10^5^ to 4.08 × 10^6^ ± 5.00 × 10^5^ cells per lymph node) after sensory neurons CNO-stimulation (Fig. 7E). In tumor non-draining lymph nodes, we detected increases in IFN-γ-producing CD4 + T cells (from 5.73 × 10^6^ ± 2.17 × 10^6^ to 1.92 × 10^7^ ± 4.23 × 10^6^ cells per lymph node), in IFN-γ-producing CD8 + T cells (from 1.36 × 10^7^ ± 5.57 × 10^6^ to 4.77 × 10^7^ ± 1.19 × 10^7^ cells per lymph node), in IFN-γ-producing γδ T cells (from 2.27 × 10^6^ ± 8.55 × 10^5^ to 1.03 × 10^7^ ± 3.22 × 10^6^ cells per lymph node), and in IFN-γ-producing NK cells (from 1.80 × 10^5^ ± 4.70 × 10^4^ to 6.82 × 10^5^ ± 1.53 × 10^5^ cells per lymph node) (Fig. 7F). Our data suggest that lymphocytes at the lymph nodes may be contributing to the response against the melanoma observed within the tumor microenvironment after sensory neurons’ overactivation as both, IFN-γ and IL-17, may contribute to enhance the anti-tumoral response in the context of melanoma [92, 109, 151]. Altogether, our data suggest that sensory neurons stimulation alters immune surveillance that impacts melanoma development.

### High expression of genes related to sensory neurons correlates with best prognosis in human melanoma patients

**Fig. 8:**
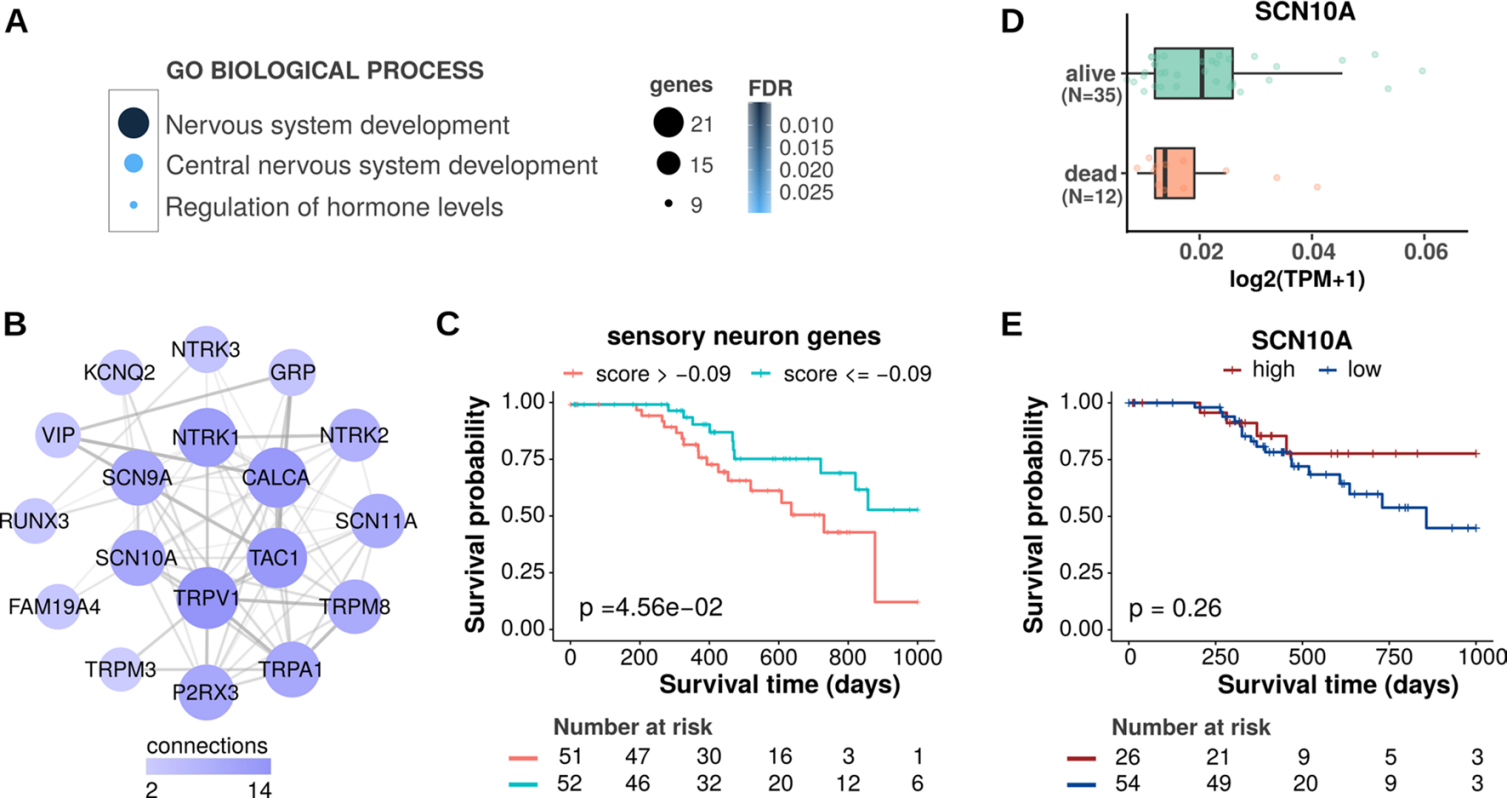
Overexpression of genes related to sensory nerves is associated with Skin Cutaneous Melanomas (SKCM) patients improved survival. **A** Biological Processes of genes overexpressed in Skin Cutaneous Melanomas (SKCM) samples from alive patients versus dead patients. Patients were stratified (alive or dead) based on their vital status in a 5-year interval of their tumor diagnosis (clinical data available at TCGA and curated by Liu et al. (2018) [85]. We stratified patients in two groups, alive or dead, based on a 5-year interval of their tumor diagnosis. **B** Interactions among genes related to sensory neurons. **C** Gene signature using sensory neurons-related genes. High expression of these genes (lower patient scores) is associated with a better overall survival of SKCM patients (patients were stratified based on their median Reboot score). Overall survival of patients showing expression of sensory neurons-related genes. More negative scores are associated with higher gene expression. **D** Expression of SNC10A (Nav1.8) in SKCM samples. Only patients presenting tumors expressing SNC10A were used. **E** We evaluated the survival probability of patients with melanoma based on their tumor transcriptome. Patients were stratified based on lower/upper quartiles of SCN10A expression values. Overall survival of SKCM patients based on the level of expression of SCN10A. High expression of SCN10A (Nav1.8) correlates with best outcomes in patients with melanoma

In order to investigate our findings also in human tumors, we analyzed The Cancer Genome Atlas (TCGA) samples from 103 Skin Cutaneous Melanoma (SKCM) patients. First, we stratified SKCM patients in two groups, alive or dead based on a 5-year interval. Next, we searched for differentially expressed genes between these groups. We found 112 up-regulated and 195 down-regulated genes (|log2(Fold change)|≥ 1 and adjusted *P*-value < 0.05; Table 2). Next, we performed a Gene Ontology (GO) analysis of Biological Processes (BP) in which these genes are involved. The up-regulated genes set are enriched for three biological processes, while the down-regulated genes are enriched to a wide variety of processes (Fig. 8A; Table 3). Curiously, for the former (up-regulated genes), two out of three Biological Processes represent nervous system development (Fig. 8A), indicating the importance of neuronal networks in melanoma outcomes. Next, we investigated the interactions (and putative regulation) of 34 sensory neurons-related genes which were selected from the literature [29, 37, 51, 114, 141, 144] (Table 4). Figure 8B shows a strong connection among 18 of these genes, suggesting that they work on the same cellular pathways or cell types. Next, using the expression levels of these 34 gene markers for sensory neurons we investigated their potential to be "a signature" associated with SKCM cancer patient survival. Figure 8C shows that high expression of these genes (lower patient scores) are associated with a better overall survival of SKCM patients. Next, we investigated the expression of these genes in the two sample sets (alive and dead patients). We found that SCN10A, which codifies Nav1.8, a key gene based on which our mouse models target sensory neurons, is more expressed in alive than in dead patients (Fig. 8D). Finally, we investigated the impact of SCN10A expression on SKCM patients' survival. Figure 8E shows that high expression of SCN10A trends to be associated with a better overall survival of SKCM patients, even without statistical support (*P*-value = 0.26). Taken together, these results confirm that genes related to nervous system development are enriched in samples from live Skin Cutaneous Melanoma patients. Focusing on gene markers for sensory neurons, we confirmed that these genes are strongly connected, suggesting a synergistic activity, and that the higher expression of some of these genes are associated with a higher overall survival. Strikingly, the high expression of SCN10A is potentially associated with better SKCM patient survival, indicating that the presence of sensory neurons within melanoma counteracts cancer progression. We also found that TCGA samples from tumors with a worst prognosis (dead patients) have an enrichment of genes promoting angiogenesis (Tables 3 and 5; 15 genes related to angiogenesis). We focused on this set of 15 genes related to angiogenesis and we confirmed that they are strongly connected (Additional file 2: Fig. 2), indicating a synergic function in promoting angiogenesis. Our results indicate that there is an increase in genes related with angiogenesis in tumors with worst prognosis (from dead patients) and a decrease in their expression in tumors from alive patients which show an increased expression of SCN10a, a sensory neuron marker used in this study (Fig. 8C, D, E). By using the CIBERSORT tool [102], we investigated immune infiltrated cells in the same TCGA cohort of alive vs. dead patients (Additional file 3: Fig. 3). CIBERSORT uses gene expression (RNA sequencing data) and support vector regression combined with prior knowledge of expression profiles from purified leukocyte subsets (gene signatures) to produce an estimation of the abundances of immune infiltrated cells subpopulations in a sample. In line with our data presented in this manuscript, we have checked the enrichment of immune infiltrated cells in the tumors of patients alive vs. dead (Additional file 4: Fig. 4). We found an increase of CD4 + T cells, CD8 + T cells, NK cells and dendritic cells in patients with better prognosis (alive). Thus, tumors showing a better prognosis (alive) have an increased infiltration of some key immune cells. Additionally, microarray data evidenced a down-regulation of genes related to the Th17 immune response in melanoma patients (Additional file 5: Fig. 5). These analyses are consistent with the data generated in our mouse models: that the overactivation of sensory innervations in the tumor microenvironment was associated with suppressed melanoma progression. Albeit gene expression in tumor biopsies from human cancer patients is used as a tool to define novel biomarkers and  to contribute to prognosis, the obtained data should be also validated by the quantification of sensory neuron-related proteins in human melanoma biopsies and correlation with clinical outcomes in future research.

**Table 2:** Analyzes of genes from The Cancer Genome Atlas (TCGA) samples from 103 Skin Cutaneous Melanoma (SKCM) patients

**Up-regulated genes in alive × dead**		
Gene.symbol	log2FC	FDR
SLC5A4	4.35910	1.29E−09
VGF	3.26059	1.06E−05
NPPC	3.24826	2.60E−05
LINC00698	3.76414	3.90E−05
SPACA3	2.67598	1.97E−04
VCX3A	4.95714	2.21E−04
PRSS56	4.13411	4.15E−04
ARHGAP8	2.75929	4.15E−04
VCX	3.63292	4.28E−04
LINC01287	3.48781	7.19E−04
NGFR	1.99998	1.16E−03
NAT16	2.23940	1.16E−03
RP13-143G15.4	2.26728	1.34E−03
HLA-J	1.67323	1.38E−03
LHFPL4	2.80159	2.04E−03
RP11-376N17.4	2.27953	2.10E−03
ZNF689	1.08305	2.33E−03
DCD	6.91950	2.44E−03
SLITRK5	2.38821	2.59E−03
ARPP21	3.84108	2.67E−03
TFAP2B	2.73943	2.68E−03
VIT	2.42509	3.21E−03
HPCAL4	1.70772	3.30E−03
LINC00645	3.54408	4.05E−03
KLHL32	1.29974	4.09E−03
TRIML2	2.32595	4.80E−03
GFAP	2.04141	5.21E−03
MYOZ2	2.33172	6.94E−03
PPY	2.13167	7.88E−03
ARX	2.02055	8.39E−03
LRRTM2	1.75474	8.39E−03
C20orf203	1.95612	8.43E−03
LSMEM2	1.36377	8.43E−03
NRTN	1.74226	8.58E−03
RP11-809C18.3	1.54681	8.89E−03
FOSB	1.08702	9.10E−03
PASD1	4.57206	9.14E−03
UNC93B3	1.84987	9.14E−03
RP11-469H8.6	2.35144	9.14E−03
BCO1	2.17260	9.80E−03
XKR7	1.93913	1.00E−02
RDH5	1.36688	1.04E−02
PAGE1	3.69526	1.10E−02
RP5-907D15.4	1.73346	1.10E−02
AC003092.1	2.31155	1.20E−02
IGHV1-58	3.09550	1.20E−02
FOXG1	3.56195	1.36E−02
GBA3	2.67229	1.39E−02
FKSG51	1.89383	1.39E−02
BTNL8	1.87248	1.46E−02
ACHE	1.18749	1.52E−02
KCNJ11	1.81031	1.52E−02
ENPP7P2	1.45231	1.64E−02
CCKBR	2.06353	1.73E−02
PCSK1N	1.87835	1.76E−02
RP11-114G11.5	3.04186	1.76E−02
EFTUD1P1	2.13819	1.77E−02
OR2N1P	5.08848	1.83E−02
CA10	2.21148	1.85E−02
SLCO5A1	1.55420	1.88E−02
IBSP	1.81676	1.91E−02
RP11-88I21.1	3.56590	1.94E−02
MAGEA9	5.10603	1.96E−02
SYP	1.03514	2.15E−02
NBEAP1	1.55393	2.17E−02
RP5-965G21.4	1.14894	2.18E−02
MPZ	1.95997	2.22E−02
RP11-299H22.3	2.95030	2.22E−02
FBXO2	1.34819	2.31E−02
LGSN	2.41190	2.31E−02
RDH8	2.14341	2.32E−02
AC073325.2	1.90901	2.38E−02
GAGE1	3.92238	2.44E−02
MYB	1.16344	2.55E−02
AATK	1.23997	2.63E−02
DOK7	1.48390	2.72E−02
AC068580.7	2.45353	2.72E−02
RP11-36D19.9	1.99056	2.72E−02
HAPLN2	1.51979	2.73E−02
TDRD12	2.24362	3.09E−02
RP11-159H10.3	1.83751	3.18E−02
CHGB	1.62908	3.32E−02
RCN3	1.25033	3.32E−02
RP5-1171I10.5	1.33948	3.32E−02
NMRK2	2.15724	3.33E−02
TNNI3	1.50305	3.33E−02
DPEP3	1.81124	3.33E−02
DPYSL5	2.12990	3.33E−02
ZNF365	1.53513	3.39E−02
KCNQ2	1.98246	3.51E−02
PMP2	2.27248	3.53E−02
HAVCR1	2.17185	3.56E−02
RP1-140K8.5	1.72113	3.72E−02
ROR1-AS1	2.03339	3.75E−02
C1QTNF1-AS1	1.82857	3.75E−02
WFDC1	1.74139	3.99E−02
DEFB126	2.40171	3.99E−02
LBP	1.71514	3.99E−02
CDH12	2.39970	3.99E−02
FABP7	2.11963	3.99E−02
MAGEB17	1.73309	3.99E−02
MYBPC1	1.99301	3.99E−02
RP4-764D2.1	1.03824	4.07E−02
CD5L	1.77129	4.21E−02
CPN2	1.81842	4.21E−02
ZNF727	2.20430	4.21E−02
CST1	2.16587	4.35E−02
RP11-9G1.3	2.55148	4.48E−02
LL22NC03-22D1.1	2.49624	4.50E−02
NPFFR1	1.60868	4.54E−02
MYRIP	1.45769	4.67E−02
RP11-369C8.1	2.61906	4.95E−02

**Down-regulated genes in alive × dead**		
Gene.symbol	log2FC	FDR
AVPR1A	− 2.06066	8.65E−08
KRT16P2	− 5.08308	1.00E−07
CHST8	− 3.38063	7.38E−07
ST6GAL2	− 2.68516	1.74E−06
PRSS35	− 3.35395	4.71E−6
SLC8A3	− 3.18840	9.15E−06
CRABP1	− 3.07142	9.15E−6
HSPB3	− 3.24770	2.15E−05
TREX2	− 2.43750	2.15E−05
B3GNT4	− 1.77784	2.26E−05
NPTX1	− 2.61603	1.97E−04
PI3	− 3.60121	2.70E−04
HEYL	-1.67534	2.99E−04
ID3	− 1.32736	3.67E−04
ADRB2	− 1.81924	3.91E−04
C6orf223	− 2.13191	4.15E−04
AJAP1	− 1.92423	4.56E−04
CHRNA4	− 2.95110	4.69E−04
RHCG	− 2.92093	5.10E−04
PART1	− 2.14901	5.50E−04
ALOX12	− 1.23574	5.50E−04
RSPO3	− 1.48997	6.13E−04
GDPD3	− 1.74062	7.38E−04
CNFN	− 2.84236	8.27E−04
ANO2	− 1.70534	9.14E−04
OVOL1	− 2.75293	9.14E−04
CLDN4	− 2.52180	9.56E−04
GREB1L	− 2.35313	1.12E−03
IGLV9-49	− 3.84182	1.20E−03
TGM1	− 2.85878	1.28E−03
CYSRT1	− 2.10472	1.34E−03
LYPD5	− 2.27815	1.34E−03
CYP19A1	− 1.84923	1.41E−03
ACTG2	− 1.63424	1.41E−03
ABCG4	− 2.12582	1.41E−03
FAM3D	− 2.12972	1.41E−03
PAPSS2	− 1.50601	1.41E−03
AC124789.1	− 2.07689	1.41E−03
RASL11B	− 1.54784	1.43E−03
MAFB	− 1.28214	1.43E−03
NGF	− 1.73693	2.01E−03
AC006116.20	− 1.47472	2.01E−03
S100A12	− 2.64402	2.10E−03
KLK14	− 2.29647	2.39E−03
IL1RN	− 2.09008	2.39E−03
B3GNT8	− 1.78028	2.39E−03
GPX3	− 1.88978	2.39E−03
CDA	− 2.22423	2.68E−03
CHN1	− 1.30585	2.68E−03
PRSS27	− 1.71351	2.68E−03
TMEM79	− 1.43989	3.52E−03
NOTCH3	− 1.22297	3.57E−03
FGF18	− 1.35348	3.80E−03
HGF	− 1.19158	4.28E−03
SH3RF3	− 1.23159	4.49E−03
KRT37	− 4.22818	4.57E−03
SH3RF3-AS1	− 1.31293	4.57E−03
ZC3H12A	− 1.35742	4.67E−03
TBX4	− 1.84817	5.39E−03
CLIC3	− 2.31078	5.58E−03
LRRC43	− 1.34142	5.69E−03
NRARP	− 1.42862	5.97E−03
B3GNT3	− 1.97566	6.61E−03
ELF3	− 2.07351	6.73E−03
LRRN2	− 1.90993	6.80E−03
NFE4	− 2.76901	6.80E−03
KLK10	− 2.72909	6.94E−03
LINC01121	− 1.68460	7.15E−03
SDCBP2	− 1.48707	7.88E−03
FAM83G	− 1.50253	7.88E−03
ZNF469	− 1.06394	7.88E−03
ROR2	− 1.15831	7.92E−03
LYNX1	− 1.75074	7.92E−03
VSIG10L	− 1.54237	7.92E−03
PITX1	− 2.50531	8.39E−03
PNMA5	− 2.07397	8.43E−03
LTB4R2	− 1.38009	8.43E−03
GTSF1	− 1.95376	8.89E−03
RP3-449H6.1	− 2.80745	8.89E−03
PKDCC	− 1.47224	9.03E−03
C11orf87	− 1.86276	9.10E−03
C9orf47	− 1.63063	9.10E−03
KLK12	− 3.43996	9.10E−03
SPNS2	− 1.33949	9.43E−03
TCHH	− 1.56077	1.00E−02
ADRA1D	− 1.45232	1.01E−02
LINC00675	− 2.28274	1.01E−02
GLP1R	− 1.93142	1.04E−02
DLX5	− 1.61403	1.05E−02
JUP	− 1.81687	1.10E−02
GREM1	− 1.36182	1.10E−02
FUT2	− 1.65558	1.10E−02
FLJ43879	− 1.73000	1.10E−02
RP4-530I15.9	− 1.37950	1.10E−02
ADAMTSL4	− 1.15965	1.10E−02
LGALS9C	− 1.74166	1.11E−02
RP11-145A3.1	− 1.28593	1.20E−02
LINC00689	− 2.44100	1.20E−02
RP11-57C13.6	− 2.13756	1.20E−02
WNT11	− 1.67218	1.23E−02
SOX11	− 1.31510	1.27E−02
SMCO2	− 1.86262	1.29E−02
AC104654.2	− 1.54674	1.34E−02
RP11-715H19.2	− 2.54830	1.45E−02
MALL	− 1.65870	1.50E−02
SLPI	− 2.56276	1.50E−02
ZNF385B	− 1.82165	1.52E−02
AC079305.10	− 1.07337	1.52E−02
RARRES1	− 1.09119	1.56E−02
C1orf177	− 1.80915	1.64E−02
MIR4635	− 1.81095	1.68E−02
KCNK12	− 1.74033	1.70E−02
CTC-525D6.2	− 3.65998	1.75E−02
FGF7	− 1.14265	1.76E−02
KRT82	− 2.71344	1.76E−02
CTD-2554C21.3	− 2.09957	1.76E−02
VNN3	− 1.87348	1.94E−02
KRT17P6	− 2.54509	1.94E−02
CD36	− 1.47332	1.98E−02
ALDH1L1	− 1.84277	1.99E−02
KRT25	− 3.64328	2.04E−02
GLIS3	− 1.24797	2.06E−02
SPRR2D	− 2.65861	2.06E−02
CEACAM19	− 1.48405	2.11E−02
ZNF154	− 1.19730	2.12E−02
FBLIM1	− 1.00682	2.15E−02
OR7E11P	− 3.14060	2.15E−02
ZNF185	− 1.43741	2.27E−02
MYOC	− 3.14651	2.30E−02
SULT2B1	− 2.16618	2.31E−02
PLA2G4E−AS1	− 1.49669	2.32E−02
IL36G	− 2.54342	2.39E−02
IGKV2-29	− 3.58127	2.43E−02
PADI3	− 1.60173	2.45E−02
WDR87	− 2.67496	2.45E−02
CTD-2555C10.3	− 1.62741	2.45E−02
SMPD3	− 1.51129	2.63E−02
LYPD3	− 2.15151	2.63E−02
SPINK9	− 2.18753	2.63E−02
RP3-405J10.2	− 1.89008	2.63E−02
KRT17	− 2.51044	2.65E−02
RP11-845M18.6	− 2.34005	2.70E−02
CPXM2	− 1.33321	2.72E−02
GDPD2	− 1.69177	2.79E−02
LINC01482	− 1.58713	2.84E−02
KRT8P13	− 2.24864	3.07E−02
ANGPT2	− 1.42767	3.23E−02
TMEM45B	− 1.81149	3.23E−02
KLK13	-2.41601	3.23E−02
IGHE	− 2.55145	3.23E−02
SPRR2A	− 2.94281	3.23E−02
RP11-91J3.3	− 1.87453	3.23E−02
CTC-490G23.2	− 2.34066	3.32E−02
ADAMTS15	− 1.20759	3.33E−02
RHOD	− 1.51767	3.33E−02
COL28A1	− 1.56903	3.33E−02
RP11-752L20.3	− 1.07531	3.33E−02
AC133785.1	− 2.02852	3.47E−02
GNA15	− 1.37100	3.49E−02
FAM46B	− 1.22702	3.53E−02
KRT80	− 2.07094	3.53E−02
TWIST2	− 1.11079	3.53E−02
KRT42P	− 2.45179	3.53E−2
PRSS50	− 1.86776	3.64E−02
TBX1	− 1.26122	3.69E−2
KRT81	− 1.69382	3.75E−02
ALOX12B	− 2.41352	3.75E−02
KCNMA1-AS1	− 1.82562	3.79E−02
HS3ST3A1	− 1.28721	3.84E−02
USP2	− 1.07928	3.99E−02
BMP4	− 1.23615	3.99E−02
G6PC2	− 2.36196	3.99E−02
RP11-169K16.4	− 1.98903	3.99E−02
COL8A1	− 1.22669	4.09E−02
SIX2	− 1.02697	4.09E−02
KRT17P2	− 2.21877	4.21E−02
BDKRB1	− 1.22248	4.35E−02
LINC00857	− 1.30729	4.35E−02
CREB3L1	− 1.04632	4.44E−02
CCBE1	− 1.04094	4.44E−02
PRR15L	− 1.31182	4.48E−02
ST8SIA2	− 1.70296	4.50E−02
S100A9	− 2.02615	4.55E−02
SIK1	− 1.83293	4.60E−02
EPN3	− 1.93269	4.65E−02
ZBTB16	− 1.22227	4.67E−02
ATP8B5P	− 1.00252	4.76E−02
DUOX1	− 1.72011	4.78E−02
TRIM53CP	− 2.79481	4.80E−02
C6orf132	− 1.81165	4.87E−02
MDFI	− 1.36942	4.91E−02
CATSPERB	− 1.85527	4.94E−02
HSPE1P5	− 1.47888	4.94E−02
DEFB4A	− 3.10613	4.95E−02
SDC1	− 1.36628	4.96E−02

**Table 3:** Gene Ontology (GO) and analysis of Biological Processes (BP) in which specific genes are involved

**Up-regulated genes in alive × dead**				
Functional category	Genes in list	Total genes	FDR	Genes
Nervous system development	22	2474	0.004805005	ARX HAPLN2 SLITRK5 VCX3A VCX ZNF365 NRTN TFAP2B NGFR MYB GFAP LRRTM2 LHFPL4 DPYSL5 FABP7 FOXG1 VIT MPZ CA10 KCNQ2 ACHE HPCAL4
Central nervous system development	12	1054	0.029644095	HAPLN2 VCX3A VCX ARX GFAP ZNF365 FABP7 SLITRK5 FOXG1 VIT CA10 HPCAL4
Regulation of hormone levels	9	583	0.029644095	ACHE PCSK1N MYB VGF RDH5 BCO1 TFAP2B KCNJ11 MYRIP

**Down-regulated genes in alive × dead**				
Functional category	Genes in list	Total genes	FDR	Genes
Cornification	17	125	8.55E−15	TMEM79 TGM1 KRT37 PI3 KRT17 KLK14 TCHH KRT82 SPRR2D KLK13 KRT80 JUP KLK12 KRT25 SPINK9 KRT81 SPRR2A
Epithelium development	42	1386	9.40E−15	KLK14 SPRR2D OVOL1 NRARP SPRR2A HGF TGM1 BMP4 FGF7 AJAP1 WNT11 CNFN DLX5 ALOX12 SDC1 ID3 TBX4 KRT17 GREB1L ADAMTSL4 RSPO3 TCHH ELF3 TMEM79 HEYL GREM1 ROR2 SIX2 SOX11 KRT25 SULT2B1 TBX1 RHCG KRT37 PI3 KRT82 KLK13 KRT80 JUP KLK12 SPINK9 KRT81
Epithelial cell differentiation	33	831	1.67E−14	SPRR2D OVOL1 SPRR2A TGM1 BMP4 AJAP1 CNFN DLX5 SDC1 ID3 KRT17 ADAMTSL4 TCHH ELF3 TMEM79 GREM1 SIX2 SOX11 WNT11 SULT2B1 TBX1 RHCG KRT37 PI3 KLK14 KRT82 KLK13 KRT80 JUP KLK12 KRT25 SPINK9 KRT81
Tissue development	51	2168	2.40E−14	KLK14 SPRR2D OVOL1 NRARP SPRR2A HGF TGM1 ZBTB16 BMP4 FGF7 AJAP1 PITX1 WNT11 SMPD3 CNFN DLX5 ALOX12 SDC1 ID3 TBX4 KRT17 GREB1L ADAMTSL4 RSPO3 FGF18 TCHH PKDCC ELF3 TMEM79 HEYL GREM1 ROR2 ADRB2 SIX2 SOX11 KRT25 COL8A1 SULT2B1 SIK1 ACTG2 TBX1 RHCG KRT37 PI3 KRT82 KLK13 KRT80 JUP KLK12 SPINK9 KRT81
Skin development	23	464	1.11E−11	SPRR2D SPRR2A TGM1 CNFN ALOX12 KRT17 FGF7 TCHH TMEM79 OVOL1 JUP ALOX12B KRT25 CLDN4 KRT37 PI3 KLK14 KRT82 KLK13 KRT80 KLK12 SPINK9 KRT81
Keratinization	18	268	4.20E−11	TGM1 CNFN KRT17 TCHH SPRR2D TMEM79 SPRR2A KRT37 PI3 KLK14 KRT82 KLK13 KRT80 JUP KLK12 KRT25 SPINK9 KRT81
Animal organ development	62	3779	4.72E−11	SPRR2D HEYL SIX2 SPRR2A HGF TGM1 ZBTB16 BMP4 FGF7 GREB1L AJAP1 MAFB MYOC USP2 PITX1 NOTCH3 WNT11 ANGPT2 SLC8A3 SMPD3 CNFN DLX5 ALOX12 MDFI SDC1 ID3 TBX4 KRT17 CYP19A1 COL8A1 RSPO3 FGF18 TCHH PKDCC ELF3 TMEM79 ZC3H12A AVPR1A GREM1 ROR2 ADRB2 OVOL1 JUP SOX11 ALOX12B CCBE1 NRARP PAPSS2 KRT25 TBX1 CLDN4 SIK1 ACTG2 KRT37 PI3 KLK14 KRT82 KLK13 KRT80 KLK12 SPINK9 KRT81
Epidermal cell differentiation	21	410	5.29E−11	SPRR2D OVOL1 SPRR2A TGM1 BMP4 CNFN KRT17 TCHH TMEM79 SULT2B1 KRT37 PI3 KLK14 KRT82 KLK13 KRT80 JUP KLK12 KRT25 SPINK9 KRT81
Epidermis development	23	521	6.96E−11	KLK14 SPRR2D OVOL1 SPRR2A TGM1 BMP4 CNFN KRT17 FGF7 TCHH TMEM79 KRT25 SULT2B1 ELF3 KRT37 PI3 KRT82 KLK13 KRT80 JUP KLK12 SPINK9 KRT81
Keratinocyte differentiation	18	346	1.87E−09	SPRR2D SPRR2A TGM1 CNFN KRT17 TCHH TMEM79 KRT37 PI3 KLK14 KRT82 KLK13 KRT80 JUP KLK12 KRT25 SPINK9 KRT81
Ossification	18	396	1.56E−08	MYOC ZBTB16 BMP4 TWIST2 WNT11 SMPD3 DLX5 ID3 GDPD2 FGF18 PKDCC ROR2 ADRB2 SIX2 SOX11 GREM1 CREB3L1 HGF
Cellular developmental process	64	4671	3.21E−08	WNT11 DLX5 ID3 BMP4 NGF SPRR2D ELF3 HEYL OVOL1 RHOD SOX11 SPRR2A MYOC TGM1 ZC3H12A GREM1 AJAP1 MAFB TWIST2 HGF PITX1 NOTCH3 ANGPT2 SLC8A3 SMPD3 CNFN ZBTB16 MDFI SDC1 KRT17 CHN1 GDPD2 CATSPERB CD36 SIK1 ADAMTSL4 FGF18 TCHH FBLIM1 PKDCC TMEM79 AVPR1A ROR2 ADRB2 SIX2 GTSF1 NRARP COL8A1 SULT2B1 CREB3L1 S100A9 TBX1 RHCG KRT37 PI3 KLK14 KRT82 KLK13 KRT80 JUP KLK12 KRT25 SPINK9 KRT81
Cell differentiation	62	4459	3.79E−08	WNT11 DLX5 ID3 BMP4 NGF SPRR2D ELF3 HEYL OVOL1 SOX11 SPRR2A MYOC TGM1 ZC3H12A AJAP1 MAFB TWIST2 HGF PITX1 NOTCH3 ANGPT2 SLC8A3 SMPD3 CNFN ZBTB16 MDFI SDC1 KRT17 CHN1 GDPD2 CATSPERB CD36 SIK1 ADAMTSL4 FGF18 TCHH PKDCC TMEM79 AVPR1A GREM1 ROR2 ADRB2 SIX2 GTSF1 NRARP COL8A1 SULT2B1 CREB3L1 S100A9 TBX1 RHCG KRT37 PI3 KLK14 KRT82 KLK13 KRT80 JUP KLK12 KRT25 SPINK9 KRT81
Anatomical structure morphogenesis	46	2785	1.00E−07	PITX1 DLX5 KLK14 NGF HEYL RHOD NRARP HGF MYOC BMP4 IL1RN FGF7 ZC3H12A GREM1 AJAP1 NOTCH3 WNT11 ANGPT2 SMPD3 ALOX12 ZBTB16 MDFI SDC1 ID3 TBX4 KRT17 CHN1 CD36 GREB1L COL8A1 RSPO3 FGF18 FBLIM1 PKDCC ELF3 TMEM79 ROR2 SIX2 SOX11 CCBE1 MAFB KRT25 CREB3L1 JUP TBX1 ACTG2
Tissue morphogenesis	21	719	8.87E−07	KLK14 NRARP HGF BMP4 AJAP1 WNT11 ALOX12 TBX4 KRT17 FGF7 GREB1L RSPO3 TMEM79 HEYL GREM1 ROR2 SIX2 SOX11 KRT25 ACTG2 TBX1
Regulation of cartilage development	8	68	1.39E−06	ZBTB16 BMP4 SMPD3 FGF18 PKDCC SIX2 GREM1 WNT11
Tube development	25	1062	1.93E−06	NRARP BMP4 FGF7 ZC3H12A HGF NOTCH3 WNT11 ANGPT2 SMPD3 ALOX12 SDC1 TBX4 GREB1L COL8A1 RSPO3 FGF18 PKDCC HEYL GREM1 SIX2 SOX11 CCBE1 CREB3L1 JUP TBX1
Morphogenesis of an epithelium	18	581	3.91E−06	KLK14 NRARP HGF BMP4 AJAP1 WNT11 ALOX12 TBX4 KRT17 FGF7 GREB1L RSPO3 TMEM79 GREM1 ROR2 SIX2 SOX11 KRT25
Urogenital system development	14	346	4.57E−06	SIX2 BMP4 GREB1L NOTCH3 WNT11 ANGPT2 ZBTB16 SDC1 ID3 CYP19A1 HEYL GREM1 OVOL1 SOX11
Kidney development	13	291	4.57E−06	SIX2 BMP4 GREB1L NOTCH3 WNT11 ANGPT2 ZBTB16 SDC1 ID3 HEYL GREM1 OVOL1 SOX11
Regulation of anatomical structure morphogenesis	25	1125	4.57E−06	NGF RHOD HGF MYOC BMP4 IL1RN FGF7 ZC3H12A AJAP1 WNT11 ANGPT2 ALOX12 CHN1 CD36 FBLIM1 GREM1 ROR2 SIX2 CCBE1 NRARP FGF18 CREB3L1 JUP TBX1 RSPO3
Regulation of ossification	11	193	4.57E−06	ZBTB16 BMP4 TWIST2 ID3 GDPD2 PKDCC ADRB2 SIX2 SOX11 GREM1 HGF
Skeletal system development	17	541	6.10E−06	ZBTB16 BMP4 MYOC PITX1 SMPD3 DLX5 MDFI FGF18 PKDCC ROR2 SIX2 SOX11 PAPSS2 WNT11 TBX4 GREM1 TBX1
Renal system development	13	307	7.35E−06	SIX2 BMP4 GREB1L NOTCH3 WNT11 ANGPT2 ZBTB16 SDC1 ID3 HEYL GREM1 OVOL1 SOX11
Animal organ morphogenesis	24	1099	1.06E−05	HEYL HGF BMP4 FGF7 AJAP1 WNT11 SMPD3 DLX5 MDFI SDC1 ID3 GREB1L COL8A1 FGF18 ELF3 GREM1 ROR2 SIX2 SOX11 MAFB TBX4 TBX1 ACTG2 RSPO3
Osteoblast differentiation	11	218	1.18E−05	MYOC BMP4 TWIST2 WNT11 DLX5 ID3 GDPD2 GREM1 SOX11 CREB3L1 HGF
Regulation of developmental process	41	2763	1.21E−05	ID3 NGF HEYL RHOD HGF MYOC ZBTB16 BMP4 IL1RN FGF7 ZC3H12A AJAP1 MAFB TWIST2 NOTCH3 WNT11 ANGPT2 SMPD3 ALOX12 KRT17 CHN1 GDPD2 CD36 FGF18 FBLIM1 PKDCC TMEM79 GREM1 ROR2 ADRB2 SIX2 SOX11 CCBE1 NRARP CREB3L1 JUP SULT2B1 SIK1 S100A9 TBX1 RSPO3
Anatomical structure formation involved in morphogenesis	24	1164	2.67E−05	DLX5 NRARP BMP4 ZC3H12A HGF NOTCH3 WNT11 ANGPT2 SMPD3 TBX4 CD36 COL8A1 RSPO3 FGF18 HEYL GREM1 ROR2 SIX2 SOX11 CCBE1 MAFB CREB3L1 JUP TBX1
Skeletal system morphogenesis	11	245	3.39E−05	SMPD3 DLX5 MDFI BMP4 FGF18 ROR2 SIX2 SOX11 TBX4 GREM1 TBX1
Regulation of chondrocyte differentiation	6	49	3.91E−05	BMP4 FGF18 PKDCC SIX2 ZBTB16 GREM1
Appendage morphogenesis	9	155	3.91E−05	PITX1 DLX5 ZBTB16 TBX4 BMP4 PKDCC GREM1 ROR2 SOX11
Limb morphogenesis	9	155	3.91E−05	PITX1 DLX5 ZBTB16 TBX4 BMP4 PKDCC GREM1 ROR2 SOX11
Tube morphogenesis	20	860	3.91E−05	NRARP BMP4 ZC3H12A HGF NOTCH3 WNT11 ANGPT2 ALOX12 TBX4 GREB1L COL8A1 RSPO3 FGF18 GREM1 SIX2 SOX11 CCBE1 CREB3L1 JUP TBX1
Regulation of morphogenesis of an epithelium	10	204	4.32E−05	HGF BMP4 AJAP1 WNT11 ALOX12 FGF7 GREM1 ROR2 SIX2 RSPO3
Epidermis morphogenesis	5	29	5.24E−05	KLK14 KRT17 FGF7 TMEM79 KRT25
Angiogenesis	15	511	5.42E−05	NRARP BMP4 ZC3H12A HGF NOTCH3 ANGPT2 TBX4 COL8A1 RSPO3 FGF18 GREM1 CCBE1 CREB3L1 JUP TBX1
Cell migration	27	1506	5.42E−05	HGF SDC1 IL36G IL1RN DEFB4A RHOD MYOC ANGPT2 BMP4 FGF7 FGF18 S100A9 ZC3H12A ROR2 SIX2 WNT11 BDKRB1 SMPD3 ALOX12 CYP19A1 GREM1 CCBE1 LTB4R2 JUP TWIST2 TBX1 S100A12
Cell death	36	2415	5.45E−05	HGF BMP4 NGF ADAMTSL4 ZNF385B S100A9 ZC3H12A WNT11 ALOX12 ZBTB16 ID3 CREB3L1 TMEM79 GREM1 SOX11 TWIST2 CD36 SIK1 TBX1 PNMA5 TGM1 KRT37 PI3 KRT17 KLK14 TCHH KRT82 SPRR2D KLK13 KRT80 JUP KLK12 KRT25 SPINK9 KRT81 SPRR2A
Respiratory system development	10	215	6.07E−05	BMP4 FGF7 SMPD3 DLX5 TBX4 FGF18 PKDCC SOX11 CCBE1 WNT11
Cartilage development	10	216	6.17E−05	ZBTB16 BMP4 PITX1 SMPD3 FGF18 PKDCC ROR2 SIX2 GREM1 WNT11
Positive regulation of ossification	7	88	6.78E−05	ZBTB16 BMP4 GDPD2 PKDCC ADRB2 SOX11 HGF
Regulation of multicellular organismal development	33	2138	7.25E−05	ID3 NGF HEYL HGF ZBTB16 BMP4 IL1RN FGF7 ZC3H12A AJAP1 MAFB NOTCH3 WNT11 ANGPT2 SMPD3 ALOX12 KRT17 CHN1 FGF18 PKDCC GREM1 ROR2 ADRB2 SIX2 SOX11 CCBE1 NRARP CREB3L1 JUP SULT2B1 S100A9 TBX1 RSPO3
Blood vessel morphogenesis	16	603	7.46E−5	NRARP BMP4 ZC3H12A HGF NOTCH3 WNT11 ANGPT2 TBX4 COL8A1 RSPO3 FGF18 GREM1 CCBE1 CREB3L1 JUP TBX1
Programmed cell death	34	2257	8.00E−05	HGF BMP4 NGF ADAMTSL4 ZNF385B S100A9 WNT11 ALOX12 ZBTB16 ID3 CREB3L1 TMEM79 ZC3H12A GREM1 TWIST2 SIK1 TBX1 PNMA5 TGM1 KRT37 PI3 KRT17 KLK14 TCHH KRT82 SPRR2D KLK13 KRT80 JUP KLK12 KRT25 SPINK9 KRT81 SPRR2A
Blood vessel development	17	687	8.58E−05	HEYL NRARP BMP4 ZC3H12A HGF NOTCH3 WNT11 ANGPT2 TBX4 COL8A1 RSPO3 FGF18 GREM1 CCBE1 CREB3L1 JUP TBX1
Embryonic morphogenesis	16	615	8.98E−05	BMP4 IL1RN PITX1 WNT11 DLX5 ZBTB16 MDFI TBX4 RSPO3 GREM1 ROR2 SIX2 SOX11 MAFB COL8A1 TBX1
Cell motility	28	1670	9.30E−05	HGF SDC1 IL36G IL1RN DEFB4A RHOD MYOC ANGPT2 BMP4 FGF7 FGF18 S100A9 ZC3H12A ROR2 SIX2 WNT11 BDKRB1 SMPD3 ALOX12 CYP19A1 GREM1 CCBE1 LTB4R2 DUOX1 JUP TWIST2 TBX1 S100A12
Localization of cell	28	1670	9.30E−05	HGF SDC1 IL36G IL1RN DEFB4A RHOD MYOC ANGPT2 BMP4 FGF7 FGF18 S100A9 ZC3H12A ROR2 SIX2 WNT11 BDKRB1 SMPD3 ALOX12 CYP19A1 GREM1 CCBE1 LTB4R2 DUOX1 JUP TWIST2 TBX1 S100A12
Cellular response to growth factor stimulus	17	694	9.30E−05	HGF BMP4 NGF FGF18 CREB3L1 GREM1 CCBE1 FAM83G ANGPT2 SMPD3 DLX5 RASL11B FGF7 HEYL ROR2 SOX11 TBX1
Cell–cell signaling	29	1774	9.65E−05	WNT11 CHRNA4 NGF RSPO3 NRARP JUP HGF CYP19A1 GREM1 FAM3D SLC8A3 SMPD3 DLX5 SDC1 BMP4 IL1RN FGF7 ROR2 ADRB2 SOX11 LYNX1 MYOC G6PC2 MDFI GLP1R IL36G FGF18 S100A9 ADRA1D
Tissue migration	11	293	1.10E−04	ANGPT2 BMP4 FGF7 FGF18 ZC3H12A ALOX12 GREM1 CCBE1 LTB4R2 JUP ACTG2
Appendage development	9	186	1.10E−04	PITX1 DLX5 ZBTB16 TBX4 BMP4 PKDCC GREM1 ROR2 SOX11
Limb development	9	186	1.10E−04	PITX1 DLX5 ZBTB16 TBX4 BMP4 PKDCC GREM1 ROR2 SOX11
Lung development	9	188	1.18E−04	BMP4 FGF7 SMPD3 TBX4 FGF18 PKDCC SOX11 CCBE1 WNT11
Vasculature development	17	715	1.20E−04	HEYL NRARP BMP4 ZC3H12A HGF NOTCH3 WNT11 ANGPT2 TBX4 COL8A1 RSPO3 FGF18 GREM1 CCBE1 CREB3L1 JUP TBX1
Respiratory tube development	9	192	1.34E−04	BMP4 FGF7 SMPD3 TBX4 FGF18 PKDCC SOX11 CCBE1 WNT11
Response to growth factor	17	723	1.34E−04	HGF BMP4 NGF FGF18 CREB3L1 GREM1 CCBE1 FAM83G ANGPT2 SMPD3 DLX5 RASL11B FGF7 HEYL ROR2 SOX11 TBX1
Cardiovascular system development	17	724	1.34E−04	HEYL NRARP BMP4 ZC3H12A HGF NOTCH3 WNT11 ANGPT2 TBX4 COL8A1 RSPO3 FGF18 GREM1 CCBE1 CREB3L1 JUP TBX1
Locomotion	30	1921	1.34E−04	HGF SDC1 IL36G IL1RN DEFB4A RHOD MYOC ANGPT2 BMP4 FGF7 FGF18 S100A9 ZC3H12A ROR2 SIX2 WNT11 BDKRB1 SMPD3 DLX5 ALOX12 CHN1 CYP19A1 GREM1 CCBE1 LTB4R2 DUOX1 JUP TWIST2 TBX1 S100A12
Mesonephros development	7	104	1.40E−04	BMP4 ZBTB16 SDC1 GREB1L GREM1 SIX2 WNT11
Positive regulation of developmental process	25	1433	1.40E−04	NGF MYOC ZBTB16 BMP4 FGF7 ZC3H12A HGF ANGPT2 ALOX12 KRT17 GDPD2 CD36 FGF18 PKDCC TMEM79 HEYL GREM1 ROR2 ADRB2 SOX11 CCBE1 JUP SULT2B1 S100A9 TBX1
Nephron development	8	147	1.41E−04	BMP4 NOTCH3 ANGPT2 GREB1L HEYL GREM1 SIX2 WNT11
Positive regulation of cell communication	30	1937	1.48E−04	BMP4 IL36G IL1RN RSPO3 NRARP JUP HGF MYOC KLK14 CYP19A1 FGF18 S100A9 S100A12 ZC3H12A ADRB2 ALOX12B CCBE1 SLC8A3 DLX5 CHN1 CD36 GREM1 ROR2 SOX11 WNT11 SH3RF3 NGF TBX1 FGF7 ELF3
Positive regulation of signaling	30	1945	1.56E−04	BMP4 IL36G IL1RN RSPO3 NRARP JUP HGF MYOC KLK14 CYP19A1 FGF18 S100A9 S100A12 ZC3H12A ADRB2 ALOX12B CCBE1 SLC8A3 DLX5 CHN1 CD36 GREM1 ROR2 SOX11 WNT11 SH3RF3 NGF TBX1 FGF7 ELF3
Circulatory system development	21	1077	1.56E−04	HEYL NRARP BMP4 ZC3H12A HGF NOTCH3 WNT11 ANGPT2 ID3 TBX4 GREB1L COL8A1 RSPO3 FGF18 GREM1 SOX11 CCBE1 CREB3L1 JUP TBX1 SIK1
Positive regulation of signal transduction	28	1762	1.91E−04	BMP4 IL36G IL1RN RSPO3 NRARP JUP HGF MYOC KLK14 FGF18 S100A9 S100A12 ZC3H12A ADRB2 ALOX12B CCBE1 DLX5 CHN1 CD36 GREM1 ROR2 SOX11 WNT11 SH3RF3 NGF TBX1 FGF7 ELF3
Ameboidal-type cell migration	12	392	2.39E−04	ANGPT2 BMP4 FGF7 FGF18 ZC3H12A WNT11 ALOX12 GREM1 CCBE1 LTB4R2 JUP TBX1
Regulation of osteoblast differentiation	7	115	2.44E−04	BMP4 TWIST2 ID3 GDPD2 GREM1 SOX11 HGF
Bone mineralization	7	116	2.55E−04	BMP4 WNT11 SMPD3 PKDCC ROR2 ADRB2 GREM1
Establishment of skin barrier	4	22	2.65E−04	ALOX12 TMEM79 ALOX12B CLDN4
Regulation of cell motility	19	946	2.69E−04	HGF RHOD MYOC ANGPT2 BMP4 FGF7 FGF18 ZC3H12A ROR2 BDKRB1 SMPD3 ALOX12 CYP19A1 GREM1 CCBE1 DUOX1 JUP TWIST2 WNT11
Mesenchymal cell proliferation	5	46	2.69E−04	BMP4 FGF7 WNT11 SIX2 TBX1
Positive regulation of cell motility	14	544	2.73E−04	HGF MYOC BMP4 FGF7 FGF18 ZC3H12A ROR2 BDKRB1 ALOX12 CCBE1 DUOX1 TWIST2 WNT11 RHOD
Response to BMP	8	165	2.73E−04	BMP4 GREM1 FAM83G SMPD3 DLX5 HEYL ROR2 SOX11
Cellular response to BMP stimulus	8	165	2.73E−04	BMP4 GREM1 FAM83G SMPD3 DLX5 HEYL ROR2 SOX11
Biomineral tissue development	8	167	2.93E−04	BMP4 WNT11 SMPD3 PKDCC ROR2 ADRB2 GREM1 TBX1
Connective tissue development	10	280	3.18E−04	ZBTB16 BMP4 PITX1 SMPD3 FGF18 PKDCC ROR2 SIX2 GREM1 WNT11
Regulation of animal organ morphogenesis	10	281	3.22E−04	HGF BMP4 FGF7 AJAP1 WNT11 GREM1 ROR2 SIX2 TBX1 RSPO3
Embryo development	20	1054	3.22E−04	BMP4 IL1RN PITX1 WNT11 DLX5 ZBTB16 MDFI ID3 TBX4 RSPO3 PKDCC ELF3 GREM1 ROR2 SIX2 SOX11 NRARP MAFB COL8A1 TBX1
Chondrocyte differentiation	7	123	3.22E−04	SMPD3 BMP4 FGF18 PKDCC SIX2 ZBTB16 GREM1
Epithelial cell migration	10	284	3.33E−04	ANGPT2 BMP4 FGF7 FGF18 ZC3H12A ALOX12 GREM1 CCBE1 LTB4R2 JUP
Regulation of cell migration	18	883	3.33E−04	HGF RHOD MYOC ANGPT2 BMP4 FGF7 FGF18 ZC3H12A ROR2 BDKRB1 SMPD3 ALOX12 CYP19A1 GREM1 CCBE1 JUP TWIST2 WNT11
Positive regulation of cellular component movement	14	558	3.33E−04	HGF MYOC BMP4 FGF7 FGF18 ZC3H12A ROR2 BDKRB1 ALOX12 CCBE1 DUOX1 TWIST2 WNT11 RHOD
Epithelium migration	10	287	3.60E−04	ANGPT2 BMP4 FGF7 FGF18 ZC3H12A ALOX12 GREM1 CCBE1 LTB4R2 JUP
Hair follicle morphogenesis	4	25	3.68E−04	KRT17 FGF7 TMEM79 KRT25
Regulation of water loss via skin	4	25	3.68E−04	ALOX12 TMEM79 ALOX12B CLDN4
Embryonic limb morphogenesis	7	130	4.20E−04	PITX1 DLX5 ZBTB16 TBX4 BMP4 GREM1 ROR2
Embryonic appendage morphogenesis	7	130	4.20E−04	PITX1 DLX5 ZBTB16 TBX4 BMP4 GREM1 ROR2
Positive regulation of locomotion	14	576	4.34E−04	HGF MYOC BMP4 FGF7 FGF18 ZC3H12A ROR2 BDKRB1 ALOX12 CCBE1 DUOX1 TWIST2 WNT11 RHOD
Endochondral ossification	4	27	4.78E−04	SMPD3 DLX5 BMP4 FGF18
Replacement ossification	4	27	4.78E−04	SMPD3 DLX5 BMP4 FGF18
Positive regulation of MAPK cascade	14	583	4.79E−04	IL36G IL1RN BMP4 FGF18 ZC3H12A ADRB2 ALOX12B HGF CD36 ROR2 SH3RF3 TBX1 NGF S100A12
Poly-N-acetyllactosamine biosynthetic process	3	10	5.48E−04	B3GNT4 B3GNT8 B3GNT3
Regulation of multicellular organismal process	41	3382	5.95E−04	ID3 NGF IL36G IL1RN HEYL AVPR1A ADRB2 HGF ANGPT2 ZBTB16 BMP4 FGF7 FGF18 ZC3H12A CCBE1 AJAP1 MAFB TWIST2 NOTCH3 WNT11 SLC8A3 SMPD3 ALOX12 GLP1R KRT17 CHN1 GDPD2 CD36 PKDCC GREM1 ROR2 SIX2 SOX11 NRARP CREB3L1 JUP SULT2B1 ALOX12B S100A9 TBX1 RSPO3
Response to endogenous stimulus	26	1704	5.97E−04	BMP4 NGF AVPR1A IL1RN FGF18 CREB3L1 HEYL GREM1 JUP FAM83G SLC8A3 CHRNA4 SMPD3 DLX5 GLP1R SDC1 RASL11B CD36 FGF7 ROR2 ADRB2 SOX11 CLDN4 TBX1 GNA15 CATSPERB
Positive regulation of cell migration	13	521	6.09E−04	HGF MYOC BMP4 FGF7 FGF18 ZC3H12A ROR2 BDKRB1 ALOX12 CCBE1 TWIST2 WNT11 RHOD
Chemotaxis	15	680	6.18E−04	IL36G IL1RN DEFB4A HGF ANGPT2 FGF7 FGF18 S100A9 DLX5 BMP4 CHN1 CYP19A1 GREM1 S100A12 LTB4R2
Regulation of cellular component movement	19	1028	6.21E−04	HGF RHOD MYOC ANGPT2 BMP4 FGF7 FGF18 ZC3H12A ROR2 BDKRB1 SMPD3 ALOX12 CYP19A1 GREM1 CCBE1 DUOX1 JUP TWIST2 WNT11
Movement of cell or subcellular component	30	2139	6.25E−04	HGF SDC1 IL36G IL1RN DEFB4A RHOD MYOC ANGPT2 BMP4 FGF7 FGF18 S100A9 ZC3H12A ROR2 SIX2 WNT11 BDKRB1 SMPD3 DLX5 ALOX12 CHN1 CYP19A1 GREM1 CCBE1 LTB4R2 DUOX1 JUP TWIST2 TBX1 S100A12
Taxis	15	683	6.25E−04	IL36G IL1RN DEFB4A HGF ANGPT2 FGF7 FGF18 S100A9 DLX5 BMP4 CHN1 CYP19A1 GREM1 S100A12 LTB4R2
Nephron tubule development	6	96	6.25E−04	BMP4 GREB1L HEYL GREM1 SIX2 WNT11
Embryonic organ development	12	452	6.34E−04	WNT11 DLX5 MDFI ID3 BMP4 RSPO3 PKDCC ROR2 SIX2 SOX11 MAFB TBX1
Morphogenesis of a branching epithelium	8	194	6.34E−04	NRARP HGF BMP4 FGF7 GREB1L RSPO3 GREM1 SIX2
Kidney epithelium development	7	143	6.55E−04	BMP4 SDC1 GREB1L HEYL GREM1 SIX2 WNT11
Poly-N-acetyllactosamine metabolic process	3	11	6.64E−04	B3GNT4 B3GNT8 B3GNT3
Positive regulation of intracellular signal transduction	20	1133	6.72E−04	IL36G IL1RN HGF MYOC BMP4 FGF18 S100A9 S100A12 ZC3H12A ADRB2 ALOX12B CD36 GREM1 ROR2 SOX11 WNT11 SH3RF3 NGF TBX1 FGF7
Regulation of locomotion	19	1041	6.74E−04	HGF RHOD MYOC ANGPT2 BMP4 FGF7 FGF18 ZC3H12A ROR2 BDKRB1 SMPD3 ALOX12 CYP19A1 GREM1 CCBE1 DUOX1 JUP TWIST2 WNT11
Ureteric bud development	6	99	6.85E−04	BMP4 SDC1 GREB1L GREM1 SIX2 WNT11
Renal tubule development	6	99	6.85E−04	BMP4 GREB1L HEYL GREM1 SIX2 WNT11
Positive regulation of cartilage development	4	31	6.94E−04	ZBTB16 BMP4 FGF18 PKDCC
Mesonephric epithelium development	6	100	7.06E−04	BMP4 SDC1 GREB1L GREM1 SIX2 WNT11
Mesonephric tubule development	6	100	7.06E−04	BMP4 SDC1 GREB1L GREM1 SIX2 WNT11
Positive regulation of gene expression	29	2060	7.22E−04	DLX5 ZBTB16 HEYL JUP SOX11 BMP4 ELF3 ZC3H12A CCBE1 PITX1 NOTCH3 ALOX12 KRT17 CD36 FGF7 CREB3L1 GREM1 ROR2 ADRB2 SIX2 OVOL1 MAFB WNT11 NGF ALOX12B GLIS3 ACTG2 TBX1 HGF
Regulation of morphogenesis of a branching structure	5	63	7.98E−04	HGF BMP4 FGF7 GREM1 SIX2
BMP signaling pathway	7	152	8.66E−04	BMP4 GREM1 FAM83G SMPD3 DLX5 ROR2 SOX11
Endothelial cell migration	8	206	8.66E−04	ANGPT2 BMP4 FGF18 ZC3H12A ALOX12 GREM1 CCBE1 JUP
Morphogenesis of a branching structure	8	208	9.13E−04	NRARP HGF BMP4 FGF7 GREB1L RSPO3 GREM1 SIX2
Mesenchymal to epithelial transition involved in metanephros morphogenesis	3	13	1.01E−03	BMP4 GREM1 SIX2
Mesenchymal cell differentiation	8	212	1.01E−03	WNT11 BMP4 HEYL SIX2 SOX11 GREM1 TBX1 HGF
Mesenchyme development	9	273	1.01E−03	WNT11 BMP4 HEYL SIX2 SOX11 GREM1 ACTG2 TBX1 HGF
Ureteric bud morphogenesis	5	67	1.01E−03	BMP4 GREB1L GREM1 SIX2 WNT11
Mesonephric tubule morphogenesis	5	68	1.08E−03	BMP4 GREB1L GREM1 SIX2 WNT11
Regulation of cell-substrate adhesion	8	216	1.13E−03	MYOC CD36 AJAP1 ANGPT2 COL8A1 GREM1 RHOD JUP
Regulation of endothelial cell migration	7	161	1.16E−03	ANGPT2 BMP4 FGF18 ZC3H12A ALOX12 CCBE1 JUP
Positive regulation of multicellular organismal process	27	1911	1.17E−03	NGF IL36G IL1RN AVPR1A ZBTB16 BMP4 FGF7 FGF18 ZC3H12A CCBE1 HGF WNT11 ANGPT2 ALOX12 KRT17 GDPD2 CD36 PKDCC HEYL GREM1 ROR2 ADRB2 SOX11 JUP ALOX12B S100A9 TBX1
Nephron epithelium development	6	112	1.18E−03	BMP4 GREB1L HEYL GREM1 SIX2 WNT11
Regulation of epithelial cell migration	8	223	1.36E−03	ANGPT2 BMP4 FGF7 FGF18 ZC3H12A ALOX12 CCBE1 JUP
Positive regulation of epidermis development	4	38	1.36E−03	BMP4 KRT17 TMEM79 SULT2B1
Aminoglycan biosynthetic process	6	116	1.39E−03	B3GNT4 B3GNT8 B3GNT3 SMPD3 SDC1 HS3ST3A1
Positive regulation of angiogenesis	7	167	1.39E−03	ZC3H12A HGF ANGPT2 GREM1 CCBE1 FGF18 JUP
Sprouting angiogenesis	6	117	1.45E−03	NRARP BMP4 RSPO3 GREM1 CREB3L1 CCBE1
Cell-substrate adhesion	10	358	1.46E−03	LYPD3 LYPD5 MYOC CD36 AJAP1 ANGPT2 COL8A1 GREM1 RHOD JUP
Sulfate assimilation	2	3	1.52E−03	PAPSS2 SULT2B1
Cuticle development	2	3	1.52E−03	TMEM79 DUOX1
Cloacal septation	2	3	1.52E−03	BMP4 WNT11
Aminoglycan metabolic process	7	171	1.53E−03	B3GNT4 B3GNT8 B3GNT3 HGF SMPD3 SDC1 HS3ST3A1
Cardiac septum morphogenesis	5	75	1.53E−03	HEYL WNT11 BMP4 SOX11 TBX1
Cellular response to endogenous stimulus	22	1432	1.61E−03	BMP4 NGF AVPR1A FGF18 CREB3L1 HEYL GREM1 JUP FAM83G SLC8A3 CHRNA4 SMPD3 DLX5 GLP1R RASL11B FGF7 ROR2 ADRB2 SOX11 CD36 TBX1 GNA15
Nephron tubule morphogenesis	5	76	1.61E−03	BMP4 GREB1L GREM1 SIX2 WNT11
Metanephric renal vesicle morphogenesis	3	16	1.65E−03	BMP4 GREM1 SIX2
Regulation of cell–matrix adhesion	6	122	1.68E−03	MYOC CD36 AJAP1 GREM1 RHOD JUP
Outflow tract morphogenesis	5	77	1.68E−03	WNT11 BMP4 HEYL SOX11 TBX1
Nephron epithelium morphogenesis	5	78	1.77E−03	BMP4 GREB1L GREM1 SIX2 WNT11
Ventricular septum morphogenesis	4	42	1.79E−03	HEYL WNT11 BMP4 SOX11
Cell–matrix adhesion	8	239	1.91E−03	LYPD3 LYPD5 MYOC CD36 AJAP1 GREM1 RHOD JUP
Embryonic organ morphogenesis	9	305	1.91E−03	WNT11 DLX5 MDFI BMP4 ROR2 SIX2 SOX11 MAFB TBX1
Renal tubule morphogenesis	5	80	1.91E−03	BMP4 GREB1L GREM1 SIX2 WNT11
Nephron morphogenesis	5	80	1.91E−03	BMP4 GREB1L GREM1 SIX2 WNT11
Nephric duct development	3	17	1.91E−03	BMP4 GREB1L WNT11
Regulation of cell communication	43	3903	2.15E−03	BMP4 NGF IL36G IL1RN SIK1 RSPO3 NRARP JUP HGF MYOC KLK14 CYP19A1 FGF18 CREB3L1 S100A9 S100A12 ZC3H12A HEYL GREM1 ADRB2 ALOX12B LYNX1 CCBE1 FAM3D WNT11 SLC8A3 CHRNA4 DLX5 RASL11B CHN1 CD36 FGF7 AVPR1A ROR2 SOX11 G6PC2 SH3RF3 MDFI TBX1 NOTCH3 GLP1R ELF3 RHOD
Enzyme linked receptor protein signaling pathway	18	1072	2.17E−03	HGF BMP4 NGF ROR2 ANGPT2 CREB3L1 GREM1 CCBE1 FAM83G MYOC SMPD3 DLX5 RASL11B FGF7 FGF18 SOX11 CHN1 ADRB2
Regulation of MAPK cascade	15	793	2.17E−03	BMP4 IL36G IL1RN MYOC FGF18 ZC3H12A ADRB2 ALOX12B HGF CD36 ROR2 SH3RF3 TBX1 NGF S100A12
Embryonic skeletal system development	6	130	2.21E−03	MDFI BMP4 SIX2 SOX11 WNT11 TBX1
Positive regulation of vasculature development	7	186	2.29E−03	ZC3H12A HGF ANGPT2 GREM1 CCBE1 FGF18 JUP
Response to organic substance	40	3547	2.30E−03	HGF BMP4 NGF IL36G IL1RN AVPR1A CD36 DUOX1 FGF18 CREB3L1 ZC3H12A HEYL GREM1 JUP CCBE1 FAM83G ANGPT2 SLC8A3 BDKRB1 CHRNA4 SMPD3 DLX5 ALOX12 GLP1R SDC1 ID3 SLPI RASL11B FGF7 ROR2 ADRB2 ABCG4 SOX11 CLDN4 WNT11 TBX1 GNA15 CATSPERB HSPB3 GPX3
Positive chemotaxis	5	84	2.30E−03	ANGPT2 FGF7 HGF BMP4 DEFB4A
Response to psychosocial stress	2	4	2.56E−03	GLP1R ADRB2
Cloaca development	2	4	2.56E−03	BMP4 WNT11
Positive regulation of epithelial cell proliferation	7	190	2.56E−03	NRARP BMP4 FGF7 DLX5 SOX11 TGM1 TBX1
Regulation of branching involved in salivary gland morphogenesis by mesenchymal-epithelial signaling	2	4	2.56E−03	HGF FGF7
Myoblast differentiation	5	87	2.62E−03	BMP4 PITX1 SDC1 ID3 GREM1
Regulation of signaling	43	3952	2.64E−03	BMP4 NGF IL36G IL1RN SIK1 RSPO3 NRARP JUP HGF MYOC KLK14 CYP19A1 FGF18 CREB3L1 S100A9 S100A12 ZC3H12A HEYL GREM1 ADRB2 ALOX12B LYNX1 CCBE1 FAM3D WNT11 SLC8A3 CHRNA4 DLX5 RASL11B CHN1 CD36 FGF7 AVPR1A ROR2 SOX11 G6PC2 SH3RF3 MDFI TBX1 NOTCH3 GLP1R ELF3 RHOD
Negative regulation of transcription, DNA-templated	20	1298	2.73E−03	ZBTB16 BMP4 SIX2 OVOL1 SOX11 ZNF154 ID3 CREB3L1 ELF3 HEYL PITX1 NOTCH3 CD36 GREM1 NRARP USP2 WNT11 GLIS3 MDFI TWIST2
Positive regulation of chondrocyte differentiation	3	20	2.78E−03	FGF18 PKDCC ZBTB16
Mesenchymal to epithelial transition	3	20	2.78E−03	BMP4 GREM1 SIX2
Renal vesicle morphogenesis	3	20	2.78E−03	BMP4 GREM1 SIX2
Response to oxygen-containing compound	24	1725	2.88E−03	IL36G IL1RN HGF DUOX1 ZC3H12A JUP ANGPT2 SLC8A3 BDKRB1 CHRNA4 SMPD3 GLP1R SDC1 ID3 SLPI CD36 AVPR1A ADRB2 CLDN4 WNT11 TBX1 GNA15 CATSPERB GPX3
Regulation of vasculature development	9	328	2.89E−03	BMP4 ZC3H12A HGF ANGPT2 GREM1 CCBE1 FGF18 CREB3L1 JUP
Digestive tract morphogenesis	4	50	3.01E−03	BMP4 SIX2 SOX11 WNT11
Metanephros development	5	91	3.04E−03	BMP4 ID3 GREB1L GREM1 SIX2
Positive regulation of epithelial cell migration	6	141	3.04E−03	BMP4 FGF7 FGF18 ZC3H12A ALOX12 CCBE1
Regulation of cell differentiation	26	1954	3.04E−03	ID3 NGF HEYL MYOC BMP4 ZC3H12A AJAP1 MAFB TWIST2 HGF NOTCH3 ZBTB16 CHN1 GDPD2 CD36 FGF18 PKDCC GREM1 ROR2 SIX2 SOX11 NRARP SULT2B1 SIK1 S100A9 TBX1
Copulation	3	21	3.06E−03	KLK14 PI3 AVPR1A
Positive regulation of cell differentiation	17	1018	3.06E−03	NGF MYOC BMP4 ZC3H12A HGF ZBTB16 GDPD2 CD36 FGF18 PKDCC HEYL GREM1 ROR2 SOX11 SULT2B1 S100A9 TBX1
Renal vesicle development	3	21	3.06E−03	BMP4 GREM1 SIX2
Cell surface receptor signaling pathway involved in cell–cell signaling	13	654	3.16E−03	WNT11 RSPO3 NRARP JUP GREM1 CHRNA4 DLX5 SDC1 ROR2 ADRB2 MYOC BMP4 MDFI
Roof of mouth development	5	93	3.25E−03	WNT11 DLX5 PKDCC SOX11 TBX1
Leukocyte migration	11	491	3.36E−03	IL36G IL1RN S100A9 BDKRB1 SMPD3 CYP19A1 GREM1 ROR2 ANGPT2 SDC1 S100A12
Cell chemotaxis	9	339	3.45E−03	IL36G IL1RN DEFB4A HGF FGF18 S100A9 CYP19A1 GREM1 S100A12
Sensory organ morphogenesis	8	270	3.53E−03	DLX5 BMP4 COL8A1 ROR2 SIX2 SOX11 MAFB TBX1
Negative regulation of cellular biosynthetic process	23	1658	3.67E−03	ZBTB16 BMP4 SIX2 OVOL1 SOX11 ZNF154 ID3 CREB3L1 ELF3 ZC3H12A HEYL PITX1 NOTCH3 SMPD3 CD36 GREM1 NRARP USP2 WNT11 GLIS3 MDFI SIK1 TWIST2
Hepoxilin metabolic process	2	5	3.67E−03	ALOX12 ALOX12B
Hepoxilin biosynthetic process	2	5	3.67E−03	ALOX12 ALOX12B
Glomerulus vasculature morphogenesis	2	5	3.67E−03	NOTCH3 BMP4
Glomerular capillary formation	2	5	3.67E−03	NOTCH3 BMP4
Negative regulation of RNA metabolic process	21	1446	3.68E−03	ZBTB16 BMP4 SIX2 OVOL1 SOX11 ZNF154 ID3 CREB3L1 ELF3 ZC3H12A HEYL PITX1 NOTCH3 CD36 GREM1 NRARP USP2 WNT11 GLIS3 MDFI TWIST2
Embryonic skeletal system morphogenesis	5	97	3.70E−03	MDFI BMP4 SIX2 SOX11 TBX1
Kidney morphogenesis	5	97	3.70E−03	BMP4 GREB1L GREM1 SIX2 WNT11
Cellular response to chemical stimulus	39	3536	3.70E−03	HGF BMP4 NGF IL36G IL1RN AVPR1A DEFB4A DUOX1 FGF18 CREB3L1 S100A9 S100A12 ZC3H12A HEYL GREM1 JUP CCBE1 FAM83G ANGPT2 SLC8A3 CHRNA4 SMPD3 DLX5 ALOX12 GLP1R ID3 RASL11B CD36 CYP19A1 FGF7 ROR2 ADRB2 ABCG4 SOX11 GPX3 WNT11 TBX1 GNA15 SDC1
Positive regulation of endothelial cell migration	5	98	3.86E−03	BMP4 FGF18 ZC3H12A ALOX12 CCBE1
Regulation of cell adhesion	14	765	3.94E−03	MYOC CD36 IL1RN AJAP1 ANGPT2 ALOX12 ZBTB16 COL8A1 ZC3H12A GREM1 RHOD NRARP JUP BMP4
Negative regulation of nucleic acid-templated transcription	20	1353	3.96E−03	ZBTB16 BMP4 SIX2 OVOL1 SOX11 ZNF154 ID3 CREB3L1 ELF3 HEYL PITX1 NOTCH3 CD36 GREM1 NRARP USP2 WNT11 GLIS3 MDFI TWIST2
Cellular response to organic substance	34	2938	3.99E−03	HGF BMP4 NGF IL36G IL1RN AVPR1A DUOX1 FGF18 CREB3L1 ZC3H12A HEYL GREM1 JUP CCBE1 FAM83G ANGPT2 SLC8A3 CHRNA4 SMPD3 DLX5 ALOX12 GLP1R ID3 RASL11B CD36 FGF7 ROR2 ADRB2 ABCG4 SOX11 WNT11 TBX1 GNA15 SDC1
Negative regulation of RNA biosynthetic process	20	1355	3.99E−03	ZBTB16 BMP4 SIX2 OVOL1 SOX11 ZNF154 ID3 CREB3L1 ELF3 HEYL PITX1 NOTCH3 CD36 GREM1 NRARP USP2 WNT11 GLIS3 MDFI TWIST2
Regulation of branching involved in ureteric bud morphogenesis	3	24	4.06E−03	BMP4 GREM1 SIX2
Intermediate filament organization	3	24	4.06E−03	TCHH KRT17 KRT25
Positive regulation of transcription by RNA polymerase II	19	1255	4.06E−03	DLX5 ZBTB16 HEYL JUP SOX11 BMP4 ZC3H12A PITX1 NOTCH3 CREB3L1 ELF3 GREM1 ADRB2 SIX2 OVOL1 MAFB GLIS3 TBX1 HGF
Glomerulus vasculature development	3	24	4.06E−03	BMP4 NOTCH3 ANGPT2
Negative regulation of biosynthetic process	23	1681	4.10E−03	ZBTB16 BMP4 SIX2 OVOL1 SOX11 ZNF154 ID3 CREB3L1 ELF3 ZC3H12A HEYL PITX1 NOTCH3 SMPD3 CD36 GREM1 NRARP USP2 WNT11 GLIS3 MDFI SIK1 TWIST2
Carbohydrate biosynthetic process	7	214	4.10E−03	G6PC2 B3GNT4 B3GNT8 B3GNT3 SMPD3 CHST8 SIK1
Positive regulation of protein phosphorylation	17	1057	4.10E−03	HGF BMP4 IL36G IL1RN FGF7 FGF18 ZC3H12A ADRB2 ALOX12B WNT11 CD36 GREM1 ROR2 SH3RF3 TBX1 NGF S100A12
Negative regulation of nucleobasE−containing compound metabolic process	22	1575	4.15E−03	ZBTB16 BMP4 SIX2 OVOL1 SOX11 ZNF154 ID3 CREB3L1 CDA ELF3 ZC3H12A HEYL PITX1 NOTCH3 CD36 GREM1 NRARP USP2 WNT11 GLIS3 MDFI TWIST2
Regionalization	9	355	4.24E−03	BMP4 ZBTB16 GREM1 ROR2 SIX2 NRARP MAFB MDFI TBX1
Regulation of protein import into nucleus	4	58	4.41E−03	BMP4 ZC3H12A JUP CD36
Negative regulation of macromolecule biosynthetic process	22	1597	4.86E−03	ZBTB16 BMP4 SIX2 OVOL1 SOX11 ZNF154 ID3 CREB3L1 ELF3 ZC3H12A HEYL PITX1 NOTCH3 SMPD3 CD36 GREM1 NRARP USP2 WNT11 GLIS3 MDFI TWIST2
Positive regulation of osteoblast differentiation	4	60	4.86E−03	BMP4 GDPD2 SOX11 HGF
Positive regulation of transcription, DNA-templated	22	1599	4.86E−03	DLX5 ZBTB16 HEYL JUP SOX11 BMP4 ELF3 ZC3H12A PITX1 NOTCH3 FGF7 CREB3L1 GREM1 ROR2 ADRB2 SIX2 OVOL1 MAFB WNT11 GLIS3 TBX1 HGF
Renal system vasculature development	3	26	4.86E−03	BMP4 NOTCH3 ANGPT2
Kidney vasculature development	3	26	4.86E−03	BMP4 NOTCH3 ANGPT2
Cell differentiation involved in metanephros development	3	26	4.86E−03	BMP4 GREM1 SIX2
Metanephric nephron morphogenesis	3	26	4.86E−03	BMP4 GREM1 SIX2
Glycosaminoglycan metabolic process	6	161	4.99E−03	HGF SMPD3 SDC1 HS3ST3A1 B3GNT4 B3GNT3
Regulation of protein import	4	61	5.07E−03	BMP4 ZC3H12A JUP CD36
Branching involved in ureteric bud morphogenesis	4	61	5.07E−03	BMP4 GREB1L GREM1 SIX2
Glomerulus development	4	61	5.07E−03	BMP4 NOTCH3 ANGPT2 HEYL
Negative regulation of cartilage development	3	27	5.28E−03	BMP4 GREM1 WNT11
Regulation of mesonephros development	3	27	5.28E−03	BMP4 GREM1 SIX2
Bone development	7	228	5.49E−03	MYOC SMPD3 DLX5 BMP4 FGF18 PAPSS2 GREM1
Negative regulation of cellular macromolecule biosynthetic process	21	1511	5.53E−03	ZBTB16 BMP4 SIX2 OVOL1 SOX11 ZNF154 ID3 CREB3L1 ELF3 ZC3H12A HEYL PITX1 NOTCH3 CD36 GREM1 NRARP USP2 WNT11 GLIS3 MDFI TWIST2
Regulation of angiogenesis	8	298	5.53E−03	ZC3H12A HGF ANGPT2 GREM1 CCBE1 FGF18 CREB3L1 JUP
Peptide cross-linking	4	63	5.57E−03	SPRR2D SPRR2A TGM1 PI3
Embryonic hindlimb morphogenesis	3	28	5.73E−03	PITX1 ZBTB16 BMP4
Secondary palate development	3	28	5.73E−03	SOX11 WNT11 TBX1
Glycosaminoglycan biosynthetic process	5	111	5.78E−03	SMPD3 SDC1 HS3ST3A1 B3GNT4 B3GNT3
Artery morphogenesis	4	64	5.81E−03	BMP4 NOTCH3 WNT11 TBX1
Regulation of localization	33	2905	5.84E−03	HGF RHOD MYOC ANGPT2 BMP4 CYP19A1 FGF7 FGF18 ZC3H12A ROR2 JUP FAM3D BDKRB1 CHRNA4 SMPD3 ALOX12 CD36 PKDCC AVPR1A GREM1 ADRB2 CLIC3 SOX11 CCBE1 KCNK12 DUOX1 TWIST2 WNT11 SDC1 G6PC2 ALOX12B SIK1 GLP1R
Cardiac septum development	5	112	5.93E−03	HEYL WNT11 BMP4 SOX11 TBX1
Positive regulation of cellular biosynthetic process	26	2082	5.95E−03	DLX5 ZBTB16 HEYL JUP SOX11 HGF BMP4 ELF3 ZC3H12A GREM1 PITX1 NOTCH3 KRT17 CD36 FGF7 CREB3L1 AVPR1A ROR2 ADRB2 SIX2 OVOL1 MAFB WNT11 GLIS3 TBX1 SMPD3
Response to lipid	16	1006	5.95E−03	IL36G IL1RN ZC3H12A HEYL BDKRB1 ALOX12 SDC1 ID3 SLPI CD36 AVPR1A CLDN4 BMP4 WNT11 TBX1 CATSPERB
MAPK cascade	16	1007	5.97E−03	BMP4 IL36G IL1RN MYOC FGF18 ZC3H12A ADRB2 ALOX12B HGF CD36 ROR2 SH3RF3 TBX1 NGF FGF7 S100A12
Negative regulation of cell adhesion	8	304	5.99E−03	MYOC IL1RN AJAP1 ANGPT2 ALOX12 ZC3H12A NRARP BMP4
Renal system vasculature morphogenesis	2	7	6.07E−03	NOTCH3 BMP4
Kidney vasculature morphogenesis	2	7	6.07E−03	NOTCH3 BMP4
Positive regulation of phosphorylation	17	1111	6.10E−03	HGF BMP4 IL36G IL1RN FGF7 FGF18 ZC3H12A ADRB2 ALOX12B WNT11 CD36 GREM1 ROR2 SH3RF3 TBX1 NGF S100A12
Regulation of cell proliferation	23	1756	6.32E−03	NRARP BMP4 FGF7 GREM1 JUP NOTCH3 WNT11 SMPD3 DLX5 ALOX12 ZBTB16 RARRES1 FGF18 AVPR1A SIX2 OVOL1 SOX11 SULT2B1 TGM1 ROR2 TBX1 NGF ADRA1D
Negative regulation of nitrogen compound metabolic process	30	2564	6.32E−03	ZBTB16 BMP4 SIX2 OVOL1 SOX11 ZNF154 HGF ID3 CREB3L1 CDA ELF3 ZC3H12A HEYL GREM1 PITX1 NOTCH3 BDKRB1 SMPD3 PI3 SLPI NGF CD36 NRARP SPINK9 COL28A1 USP2 WNT11 GLIS3 MDFI TWIST2
Cardiac chamber development	6	172	6.32E−03	HEYL WNT11 BMP4 GREB1L SOX11 TBX1
Negative regulation of developmental process	16	1017	6.40E−03	ID3 BMP4 MAFB TWIST2 NOTCH3 ANGPT2 ZBTB16 ZC3H12A ADRB2 SIX2 SOX11 NRARP CREB3L1 GREM1 TBX1 WNT11
Signal transduction by protein phosphorylation	16	1018	6.44E−03	BMP4 IL36G IL1RN MYOC FGF18 ZC3H12A ADRB2 ALOX12B HGF CD36 ROR2 SH3RF3 TBX1 NGF FGF7 S100A12
Transmembrane receptor protein tyrosine kinase signaling pathway	13	730	6.45E−03	HGF NGF ROR2 ANGPT2 CREB3L1 CCBE1 MYOC SMPD3 FGF7 FGF18 GREM1 CHN1 ADRB2
Cell adhesion	21	1541	6.46E−03	LYPD3 LYPD5 MYOC CD36 IL1RN S100A9 JUP AJAP1 ANGPT2 ALOX12 ZBTB16 COL8A1 FBLIM1 ZC3H12A GREM1 RHOD NRARP COL28A1 BMP4 CLDN4 LRRN2
Epithelial cell proliferation	9	387	6.56E−03	NRARP BMP4 FGF7 HGF DLX5 COL8A1 SOX11 TGM1 TBX1
Biological adhesion	21	1548	6.78E−03	LYPD3 LYPD5 MYOC CD36 IL1RN S100A9 JUP AJAP1 ANGPT2 ALOX12 ZBTB16 COL8A1 FBLIM1 ZC3H12A GREM1 RHOD NRARP COL28A1 BMP4 CLDN4 LRRN2
Myeloid leukocyte migration	7	241	6.79E−03	IL36G IL1RN S100A9 CYP19A1 GREM1 ROR2 S100A12
Positive regulation of biosynthetic process	26	2113	6.91E−03	DLX5 ZBTB16 HEYL JUP SOX11 HGF BMP4 CREB3L1 ELF3 ZC3H12A GREM1 PITX1 NOTCH3 KRT17 CD36 FGF7 AVPR1A ROR2 ADRB2 SIX2 OVOL1 MAFB WNT11 GLIS3 TBX1 SMPD3
Blood vessel endothelial cell migration	5	119	7.19E−03	ANGPT2 ALOX12 GREM1 FGF18 JUP
Mesenchymal cell differentiation involved in renal system development	2	8	7.48E−03	BMP4 SIX2
Mesenchymal-epithelial cell signaling	2	8	7.48E−03	HGF FGF7
Glomerulus morphogenesis	2	8	7.48E−03	NOTCH3 BMP4
Mesenchymal cell differentiation involved in kidney development	2	8	7.48E−03	BMP4 SIX2
Negative regulation of mesenchymal cell proliferation	2	8	7.48E−03	BMP4 WNT11
Negative regulation of cellular metabolic process	31	2724	7.59E−03	ZBTB16 BMP4 SIX2 OVOL1 SOX11 ZNF154 HGF ID3 CREB3L1 CDA ELF3 ZC3H12A HEYL GREM1 PITX1 NOTCH3 BDKRB1 SMPD3 PI3 SLPI NGF CD36 NRARP SPINK9 COL28A1 USP2 WNT11 GLIS3 MDFI SIK1 TWIST2
Multicellular organismal water homeostasis	4	71	7.63E−03	ALOX12 TMEM79 ALOX12B CLDN4
Endochondral bone morphogenesis	4	72	8.01E−03	SMPD3 DLX5 BMP4 FGF18
Metanephros morphogenesis	3	33	8.15E−03	BMP4 GREM1 SIX2
Positive regulation of nucleic acid-templated transcription	22	1694	8.53E−03	DLX5 ZBTB16 HEYL JUP SOX11 BMP4 ELF3 ZC3H12A PITX1 NOTCH3 FGF7 CREB3L1 GREM1 ROR2 ADRB2 SIX2 OVOL1 MAFB WNT11 GLIS3 TBX1 HGF
Positive regulation of RNA biosynthetic process	22	1695	8.56E−03	DLX5 ZBTB16 HEYL JUP SOX11 BMP4 ELF3 ZC3H12A PITX1 NOTCH3 FGF7 CREB3L1 GREM1 ROR2 ADRB2 SIX2 OVOL1 MAFB WNT11 GLIS3 TBX1 HGF
Positive regulation of morphogenesis of an epithelium	3	34	8.72E−03	ALOX12 BMP4 GREM1
Regulation of mesenchymal cell proliferation	3	34	8.72E−03	BMP4 WNT11 TBX1
Ventricular septum development	4	74	8.72E−03	HEYL WNT11 BMP4 SOX11
Ear morphogenesis	5	127	9.06E−03	DLX5 ROR2 SIX2 MAFB TBX1
Cell proliferation	26	2165	9.07E−03	NRARP DLX5 BMP4 FGF7 GREM1 SIX2 JUP HGF NOTCH3 WNT11 SMPD3 ALOX12 ZBTB16 RARRES1 COL8A1 FGF18 AVPR1A OVOL1 SOX11 SULT2B1 TGM1 SDCBP2 ROR2 TBX1 NGF ADRA1D
Soft palate development	2	9	9.07E−03	SOX11 TBX1
Regulation of branching involved in salivary gland morphogenesis	2	9	9.07E−03	HGF FGF7
Mesonephric duct development	2	9	9.07E−03	GREB1L WNT11
Regulation of DNA-binding transcription factor activity	10	495	9.09E−03	ID3 ZC3H12A HEYL JUP GREM1 CD36 SIK1 MDFI S100A9 S100A12
Regulation of hormone levels	11	583	9.12E−03	CYP19A1 FAM3D SMPD3 IL1RN DUOX1 CRABP1 SOX11 CHST8 G6PC2 GLP1R NGF
Water homeostasis	4	76	9.28E−03	ALOX12 TMEM79 ALOX12B CLDN4
Cellular response to toxic substance	7	259	9.32E−03	HGF SMPD3 CD36 DUOX1 S100A9 ZC3H12A GPX3
Cardiac chamber morphogenesis	5	129	9.38E−03	HEYL WNT11 BMP4 SOX11 TBX1
Cardiac ventricle development	5	130	9.67E−03	HEYL WNT11 BMP4 GREB1L SOX11
Regulation of signal transduction	37	3529	9.74E−03	BMP4 IL36G IL1RN SIK1 RSPO3 NRARP JUP HGF MYOC KLK14 FGF18 CREB3L1 S100A9 S100A12 ZC3H12A HEYL GREM1 ADRB2 ALOX12B LYNX1 CCBE1 WNT11 DLX5 RASL11B CHN1 NGF CD36 FGF7 ROR2 SOX11 FAM3D SH3RF3 MDFI TBX1 NOTCH3 ELF3 RHOD
Hindlimb morphogenesis	3	36	9.82E−03	PITX1 ZBTB16 BMP4
Odontogenesis	5	131	9.89E−03	SMPD3 SDC1 ID3 BMP4 TBX1
Regulation of bone mineralization	4	78	9.90E−03	BMP4 PKDCC ADRB2 GREM1
Polysaccharide biosynthetic process	4	78	9.90E−03	B3GNT4 B3GNT8 B3GNT3 SMPD3
Negative regulation of multicellular organismal process	18	1286	9.90E−03	ID3 ADRB2 ANGPT2 BMP4 ZC3H12A MAFB TWIST2 HGF NOTCH3 WNT11 ALOX12 ZBTB16 AVPR1A SOX11 NRARP CREB3L1 JUP GREM1
Regulation of protein phosphorylation	20	1503	9.92E−03	HGF BMP4 IL36G IL1RN MYOC FGF7 FGF18 ZC3H12A GREM1 ADRB2 ALOX12B WNT11 BDKRB1 SMPD3 CD36 ROR2 SH3RF3 TBX1 NGF S100A12
Cell surface receptor signaling pathway	35	3287	1.00E−02	HGF WNT11 BMP4 NGF IL36G IL1RN RSPO3 HEYL ROR2 NRARP JUP ANGPT2 DUOX1 CREB3L1 GREM1 CCBE1 FAM83G MYOC NOTCH3 CHRNA4 SMPD3 DLX5 GLP1R SDC1 RASL11B CD36 FGF7 FGF18 ZC3H12A ADRB2 SOX11 MDFI CHN1 CDA ELF3
Positive regulation of phosphorus metabolic process	17	1183	1.00E−02	HGF BMP4 IL36G IL1RN FGF7 FGF18 ZC3H12A ADRB2 ALOX12B WNT11 CD36 GREM1 ROR2 SH3RF3 TBX1 NGF S100A12
Positive regulation of phosphate metabolic process	17	1183	1.00E−02	HGF BMP4 IL36G IL1RN FGF7 FGF18 ZC3H12A ADRB2 ALOX12B WNT11 CD36 GREM1 ROR2 SH3RF3 TBX1 NGF S100A12
Cellular response to oxidised low-density lipoprotein particle stimulus	2	10	1.05E−02	SMPD3 CD36
Positive regulation of keratinocyte proliferation	2	10	1.05E−02	FGF7 TGM1
Positive regulation of mitochondrial depolarization	2	10	1.05E−02	MYOC ALOX12
Epithelial cell proliferation involved in lung morphogenesis	2	10	1.05E−02	BMP4 FGF7
Epithelial tube morphogenesis	8	344	1.06E−02	NRARP BMP4 ALOX12 GREB1L GREM1 SIX2 SOX11 WNT11
Epithelial to mesenchymal transition	5	135	1.08E−02	WNT11 BMP4 HEYL GREM1 HGF
Negative regulation of growth	7	269	1.08E−02	BMP4 CDA BDKRB1 CD36 GREM1 ADRB2 WNT11
Mesoderm development	5	136	1.11E−02	BMP4 WNT11 SIX2 OVOL1 TBX1
Canonical Wnt signaling pathway	8	350	1.16E−02	RSPO3 NRARP JUP GREM1 WNT11 DLX5 SDC1 ROR2
Digestive tract development	5	139	1.21E−02	BMP4 PKDCC SIX2 SOX11 WNT11
Mesenchymal cell development	4	84	1.23E−02	BMP4 HEYL SOX11 TBX1
Positive regulation of bone mineralization	3	40	1.24E−02	BMP4 PKDCC ADRB2
Embryonic digestive tract development	3	40	1.24E−02	PKDCC SIX2 SOX11
Regulation of cellular response to growth factor stimulus	7	277	1.24E−02	FGF18 CREB3L1 GREM1 CCBE1 BMP4 RASL11B SOX11
Cell fate commitment	7	277	1.24E−02	WNT11 BMP4 PITX1 NOTCH3 ROR2 SIX2 TBX1
Glycoprotein biosynthetic process	8	357	1.28E−02	HS3ST3A1 B3GNT4 FUT2 B3GNT8 B3GNT3 CHST8 ST8SIA2 ST6GAL2
Transmembrane receptor protein serine/threonine kinase signaling pathway	8	358	1.30E−02	BMP4 GREM1 FAM83G SMPD3 DLX5 RASL11B ROR2 SOX11
Axis specification	4	86	1.30E−02	BMP4 SIX2 NRARP MDFI
Negative regulation of T cell differentiation	3	41	1.30E−02	ZC3H12A NRARP BMP4
Positive regulation of nitrogen compound metabolic process	35	3351	1.30E−02	HGF DLX5 ZBTB16 BMP4 IL36G IL1RN HEYL JUP SOX11 FGF7 FGF18 S100A9 ELF3 ZC3H12A GREM1 ADRB2 ALOX12B CCBE1 PITX1 NOTCH3 WNT11 ALOX12 KRT17 CD36 CREB3L1 ROR2 SIX2 OVOL1 MAFB SH3RF3 GLIS3 TBX1 SMPD3 NGF S100A12
Metanephric nephron development	3	41	1.30E−02	BMP4 GREM1 SIX2
Negative regulation of cell communication	19	1440	1.31E−02	SIK1 NRARP HGF MYOC BMP4 KLK14 IL1RN CREB3L1 HEYL GREM1 ADRB2 FAM3D WNT11 RASL11B ZC3H12A AVPR1A ROR2 MDFI NOTCH3
Positive regulation of cellular metabolic process	36	3482	1.31E−02	HGF DLX5 ZBTB16 BMP4 IL36G IL1RN HEYL JUP SOX11 FGF7 FGF18 S100A9 ELF3 ZC3H12A GREM1 ADRB2 ALOX12B CCBE1 PITX1 NOTCH3 WNT11 ALOX12 KRT17 CD36 CREB3L1 AVPR1A ROR2 SIX2 OVOL1 MAFB SH3RF3 GLIS3 TBX1 SMPD3 NGF S100A12
Negative regulation of signaling	19	1444	1.35E−02	SIK1 NRARP HGF MYOC BMP4 KLK14 IL1RN CREB3L1 HEYL GREM1 ADRB2 FAM3D WNT11 RASL11B ZC3H12A AVPR1A ROR2 MDFI NOTCH3
Antimicrobial humoral response	5	145	1.38E−02	SLPI S100A9 S100A12 DEFB4A PI3
Hair follicle development	4	88	1.38E−02	KRT17 FGF7 TMEM79 KRT25
Regulation of epidermis development	4	88	1.38E−02	BMP4 KRT17 TMEM79 SULT2B1
Artery development	4	88	1.38E−02	BMP4 NOTCH3 WNT11 TBX1
Positive regulation of cell proliferation	15	1022	1.39E−02	NRARP BMP4 FGF7 GREM1 NOTCH3 SMPD3 DLX5 ALOX12 FGF18 AVPR1A SIX2 SOX11 TGM1 TBX1 ADRA1D
Positive regulation of response to stimulus	29	2621	1.39E−02	BMP4 IL36G IL1RN RSPO3 NRARP JUP HGF MYOC KLK14 FGF18 S100A9 S100A12 ZC3H12A ADRB2 ALOX12B CCBE1 DLX5 CHN1 CD36 GREM1 ROR2 SOX11 DUOX1 WNT11 SH3RF3 NGF TBX1 FGF7 ELF3
Positive regulation of RNA metabolic process	22	1789	1.39E−02	DLX5 ZBTB16 HEYL JUP SOX11 BMP4 ELF3 ZC3H12A PITX1 NOTCH3 FGF7 CREB3L1 GREM1 ROR2 ADRB2 SIX2 OVOL1 MAFB WNT11 GLIS3 TBX1 HGF
Positive regulation of membrane depolarization	2	12	1.39E−02	MYOC ALOX12
Regulation of intracellular signal transduction	24	2027	1.42E−02	BMP4 IL36G IL1RN SIK1 HGF MYOC FGF18 S100A9 S100A12 ZC3H12A ADRB2 ALOX12B CHN1 CD36 CREB3L1 GREM1 ROR2 SOX11 WNT11 SH3RF3 NGF TBX1 FGF7 RHOD
Positive regulation of cellular protein metabolic process	21	1680	1.42E−02	HGF BMP4 IL36G IL1RN FGF7 FGF18 S100A9 ZC3H12A ADRB2 ALOX12B CCBE1 WNT11 ALOX12 KRT17 CD36 GREM1 ROR2 SH3RF3 TBX1 NGF S100A12
Lung alveolus development	3	43	1.42E−02	BMP4 SMPD3 PKDCC
Pattern specification process	9	451	1.42E−02	BMP4 ZBTB16 GREM1 ROR2 SIX2 NRARP MAFB MDFI TBX1
Growth	15	1028	1.44E−02	NGF BMP4 FGF7 CDA WNT11 BDKRB1 SMPD3 KRT17 CD36 CYP19A1 PKDCC AVPR1A GREM1 ADRB2 S100A9
Molting cycle process	4	90	1.44E−02	KRT17 FGF7 TMEM79 KRT25
Hair cycle process	4	90	1.44E−02	KRT17 FGF7 TMEM79 KRT25
Wnt signaling pathway	10	543	1.48E−02	WNT11 RSPO3 NRARP JUP GREM1 DLX5 SDC1 ROR2 MYOC MDFI
Negative regulation of osteoblast differentiation	3	44	1.49E−02	TWIST2 ID3 GREM1
Skin epidermis development	4	91	1.49E−02	KRT17 FGF7 TMEM79 KRT25
Regulation of Wnt signaling pathway	8	371	1.49E−02	RSPO3 NRARP JUP GREM1 WNT11 DLX5 ROR2 MDFI
Cell–cell signaling by wnt	10	545	1.50E−02	WNT11 RSPO3 NRARP JUP GREM1 DLX5 SDC1 ROR2 MYOC MDFI
Digestive system development	5	151	1.54E−02	BMP4 PKDCC SIX2 SOX11 WNT11
Insemination	2	13	1.57E−02	KLK14 AVPR1A
Extracellular structure organization	9	460	1.57E−02	COL8A1 COL28A1 SMPD3 CD36 ADAMTSL4 ELF3 SDC1 GREM1 CREB3L1
Protein import into nucleus	5	152	1.57E−02	BMP4 ZC3H12A JUP CD36 SIX2
Positive regulation of cell death	12	741	1.61E−02	BMP4 ADAMTSL4 S100A9 ZC3H12A WNT11 ALOX12 ZBTB16 ID3 CD36 SIK1 PNMA5 NGF
Muscle structure development	11	645	1.61E−02	BMP4 USP2 PITX1 SDC1 ID3 HEYL AVPR1A GREM1 SOX11 SIK1 TBX1
Anterior/posterior pattern specification	6	222	1.63E−02	BMP4 ZBTB16 ROR2 SIX2 NRARP TBX1
Epithelial cell differentiation involved in kidney development	3	46	1.63E−02	BMP4 GREM1 SIX2
Regulation of canonical Wnt signaling pathway	7	298	1.65E−02	RSPO3 NRARP JUP GREM1 WNT11 DLX5 ROR2
Regulation of blood vessel endothelial cell migration	4	95	1.68E−02	ANGPT2 ALOX12 FGF18 JUP
Secretion by cell	21	1715	1.70E−02	CYP19A1 FGF7 FAM3D CHRNA4 SMPD3 CD36 IL1RN TMEM79 ZC3H12A AVPR1A SOX11 SDC1 G6PC2 CREB3L1 HGF GLP1R SLPI CDA S100A9 S100A12 JUP
Gland development	9	468	1.71E−02	HGF PITX1 BMP4 CYP19A1 FGF7 ELF3 MAFB WNT11 TBX1
Embryonic cranial skeleton morphogenesis	3	47	1.71E−02	BMP4 SIX2 TBX1
Regulation of biomineral tissue development	4	97	1.78E−02	BMP4 PKDCC ADRB2 GREM1
Mating	3	48	1.80E−02	KLK14 PI3 AVPR1A
Response to external stimulus	28	2561	1.80E−02	IL36G IL1RN SIK1 DEFB4A HGF ANGPT2 SLPI CD36 FGF7 FGF18 S100A9 S100A12 ZC3H12A JUP AJAP1 USP2 WNT11 BDKRB1 DLX5 ALOX12 BMP4 CHN1 CYP19A1 AVPR1A GREM1 ADRB2 PI3 LTB4R2
Positive regulation of canonical Wnt signaling pathway	5	159	1.83E−02	RSPO3 NRARP JUP DLX5 ROR2
Positive regulation of protein modification process	17	1279	1.83E−02	HGF BMP4 IL36G IL1RN FGF7 FGF18 ZC3H12A ADRB2 ALOX12B WNT11 CD36 GREM1 ROR2 SH3RF3 TBX1 NGF S100A12
Somite development	4	98	1.83E−02	WNT11 ROR2 SOX11 NRARP
Regulation of binding	8	388	1.83E−02	ID3 SLPI BMP4 ADRB2 SOX11 CD36 MDFI NGF
Genitalia development	3	49	1.87E−02	CYP19A1 GREB1L ROR2
Positive regulation of biomineral tissue development	3	49	1.87E−02	BMP4 PKDCC ADRB2
Ear development	6	232	1.93E−02	DLX5 BMP4 ROR2 SIX2 MAFB TBX1
Branching morphogenesis of an epithelial tube	5	162	1.94E−02	NRARP BMP4 GREB1L GREM1 SIX2
Positive regulation of exosomal secretion	2	15	1.95E−02	SDC1 SMPD3
Regulation of transcription by RNA polymerase II	30	2830	1.95E−02	DLX5 ZBTB16 BMP4 ELF3 HEYL JUP SOX11 ZC3H12A GREM1 FAM83G PITX1 NOTCH3 SMPD3 GLIS3 ID3 TBX4 CD36 CREB3L1 ROR2 ADRB2 SIX2 OVOL1 ZNF154 NRARP MAFB ZNF469 USP2 MDFI TBX1 HGF
Activation of transmembrane receptor protein tyrosine kinase activity	2	15	1.95E−02	GREM1 ADRB2
Positive regulation of macromolecule biosynthetic process	23	1978	1.96E−02	DLX5 ZBTB16 HEYL JUP SOX11 HGF BMP4 ELF3 ZC3H12A GREM1 PITX1 NOTCH3 KRT17 FGF7 CREB3L1 ROR2 ADRB2 SIX2 OVOL1 MAFB WNT11 GLIS3 TBX1
Secretion	22	1861	1.96E−02	CYP19A1 FGF7 FAM3D CHRNA4 SMPD3 CD36 IL1RN TMEM79 ZC3H12A AVPR1A SOX11 SDC1 G6PC2 ALOX12B CREB3L1 HGF GLP1R SLPI CDA S100A9 S100A12 JUP
Positive regulation of smooth muscle cell proliferation	4	101	1.97E−02	BMP4 NOTCH3 SMPD3 ALOX12
Negative regulation of hemopoiesis	5	164	2.00E−02	MAFB ZBTB16 ZC3H12A NRARP BMP4
Regulation of interleukin-6 production	5	164	2.00E−02	IL36G IL1RN ZC3H12A HGF CD36
Response to organic cyclic compound	14	975	2.02E−02	IL1RN DUOX1 HEYL JUP ANGPT2 SLC8A3 SDC1 ID3 CD36 ZC3H12A AVPR1A CLDN4 BMP4 CATSPERB
Intermediate filament cytoskeleton organization	3	51	2.02E−02	TCHH KRT17 KRT25
Negative regulation of lymphocyte differentiation	3	51	2.02E−02	ZC3H12A NRARP BMP4
Negative regulation of cell differentiation	12	774	2.08E−02	ID3 BMP4 MAFB TWIST2 NOTCH3 ZBTB16 ZC3H12A SIX2 SOX11 NRARP GREM1 TBX1
Regulation of signaling receptor activity	11	676	2.08E−02	LYNX1 HGF BMP4 NGF IL36G IL1RN FGF7 FGF18 GREM1 FAM3D ADRB2
Negative regulation of transcription by RNA polymerase II	13	876	2.08E−02	BMP4 SOX11 ZBTB16 NOTCH3 ID3 CD36 CREB3L1 HEYL OVOL1 NRARP USP2 GLIS3 MDFI
Anterior/posterior axis specification	3	52	2.10E−02	BMP4 SIX2 NRARP
Positive regulation of cell–matrix adhesion	3	52	2.10E−02	MYOC CD36 JUP
Intermediate filament-based process	3	52	2.10E−02	TCHH KRT17 KRT25
Regulation of transcription from RNA polymerase II promoter involved in heart development	2	16	2.11E−02	BMP4 GREM1
Regulation of exosomal secretion	2	16	2.11E−02	SDC1 SMPD3
Cellular response to oxygen-containing compound	16	1199	2.13E−02	IL36G IL1RN HGF ZC3H12A JUP SLC8A3 CHRNA4 SMPD3 GLP1R ID3 CD36 AVPR1A ADRB2 WNT11 TBX1 GNA15
Positive regulation of apoptotic process	11	680	2.13E−02	BMP4 ADAMTSL4 S100A9 WNT11 ALOX12 ZBTB16 ID3 ZC3H12A SIK1 PNMA5 NGF
Negative regulation of response to stimulus	21	1764	2.13E−02	SIK1 NRARP HGF MYOC ANGPT2 BMP4 KLK14 IL1RN CREB3L1 HEYL GREM1 ADRB2 AJAP1 WNT11 ALOX12 RASL11B CYP19A1 ZC3H12A ROR2 MDFI NOTCH3
Negative regulation of binding	5	168	2.13E−02	ID3 SLPI ADRB2 SOX11 MDFI
Positive regulation of macromolecule metabolic process	35	3498	2.13E−02	HGF DLX5 ZBTB16 BMP4 IL36G IL1RN HEYL JUP SOX11 FGF7 FGF18 S100A9 ELF3 ZC3H12A GREM1 ADRB2 ALOX12B CCBE1 PITX1 NOTCH3 WNT11 ALOX12 KRT17 CD36 CREB3L1 ROR2 SIX2 OVOL1 MAFB NGF SH3RF3 GLIS3 ACTG2 TBX1 S100A12
Muscle organ development	8	404	2.15E−02	BMP4 USP2 PITX1 ID3 HEYL GREM1 SOX11 TBX1
Negative regulation of DNA binding	3	53	2.16E−02	ID3 SOX11 MDFI
Mesenchyme morphogenesis	3	53	2.16E−02	WNT11 HEYL ACTG2
Regulation of phosphorylation	20	1653	2.17E−02	HGF BMP4 IL36G IL1RN MYOC FGF7 FGF18 ZC3H12A GREM1 ADRB2 ALOX12B WNT11 BDKRB1 SMPD3 CD36 ROR2 SH3RF3 TBX1 NGF S100A12
Positive regulation of programmed cell death	11	686	2.23E−02	BMP4 ADAMTSL4 S100A9 WNT11 ALOX12 ZBTB16 ID3 ZC3H12A SIK1 PNMA5 NGF
Unsaturated fatty acid biosynthetic process	3	54	2.25E−02	ALOX12 AVPR1A ALOX12B
Inner ear morphogenesis	4	107	2.25E−02	DLX5 ROR2 MAFB TBX1
Regulation of striated muscle cell differentiation	4	107	2.25E−02	GREM1 BMP4 SIK1 TBX1
Negative regulation of signal transduction	17	1321	2.27E−02	SIK1 NRARP HGF MYOC BMP4 KLK14 IL1RN CREB3L1 HEYL GREM1 ADRB2 WNT11 RASL11B ZC3H12A ROR2 MDFI NOTCH3
Lipoxygenase pathway	2	17	2.27E−02	ALOX12 ALOX12B
Interleukin-6 production	5	172	2.27E−02	IL36G IL1RN ZC3H12A HGF CD36
Linoleic acid metabolic process	2	17	2.27E−02	ALOX12 ALOX12B
Pulmonary valve morphogenesis	2	17	2.27E−02	BMP4 HEYL
Negative regulation of molecular function	16	1216	2.33E−02	ID3 HGF SLPI BMP4 ZC3H12A HEYL ADRB2 PI3 NGF SOX11 LYNX1 SPINK9 COL28A1 MDFI SIK1 LTB4R2
Positive regulation of metabolic process	37	3789	2.34E−02	HGF DLX5 ZBTB16 BMP4 IL36G IL1RN HEYL JUP SOX11 FGF7 FGF18 CREB3L1 S100A9 ELF3 ZC3H12A GREM1 ADRB2 ALOX12B CCBE1 PITX1 NOTCH3 WNT11 ALOX12 KRT17 CD36 AVPR1A ROR2 SIX2 OVOL1 MAFB NGF SH3RF3 GLIS3 ACTG2 TBX1 SMPD3 S100A12
Segmentation	4	109	2.35E−02	BMP4 ROR2 NRARP MAFB
Regulation of stress-activated MAPK cascade	6	248	2.38E−02	IL36G IL1RN ZC3H12A HGF ROR2 SH3RF3
Cell differentiation involved in kidney development	3	56	2.42E−02	BMP4 GREM1 SIX2
Regulation of epithelial cell proliferation	7	330	2.43E−02	NRARP BMP4 FGF7 DLX5 SOX11 TGM1 TBX1
Import into nucleus	5	176	2.44E−02	BMP4 ZC3H12A JUP CD36 SIX2
Regulation of stress-activated protein kinase signaling cascade	6	250	2.44E−02	IL36G IL1RN ZC3H12A HGF ROR2 SH3RF3
Positive regulation of protein metabolic process	21	1796	2.45E−02	HGF BMP4 IL36G IL1RN FGF7 FGF18 S100A9 ZC3H12A ADRB2 ALOX12B CCBE1 WNT11 ALOX12 KRT17 CD36 GREM1 ROR2 SH3RF3 TBX1 NGF S100A12
Carbohydrate derivative biosynthetic process	12	800	2.45E−02	HS3ST3A1 B3GNT4 FUT2 B3GNT8 B3GNT3 PAPSS2 CHST8 ST8SIA2 SMPD3 ST6GAL2 SDC1 CDA
Regulation of nucleocytoplasmic transport	4	111	2.46E−02	BMP4 ZC3H12A JUP CD36
Embryonic digestive tract morphogenesis	2	18	2.47E−02	SIX2 SOX11
Regulation of kidney development	3	57	2.49E−02	BMP4 GREM1 SIX2
Negative regulation of leukocyte differentiation	4	112	2.52E−02	ZC3H12A NRARP MAFB BMP4
Lung morphogenesis	3	58	2.60E−02	BMP4 FGF7 SOX11
Leukocyte chemotaxis	6	255	2.62E−02	IL36G IL1RN S100A9 CYP19A1 GREM1 S100A12
Cell junction assembly	6	255	2.62E−02	MYOC JUP WNT11 GREM1 RHOD FBLIM1
Mucopolysaccharide metabolic process	4	114	2.65E−02	HGF SMPD3 B3GNT4 B3GNT3
Regulation of growth	11	710	2.67E−02	NGF BMP4 CDA BDKRB1 KRT17 CD36 AVPR1A GREM1 ADRB2 WNT11 S100A9
Regulation of production of miRNAs involved in gene silencing by miRNA	2	19	2.68E−02	BMP4 ZC3H12A
Exosomal secretion	2	19	2.68E−02	SDC1 SMPD3
Keratinocyte migration	2	19	2.68E−02	FGF7 LTB4R2
Kidney mesenchyme development	2	19	2.68E−02	BMP4 SIX2
Negative regulation of DNA-binding transcription factor activity	5	182	2.69E−02	ID3 ZC3H12A HEYL SIK1 MDFI
Bone morphogenesis	4	115	2.69E−02	SMPD3 DLX5 BMP4 FGF18
Polysaccharide metabolic process	4	116	2.76E−02	B3GNT4 B3GNT8 B3GNT3 SMPD3
Cellular oxidant detoxification	4	117	2.83E−02	CD36 DUOX1 S100A9 GPX3
Molting cycle	4	117	2.83E−02	KRT25 KRT17 FGF7 TMEM79
Hair cycle	4	117	2.83E−02	KRT25 KRT17 FGF7 TMEM79
Extracellular exosome biogenesis	2	20	2.84E−02	SDC1 SMPD3
Positive regulation of cardiac muscle cell differentiation	2	20	2.84E−02	GREM1 BMP4
Regulation of cell growth	8	432	2.84E−02	NGF CDA BDKRB1 KRT17 AVPR1A GREM1 WNT11 S100A9
Glycoprotein metabolic process	8	432	2.84E−02	HS3ST3A1 B3GNT4 FUT2 B3GNT8 B3GNT3 CHST8 ST8SIA2 ST6GAL2
Notochord development	2	20	2.84E−02	WNT11 ID3
Blood vessel endothelial cell proliferation involved in sprouting angiogenesis	2	20	2.84E−02	NRARP BMP4
Positive regulation of stress-activated MAPK cascade	5	186	2.84E−02	IL36G IL1RN ZC3H12A ROR2 SH3RF3
Response to hydroperoxide	2	20	2.84E−02	CD36 GPX3
Middle ear morphogenesis	2	20	2.84E−02	SIX2 TBX1
Entrainment of circadian clock by photoperiod	2	20	2.84E−02	USP2 SIK1
Negative regulation of leukocyte chemotaxis	2	20	2.84E−02	CYP19A1 GREM1
Regulation of production of small RNA involved in gene silencing by RNA	2	20	2.84E−02	BMP4 ZC3H12A
Hormone secretion	7	346	2.86E−02	CYP19A1 FAM3D SMPD3 IL1RN SOX11 G6PC2 GLP1R
Positive regulation of stress-activated protein kinase signaling cascade	5	187	2.88E−02	IL36G IL1RN ZC3H12A ROR2 SH3RF3
Negative regulation of endopeptidase activity	6	264	2.89E−02	HGF PI3 SLPI SPINK9 COL28A1 NGF
Neutrophil chemotaxis	4	119	2.89E−02	IL36G IL1RN S100A9 S100A12
Embryonic digit morphogenesis	3	62	2.91E−02	ZBTB16 BMP4 ROR2
Modification of morphology or physiology of other organism	5	188	2.91E−02	SLPI S100A9 S100A12 ZC3H12A DEFB4A
Positive regulation of cell-substrate adhesion	4	120	2.95E−02	MYOC CD36 COL8A1 JUP
Positive regulation of protein serine/threonine kinase activity	7	349	2.95E−02	BMP4 FGF18 HGF ROR2 ADRB2 NGF S100A12
Regulation of cell-substrate junction assembly	3	63	3.00E−02	MYOC GREM1 RHOD
Sialylation	2	21	3.00E−02	ST8SIA2 ST6GAL2
Regulation of protein localization to nucleus	4	121	3.00E−02	BMP4 ZC3H12A JUP CD36
Protein glycosylation	6	268	3.00E−02	B3GNT4 FUT2 B3GNT8 B3GNT3 ST8SIA2 ST6GAL2
Positive regulation of Wnt signaling pathway	5	190	3.00E−02	RSPO3 NRARP JUP DLX5 ROR2
Negative regulation of chondrocyte differentiation	2	21	3.00E−02	BMP4 GREM1
Macromolecule glycosylation	6	268	3.00E−02	B3GNT4 FUT2 B3GNT8 B3GNT3 ST8SIA2 ST6GAL2
Regulation of focal adhesion assembly	3	63	3.00E−02	MYOC GREM1 RHOD
Regulation of mitochondrial depolarization	2	21	3.00E−02	MYOC ALOX12
Pulmonary valve development	2	21	3.00E−02	BMP4 HEYL
Chordate embryonic development	10	632	3.02E−02	MDFI BMP4 RSPO3 ELF3 ROR2 SIX2 SOX11 NRARP WNT11 TBX1
O-glycan processing	3	64	3.07E−02	B3GNT4 B3GNT8 B3GNT3
Respiratory gaseous exchange	3	65	3.18E−02	CHRNA4 CCBE1 MAFB
Gland morphogenesis	4	124	3.18E−02	HGF BMP4 FGF7 ELF3
Negative regulation of chemotaxis	3	65	3.18E−02	ANGPT2 CYP19A1 GREM1
Positive regulation of striated muscle cell differentiation	3	65	3.18E−02	GREM1 BMP4 TBX1
Hormone transport	7	357	3.19E−02	CYP19A1 FAM3D SMPD3 IL1RN SOX11 G6PC2 GLP1R
Extracellular vesicle biogenesis	2	22	3.21E−02	SDC1 SMPD3
Positive regulation of transcription of Notch receptor target	2	22	3.21E−02	NOTCH3 HEYL
Gastrulation	5	195	3.21E−02	BMP4 IL1RN WNT11 SIX2 COL8A1
Negative regulation of peptidase activity	6	273	3.21E−02	HGF PI3 SLPI NGF SPINK9 COL28A1
Negative regulation of response to external stimulus	7	358	3.21E−02	ANGPT2 AJAP1 HGF ALOX12 CYP19A1 ZC3H12A GREM1
Proteolysis	22	1988	3.24E−02	CPXM2 HGF USP2 KLK14 S100A9 ZC3H12A CCBE1 KLK12 PRSS50 ALOX12 PI3 SLPI KLK10 NGF ADAMTSL4 PRSS35 ADAMTS15 KLK13 PRSS27 SPINK9 COL28A1 ADRB2
Negative regulation of cell-substrate adhesion	3	66	3.25E−02	MYOC AJAP1 ANGPT2
Transcription by RNA polymerase II	30	2992	3.25E−02	DLX5 ZBTB16 BMP4 ELF3 HEYL JUP SOX11 ZC3H12A GREM1 FAM83G PITX1 NOTCH3 SMPD3 GLIS3 ID3 TBX4 CD36 CREB3L1 ROR2 ADRB2 SIX2 OVOL1 ZNF154 NRARP MAFB ZNF469 USP2 MDFI TBX1 HGF
Regulation of phosphate metabolic process	21	1870	3.25E−02	HGF BMP4 IL36G IL1RN MYOC FGF7 FGF18 CDA ZC3H12A GREM1 ADRB2 ALOX12B WNT11 BDKRB1 SMPD3 CD36 ROR2 SH3RF3 TBX1 NGF S100A12
Regulation of membrane potential	8	450	3.25E−02	CHRNA4 KCNK12 MYOC SLC8A3 ALOX12 CD36 ADRB2 JUP
Positive regulation of MAP kinase activity	6	275	3.25E−02	BMP4 FGF18 HGF ROR2 NGF S100A12
Cellular detoxification	4	126	3.26E−02	CD36 DUOX1 S100A9 GPX3
Carbohydrate metabolic process	10	644	3.27E−02	G6PC2 B3GNT4 B3GNT8 B3GNT3 ST8SIA2 ST6GAL2 SMPD3 CHST8 FUT2 SIK1
Positive regulation of nucleobase-containing compound metabolic process	22	1993	3.27E−02	DLX5 ZBTB16 HEYL JUP SOX11 HGF BMP4 ELF3 ZC3H12A GREM1 PITX1 NOTCH3 FGF7 CREB3L1 ROR2 ADRB2 SIX2 OVOL1 MAFB WNT11 GLIS3 TBX1
Regulation of phosphorus metabolic process	21	1872	3.27E−02	HGF BMP4 IL36G IL1RN MYOC FGF7 FGF18 CDA ZC3H12A GREM1 ADRB2 ALOX12B WNT11 BDKRB1 SMPD3 CD36 ROR2 SH3RF3 TBX1 NGF S100A12
Protein localization to nucleus	6	276	3.27E−02	BMP4 ZC3H12A JUP ZBTB16 CD36 SIX2
Cofactor catabolic process	3	67	3.31E−02	GPX3 DUOX1 ALDH1L1
Male genitalia development	2	23	3.35E−02	GREB1L ROR2
Lens morphogenesis in camera-type eye	2	23	3.35E−02	BMP4 SOX11
Positive regulation of epidermal cell differentiation	2	23	3.35E−02	BMP4 SULT2B1
Negative regulation of muscle contraction	2	23	3.35E−02	ADRB2 ZC3H12A
Branching involved in salivary gland morphogenesis	2	23	3.35E−02	HGF FGF7
Endothelial tube morphogenesis	2	23	3.35E−02	BMP4 ALOX12
Morphogenesis of an endothelium	2	23	3.35E−02	BMP4 ALOX12
Cranial skeletal system development	3	68	3.39E−02	BMP4 SIX2 TBX1
Inflammatory response	12	856	3.39E−02	BDKRB1 LTB4R2 S100A9 HGF SDC1 IL36G IL1RN CYP19A1 S100A12 ELF3 ZC3H12A CD36
Negative regulation of cell–cell adhesion	5	200	3.39E−02	IL1RN ALOX12 ZC3H12A NRARP BMP4
Glycosylation	6	280	3.41E−02	B3GNT4 FUT2 B3GNT8 B3GNT3 ST8SIA2 ST6GAL2
Embryo development ending in birth or egg hatching	10	652	3.44E−02	MDFI BMP4 RSPO3 ELF3 ROR2 SIX2 SOX11 NRARP WNT11 TBX1
Regulation of adherens junction organization	3	69	3.48E−02	MYOC GREM1 RHOD
Skeletal muscle cell differentiation	3	69	3.48E−02	HEYL SOX11 TBX1
Ceramide biosynthetic process	3	69	3.48E−02	ST8SIA2 ALOX12B SMPD3
Response to toxic substance	9	555	3.49E−02	CHRNA4 HGF SMPD3 SDC1 CD36 DUOX1 S100A9 ZC3H12A GPX3
Photoperiodism	2	24	3.51E−02	USP2 SIK1
Positive regulation of lipid storage	2	24	3.51E−02	ZC3H12A CD36
Purine ribonucleoside bisphosphate metabolic process	2	24	3.51E−02	PAPSS2 SULT2B1
Myelination	4	131	3.51E−02	MALL HGF MYOC SLC8A3
Positive regulation of protein kinase activity	9	557	3.51E−02	BMP4 FGF18 HGF WNT11 GREM1 ROR2 ADRB2 NGF S100A12
3 -phosphoadenosine 5 -phosphosulfate metabolic process	2	24	3.51E−02	PAPSS2 SULT2B1
Mitochondrial depolarization	2	24	3.51E−02	MYOC ALOX12
Uterus development	2	24	3.51E−02	CYP19A1 GREB1L
Regulation of cellular protein metabolic process	28	2768	3.54E−02	HGF BMP4 IL36G IL1RN MYOC FGF7 FGF18 S100A9 ZC3H12A GREM1 ADRB2 ALOX12B CCBE1 WNT11 BDKRB1 SMPD3 ALOX12 PI3 SLPI KRT17 NGF CD36 ROR2 SPINK9 COL28A1 SH3RF3 TBX1 S100A12
Formation of primary germ layer	4	132	3.56E−02	BMP4 WNT11 SIX2 COL8A1
Endocrine system development	4	132	3.56E−02	BMP4 PITX1 WNT11 TBX1
Regulation of DNA binding	4	132	3.56E−02	ID3 SOX11 MDFI NGF
Striated muscle cell differentiation	6	285	3.56E−02	BMP4 AVPR1A GREM1 SDC1 SIK1 TBX1
Inner ear development	5	205	3.59E−02	DLX5 BMP4 ROR2 MAFB TBX1
Neutrophil migration	4	133	3.62E−02	IL36G IL1RN S100A9 S100A12

**Table 4:** Sensory neuron markers

CALCA
CHRNA3
DIO3
FAM19A
GRM3
GRP
KCNQ2
MDGA1
MMD2
NECAB2
NTRK1
NTRK2
NTRK3
ONECUT3
P2RX3
PCP4
POU4F3
POU6F2
PRDM12
PRDM8
RET
RUNX1
RUNX3
SCN10A
SCN11A
SCN9A
STRA6
SYT13
TAC1
TRPA1
TRPM3
TRPM8
TRPV1
VIP

**Table 5:** Angiogenesis-related genes down-regulated in alive x dead

Gene.symbol	log2FC	FDR
ANGPT2	− 1.42767	3.23E−02
BMP4	− 1.23615	3.99E−02
CCBE1	− 1.04094	4.44E−02
COL8A1	− 1.22669	4.09E−02
CREB3L1	− 1.04632	4.44E−02
FGF18	− 1.35348	3.80E−03
GREM1	− 1.36182	1.10E−02
HGF	− 1.19158	4.28E−03
JUP	− 1.81687	1.10E−02
NOTCH3	− 1.22297	3.57E−03
NRARP	− 1.42862	5.97E−03
RSPO3	− 1.48997	6.13E−04
TBX1	− 1.26122	3.69E−02
TBX4	− 1.84817	5.39E−03
ZC3H12A	− 1.35742	4.67E−03

## Discussion

**Fig. 9:**
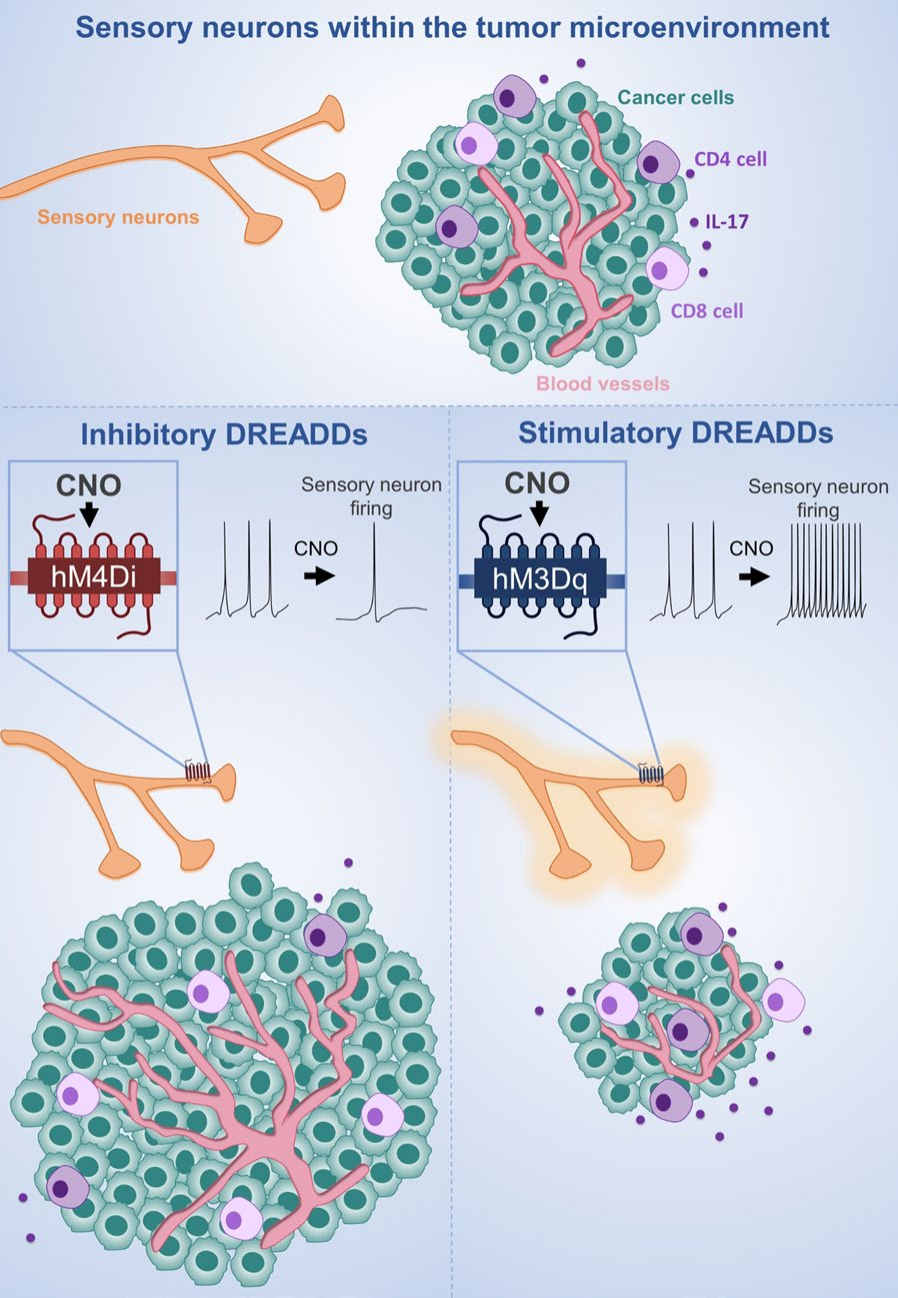
Schematic illustration summarizing the results of sensory neurons’ activity inhibition and overactivation in the melanoma microenvironment

In the present study, we examined how melanoma progression is affected by sensory neurons activity. Our chemogenetic approach revealed that inhibition of sensory activity promotes tumor growth and intra-tumoral angiogenesis. In contrast, excitation of sensory neurons induces melanoma regression with decrease in tumor growth and in new blood vessel formation, as well as a boost in the anti-tumor immune surveillance (Fig. 9). This work indicates that induction of hyperactivation in Nav1.8-expressing sensory neurons represents a potential new therapeutic path in the battle against melanoma.

Just as the role of particular genes in a specific biological process can be examined by evaluating examining the assessable consequences that result from their removal (e.g. using knockout animals), the role of neurons in the tumor microenvironment was previously assessed by eliminating them [32, 36, 71, 89, 108, 115, 116, 121, 154, 158]. Nevertheless, although biomedical research has gained some insights into the function of intra-tumoral neurons using loss-of-function studies with surgical or pharmacologic denervation, these strategies are mostly not specific to a given neuronal type. Importantly, a disadvantage of all these studies is that neuronal killing may generate secondary undesirable indirect side effects caused by the inflammatory tissular response which may influence the observed phenotypes (Männ et al. 2016; Christiaansen, Boggiatto, and Varga 2014; Bennett et al. 2005). To circumvent these limitations, novel powerful technologies have been created in the field of modern neuroscience which allow to manipulate the firing of specific neurons without killing them: optogenetics and chemogenetics. These methods use genetic strategies to deliver the expression of light-sensitive proteins or designer receptors exclusively activated by designer drugs, respectively, to the membrane of defined neuronal populations. Therefore, by using these techniques, manipulation of neurons by exposure to light or to designer drugs, without killing them, became feasible. As melanoma is a chronic disease, the use of optogenetics for longer periods, may not be the best approach, as the unavoidable surgical preparation with the chronic implantation of hardware for stimulation and prolonged exposure to highly energetic laser light will eventually culminate in confounding regional inflammation and tissue degradation. Therefore, we chose to use chemogenetics to examine the participation of sensory neurons in melanoma development, as it is more suitable to evaluate the long-term effects of sensory stimulations with less side-effects. Future studies may use similar approaches to explore the role of sensory neurons and other innervations in other cancers.

Our findings suggest that sensory neurons’ overactivation affects the immune response to melanoma. Melanoma progression is influenced by the complex interplay between cancer cells and different components of the immune system [8]. Melanoma cells may cause disruption of the organism’s immunity to overrun and escape the immune system control [96, 104]. The role of sensory innervations in these interactions remains completely unknown. Lymphocytes are the dominant immune elements found infiltrating the melanoma microenvironment. Their composition correlates with patients’ survival [38]. While regulatory T cells play pro-tumorigenic roles; CD8 + T cells, CD4 + T cells, γδ T cells, and NK cells have been shown to act against the transformed cells, [38, 43, 46, 47, 58, 64, 73, 118]. Conversely neutrophils and myeloid-derived suppressor cells have been associated with poor prognosis and are largely pro-tumorigenic [22, 28, 69, 126, 129, 145, 155]. Our data shows that sensory overactivation induce an increase in the number of tumor-infiltrating anti-cancer lymphocytes (CD8 + T cells, CD4 + T cells, γδ T cells, and NK cells), while we did not detect changes in the number of tumor-infiltrating regulatory T cells. We also found that there is a decrease in the number of neutrophils and myeloid-derived suppressor cells within the tumor. We found that these changes were tumor-specific, as we did not detect any alterations in the number of lymphocytes in the non-draining lymph nodes. Tumor-draining lymph nodes presented an increase in some of the anti-tumor lymphocytes probably because of the tumor-priming effect previously reported [135]. Signals transmitted to T cells via PD-1 or CTLA-4 (considered markers for T cells “exhaustion”) promote T cell dysfunction, thereby turning off the immune response [59, 98, 149]. We found that the tumor-infiltrating lymphocytes decrease their expression of PD1 and CTLA-4, possibly indicating that these cells are “less exhausted” within the melanoma microenvironment after sensory hyperactivation. The more active phenotypes of lymphocytes have been associated to the increase in the production of cytokines. A variety of lymphocytes are capable of producing IL-17 [14, 20, 24, 53, 56, 77, 94, 103] which has presented anti-tumorigenic effects in melanoma [3, 63, 74, 75, 91, 99, 100]. We found an increase in tumor-infiltrating lymphocytes producing IL-17 after sensory stimulation. Thus, in light of our overall findings, we suggest that induced increase in firing of sensory innervations contributes to boosting of the immune response against melanoma. Future studies will need to explore the exact molecular mechanisms involved in the interactions of sensory neurons and immune cells in the melanoma microenvironment.

A variety of cellular and molecular mechanisms may be involved in the effect of sensory neurons’ modulation on melanoma behavior. For instance, it has been documented that the same drug that is used to denervate sensory neurons, resiniferatoxin (RTX), an analogue of capsaicin, also induces stress by causing hyperactivation of the sympathetic nervous system [16, 62, 157]. These studies also revealed that sensory nerves may tune down sympathetic nerve activity [16, 62, 105]. Sympathetic neurons release norepinephrine [70, 123], which has been shown to strongly induce tumorigenesis [1, 60, 154]. It remains open the important question whether the effect of sensory innervations in the tumor microenvironment depend also on the modulation of the sympathetic tone.

## Future perspectives

The present study reveals the short-term impact of chemogenetic modulation of sensory neurons on melanoma behavior. It remains to be examined what are the long-term effects of this manipulations. In the current study, the sensory neurons’ activity is being continuously inhibited or overactivated. Are changes in sensory neurons’ activity at specific time points sufficient to influence cancer outcomes? Also, it remains to be determined what are the changes within the tumor microenvironment at different stages of cancer progression. Are some stages more sensible to changes in the activity of sensory neurons? Moreover, this study focuses on melanoma tumors. Future studies should explore what is the role of sensory neurons in the development of other solid tumors.

**Fig. 10:**
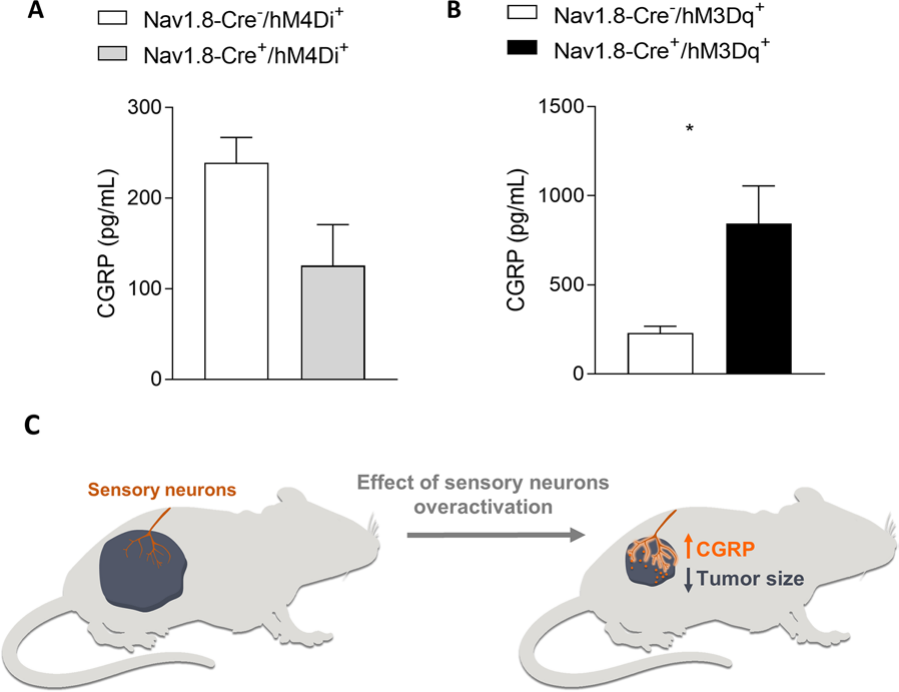
Increase in sensory neurons activity leads to increase in intra-tumoral calcitonin gene-related peptide (CGRP). CGRP concentration was measured in tumor samples from Nav1.8-Cre + /hM4Di + and Nav1.8-Cre-/hM4Di + animals (**A**) and from Nav1.8-Cre + /hM3Dq + and Nav1.8-Cre-/hM3Dq + mice (**B**). (**C**) Schematic representation of the association between intra-tumoral concentration of CGRP and tumor size. (n = 5). Data are shown as mean ± SEM

A variety of factors secreted from sensory neurons may be implicated in the regulation of the melanoma microenvironment described here [18]. The overactivation of sensory nerve fibers may induce the release of neuropeptides, such as substance P, CGRP, VIP, GRP, neurokinin A, neurokinin B, neuropeptide Y (NPY) and adrenomedullin, which have been shown to interact directly with several components within the tumor microenvironment, including cancer cells, immune cells, endothelial cells and others [4, 6, 17, 25, 26, 30, 35, 61, 80, 81, 93, 95, 117, 120, 142, 143, 148]. We detected an increase in intra-tumoral CGRP after overactivation of sensory neurons (Fig. 10). However, it remains unknown whether the decrease in the tumor size after sensory neurons overactivation is due to this increase in the intra-tumoral concentration of CGRP. Future studies will need to genetically eliminate specific receptors for this and other neuropeptides (such as receptor activity-modifying protein 1 (RAMP1) for CGRP) from different cellular components of the tumor microenvironment to reveal whether those communications are relevant for melanoma outcomes.

## Conclusion

In conclusion, this work identifies sensory neurons overactivation as a potential strategy for blocking melanoma progression and improving patient outcomes. We also anticipate that drugs that reduce sensory neurons hyperexcitability used for analgesic treatment may cause undesired effects in cancer patients and need to be carefully evaluated in pre-clinical models of cancer before using them in these patients [34, 125]. Moreover, the relationship between sensory hyperexcitability and cancer outcomes is likely to inform studies of other cancers that are also infiltrated by sensory neurons.

## Supplementary Information

Additional file 1. Gate strategy for regulatory markers. Representative contour plots showing proportion of CTLA-4 and PD-1 (top to bottom) in CD4+ and CD8+ (right to left) T viable lymphocytes within CD45+ alive cells from tumor infiltrate.

Additional file 2. Interactions among genes related to angiogenesis which are overexpressed in SKCM patients presenting worse prognosis (dead vs. alive).

Additional file 3. Inferred proportion of immune infiltrated cells in SKCM patients from the TCGA cohort.

Additional file 4. Differences observed on inferred proportions of tumor-infiltrating CD4+ T cells, CD8+ T cells, dendritic cells and NK cells between samples of alive and dead SKCM patients from the TCGA cohort.

Additional file 5. Impact of Th17 immune response in melanoma. (A) Microarray analysis of skin samples from Melanoma (n=46) and healthy (n=16) individuals from GEO database: GSE15605 was analyzed by Phantasus [156] (https://genome.ifmo.ru/phantasus). Expression of Th17 immune response markers in melanoma samples, normalized to health samples, as Log2 Fold Change. (B) Survival curve from melanoma patients. The prognostic impact of IL17A expression in melanoma patients was evaluated using the R2: Genomics Analysis and Visualization Platform (http://r2.amc.nl). We evaluated the survival probability of patients with melanoma based on their tumor transcriptome (n = 214) [19]. High expression of IL17A in melanoma is correlated with increased patient survival. Differences were considered significant at *P* value < 0.05.

## Data Availability

Data will be made available on reasonable request.
