# Supplementary figures and images for: Chemogenetic modulation of sensory neurons reveals their regulating role in melanoma progression
Source: Acta Neuropathol Commun. 2021 Nov 16;9:183. doi: 10.1186/s40478-021-01273-9 (PMC8594104; doi:10.1186/s40478-021-01273-9)

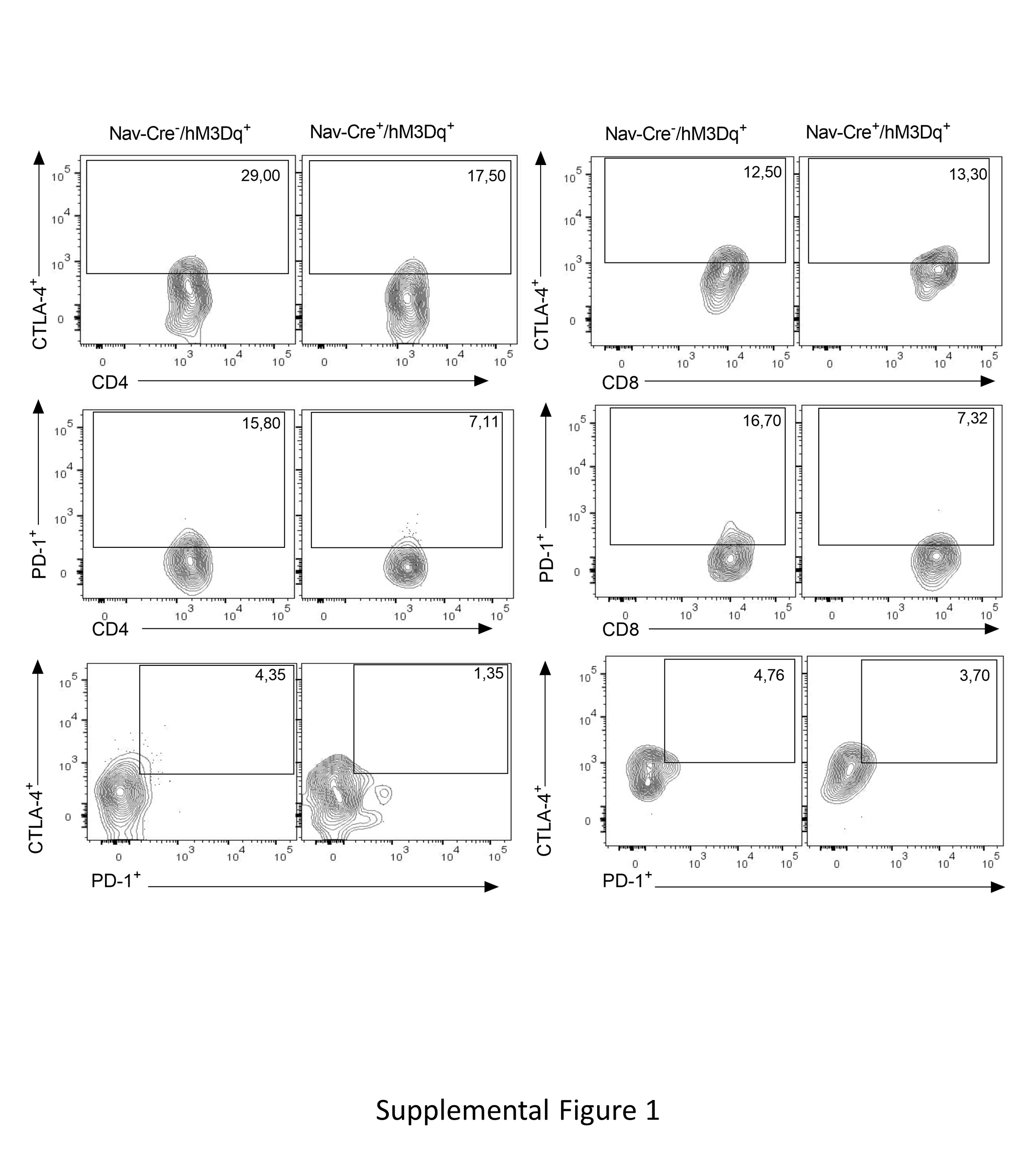

Supplement: Supplementary file 1 — Gate strategy for regulatory markers. Representative contour plots showing proportion of CTLA-4 and PD-1 (top to bottom) in CD4+ and CD8+ (right to left) T viable lymphocytes within CD45+ alive cells from tumor infiltrate. [file 40478_2021_1273_MOESM1_ESM.tif]

# ANGIOGENESIS

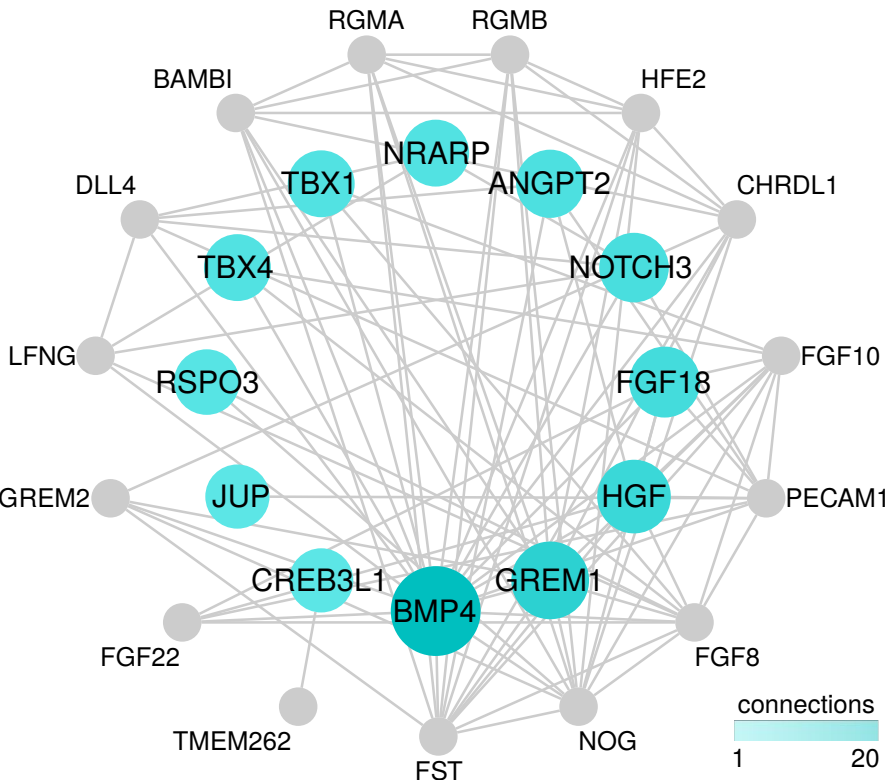

Supplement: Supplementary file 2 — Interactions among genes related to angiogenesis which are overexpressed in SKCM patients presenting worse prognosis (dead vs. alive). [file 40478_2021_1273_MOESM2_ESM.pdf]

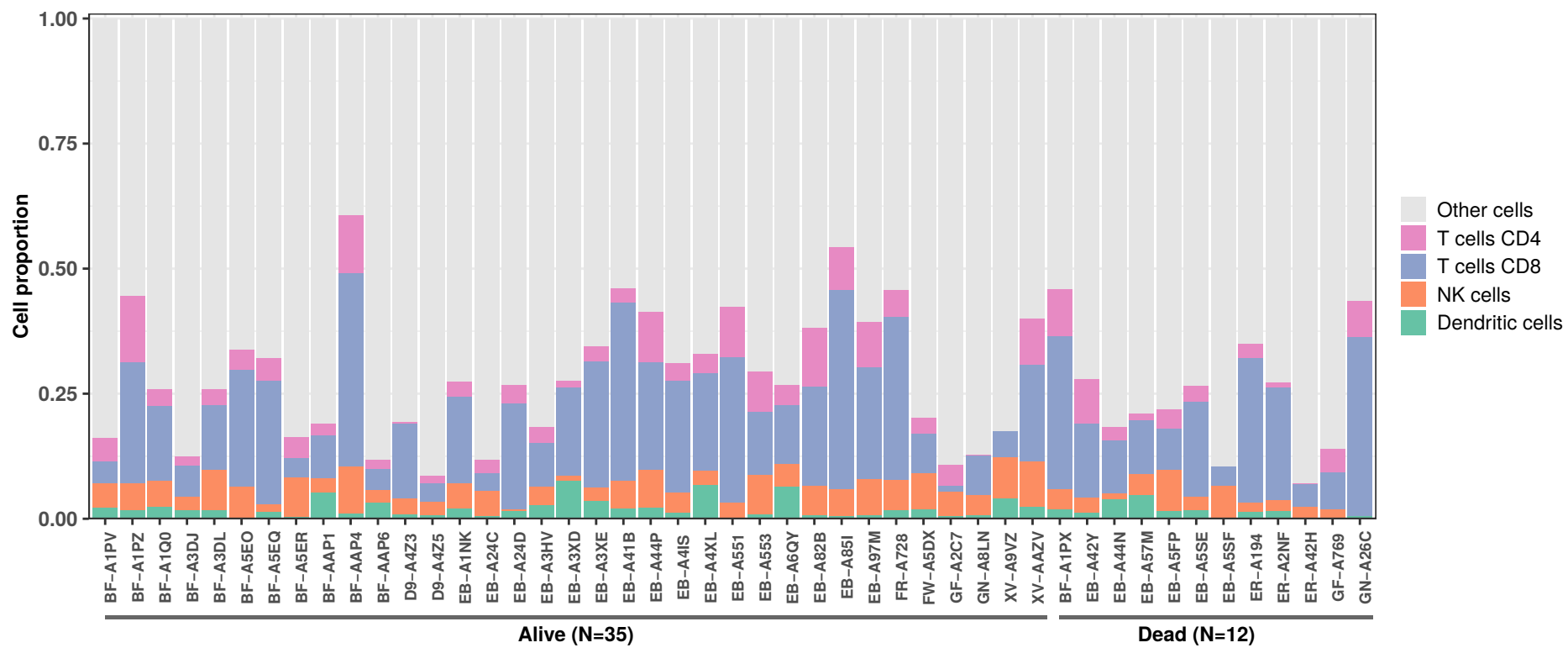

Supplement: Supplementary file 3 — Inferred proportion of immune infiltrated cells in SKCM patients from the TCGA cohort. [file 40478_2021_1273_MOESM3_ESM.pdf]

Cell proportion

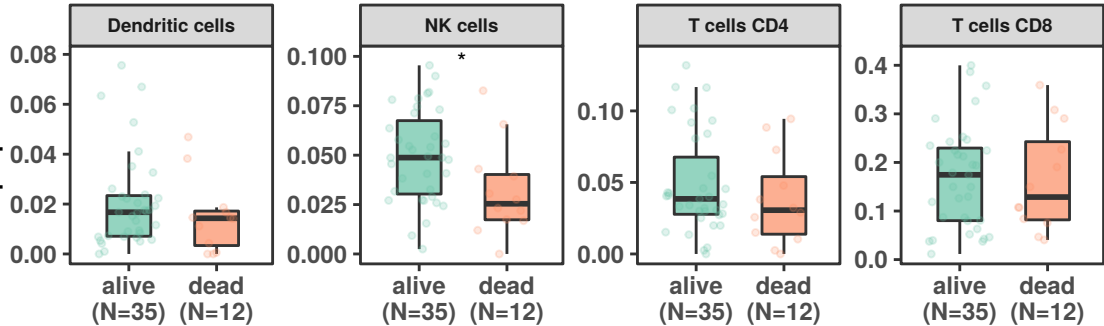

Supplement: Supplementary file 4 — Differences observed on inferred proportions of tumor-infiltrating CD4+ T cells, CD8+ T cells, dendritic cells and NK cells between samples of alive and dead SKCM patients from the TCGA cohort. [file 40478_2021_1273_MOESM4_ESM.pdf]

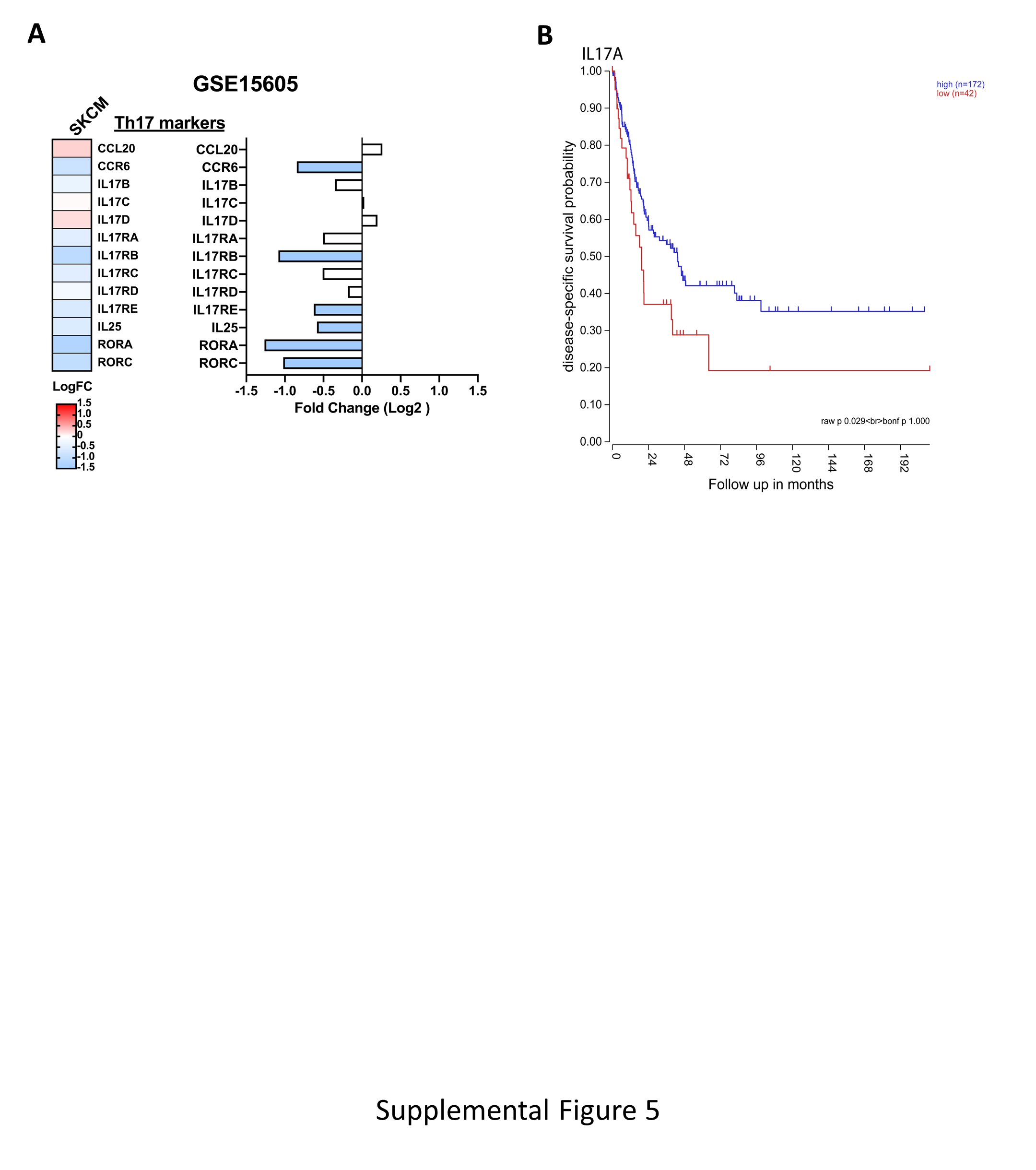

Supplement: Supplementary file 5 — Impact of Th17 immune response in melanoma. (A) Microarray analysis of skin samples from Melanoma (n=46) and healthy (n=16) individuals from GEO database: GSE15605 was analyzed by Phantasus [156] (https://genome.ifmo.ru/phantasus). Expression of Th17 immune response markers in melanoma samples, normalized to health samples, as Log2 Fold Change. (B) Survival curve from melanoma patients. The prognostic impact of IL17A expression in melanoma patients was evaluated using the R2: Genomics Analysis and Visualization Platform (http://r2.amc.nl). We evaluated the survival probability of patients with melanoma based on their tumor transcriptome (n = 214) [19]. High expression of IL17A in melanoma is correlated with increased patient survival. Differences were considered significant at P value < 0.05. [file 40478_2021_1273_MOESM5_ESM.tif]
